# Insights from daratumumab use in highly sensitized pediatric heart transplant candidates and recipients: A single-center institutional experience and outcomes

**DOI:** 10.1016/j.jhlto.2025.100346

**Published:** 2025-07-17

**Authors:** Majid Husain, Carrie Butler, Ashley M. Fan, Nancy J. Halnon, Leigh C. Reardon, Juan C. Alejos

**Affiliations:** a Division of Pediatric Cardiology, Department of Pediatrics, UCLA Mattel Children’s Hospital, Los Angeles, California; b Department of Pathology and Laboratory Medicine, David Geffen School of Medicine, UCLA Immunogenetics Center, Los Angeles, California; c Department of Pharmacy, UCLA Medical Center, Los Angeles, California; d Division of Cardiology, Department of Medicine, Ahmanson/UCLA Adult Congenital Heart Disease Center, Los Angeles, California

**Keywords:** allosensitization, daratumumab, desensitization, pediatric heart transplant, complex congenital heart disease

## Abstract

Allosensitization remains a significant obstacle for highly sensitized pediatric heart transplant candidates and recipients, particularly in the era of complex congenital heart disease palliations. Current desensitization strategies are limited, have variable efficacy, and lack durable response. Daratumumab, a monoclonal antibody targeting CD38 which is highly present on plasma cells and natural killer cells, is a novel and emerging option that has been successfully used for desensitization before heart transplantation in adults and to treat antibody-mediated rejection in solid organ recipients. However, there are limited data of its use in the pediatric population. We present our institution’s complete experience with daratumumab in 4 highly sensitized patients with reasonable tolerability and encouraging response.

## Background

Allosensitization remains a significant barrier for transplantation. Pre transplantation, allosensitization contributes to increased waitlist time and mortality, especially for highly sensitized candidates. Post transplantation, it increases the risk for antibody-mediated rejection (AMR) and has been associated with poor transplant outcomes.^1^ Current desensitization strategies are limited, have variable efficacy, and lack a durable response.^2^, ^3^

Daratumumab, a human 1gG1k monoclonal antibody which targets CD38—a transmembrane glycoprotein that is present on plasma cells and natural killer cells, is a novel and emerging therapy.^4^ Its mechanism of action makes it an attractive treatment option.^4^ It has been successfully used for desensitization before solid organ transplantation and to treat AMR in adults.^5^, ^6^ There are limited data of its use in the pediatric population,^7^, ^8^, ^9^ especially in those with significant allosensitization due to interventions for their complex congenital heart disease (CCHD).

Herein, we present our institutional protocol (Table 1), total experience thus far, and outcomes in 4 highly sensitized patients with reasonable tolerability and encouraging response. The cases are summarized in Table 2. Additional relevant details are provided below.

**Table 1:** Institutional Protocol for Administration of Daratumumab

**Week 1**		**Week 2**	

Day 1	• Premedications^a^, HSV prophylaxis^b^, and HBV screening^c^ • Split dose: 8 mg/kg/dose • Infusion rate: 25 ml/h	Day 1	*If infusion reaction/concerns from week 1, then repeat split or diluted dose* If no infusion reaction or concerns from Week 1: • Premedications (1 h before) • Full dose: 16 mg/kg/dose • Infusion rate: Start at 25 ml/h, increase by 25 ml/h to max rate of 100 ml/h
Day 2	• Premedications (1 h before) • Split dose: 8 mg/kg/dose • Infusion rate: Infuse at 25 ml/h for the first hour. If no infusion reactions occur, may increase the rate by 25 ml/h every hour (maximum rate: 100 ml/h) • Reduce the infusion rate for mild-moderate infusion reactions; permanently discontinue therapy for an anaphylactic or life-threatening infusion reaction and treat with appropriate emergency care	Day 2	Split dose OR post medication (based on week 1 tolerance)
Day 3 and 4	Post-medication: Methylprednisolone 0.5 mg/kg PO	Day 3	Post medication
Day 7	Check single antigen panel + IgG/subclasses • Give IVIG 0.4 g/kg for hypogammaglobulinemia	Day 7	Check single antigen panel + IgG/subclasses • Give IVIG 0.4 g/kg for hypogammaglobulinemia
			**Week 3-8 (and onward)**
			Same regimen as week 2

First dose should ideally be administered in the inpatient setting for close monitoring of infusion reactions. For the remainder of doses, the settings (inpatient vs outpatient), total number of doses, and spacing between doses is up to the discretion of the clinical team. Decision should be made in consultation with the immunogenetics team.

NOTE: For dosing, use actual body weight, rounded to nearest 100 mg, in 500 ml volume (unless specified by pharmacy).

The protocol was adapted from various sources and developed in collaboration with our pharmacy and immunogenetics teams: (A) Daratumumab product monograph—Janssen Inc. (B) McKeage, K. Daratumumab: first global approval. *Drugs* 76, 275-281 (2016). (C) Palumbo A, Chanan-Khan A, Weisel K, et al. Daratumumab, Bortezomib, and Dexamethasone for multiple myeloma. *N Engl J Med.* 2016;375(8):754-766. (D) Kwun J, Matignon M, Manook M, et al. Daratumumab in sensitized kidney transplantation: potentials and limitations of experimental and clinical use*. J Am Soc Nephrol.* 2019;30(7):1206-1219. (E) Aguilera Agudo C, Gómez Bueno M, Krsnik Castello I. Daratumumab for antibody-mediated rejection in heart transplant-a novel therapy: successful treatment of antibody-mediated rejection. *Transplantation*. 2021;105(3):e30-e31.

a Premedications (1 hour before infusion): Methylprednisolone 1 mg/kg IV, Acetaminophen 15 mg/kg PO, Diphenhydramine 1 mg/kg PO.

b HSV Prophylaxis: Acyclovir within 7 days of starting treatment and for 3 months following the end of treatment.

c HBV screening before starting treatment (due to reports of fatal HBV reactivation).

**Table 2:** Summary of Cases Highlighting Cardiac Diagnosis, Predaratumumab Therapies, Indication for Daratumumab Use, Age at Initial Dose, Total Number of Doses, Adverse Effects, cPRA, Outcomes, and Time to Transplantation (if Applicable)

Case	Diagnosis	Predaratumumab therapies	Daratumumab indication	Age at initial dose (years)	Total doses	Adverse effects	cPRA (all) Pre-Dara^a^	cPRA (all) Pre-OHT^a^	Outcome postdaratumumab	Time from daratumumab to OHT
1	HLHS s/p failed stage II palliation	IVIG, rituximab	Pretransplant desensitization (class I and II HLA, including C1q)	4	52 *(IP)*	None	99.99%	94.34%	Reduction in titers 78.77%^b^	n/a – waitlist
2	Shone’s Complex s/p biventricular repair	IVIG, TPE, rituximab, bortezomib, tocilizumab	Pretransplant desensitization (class I and II HLA, including C1q) Post-transplant DSA, pAMR-2	21	41	None	99.99%	94.56%	Successful transplantation across 4 HLA-DSA^c^ (negative CDC/Flow XM) Reduction in DSA	18 months
3	Repaired TOF/PA w/MAPCAS	IVIG, TPE	Pretransplant desensitization (class I and II HLA, including C1q)	9	8	Neutropenia^d^	99.99%	69.23%	Successful transplantation^e^ (negative CDC/Flow XM)	20 weeks
4	Idiopathic dilated cardiomyopathy	Steroids, IVIG, rituximab	Post-transplant DSA, pAMR-2	18	11 *(IP)*	Tachycardia, tightness in throat^f^	n/a		Reduction in DSA Resolution of pAMR-2	n/a

C1q, complement (C1q)-binding antibodies; CDC, complement-dependent cytotoxic; DSA, donor-specific antibodies; HLA, human leukocyte antigens; HLHS, hypoplastic left heart syndrome; IP: in-progress—currently receiving doses with serial serum surveillance and immunogenetics guidance; IVIG, intravenous immunoglobulins; MAPCAS, major aortopulmonary collaterals

; PA, pulmonary atresia; pAMR, pathologic antibody-mediated rejection; OHT, orthotopic heart transplant; TOF, tetralogy of fallot; TPE, therapeutic plasma exchange; XM, crossmatch.

a cPRA for all HLA class I and II antibodies.

b cPRA with unacceptable antigens listed as “avoids” in UNET (HLA MFI > 8,000: class I [A23, A24, B44, B45] and II [DR11, DR13, DR14, DR17, and DR18]).

c HLA-DSA: B71, DPA1*01, DP401, and DQ6 with all MFI<3,000 (historic DSA B38, Cw12, and Cw8).

d Neutropenia was likely multifactorial and partly related to Evans Syndrome.

e Death on POD#10 from gram-negative sepsis from suspected bacterial translocation versus contamination of mediastinal incision and chest tube sites from the proximal gastrostomy tube.

f Self-limited symptoms in the setting of patient’s anxiety and sensory integration disorder. Infusion rate decreased per protocol.

### Case series

**Figure 1:**
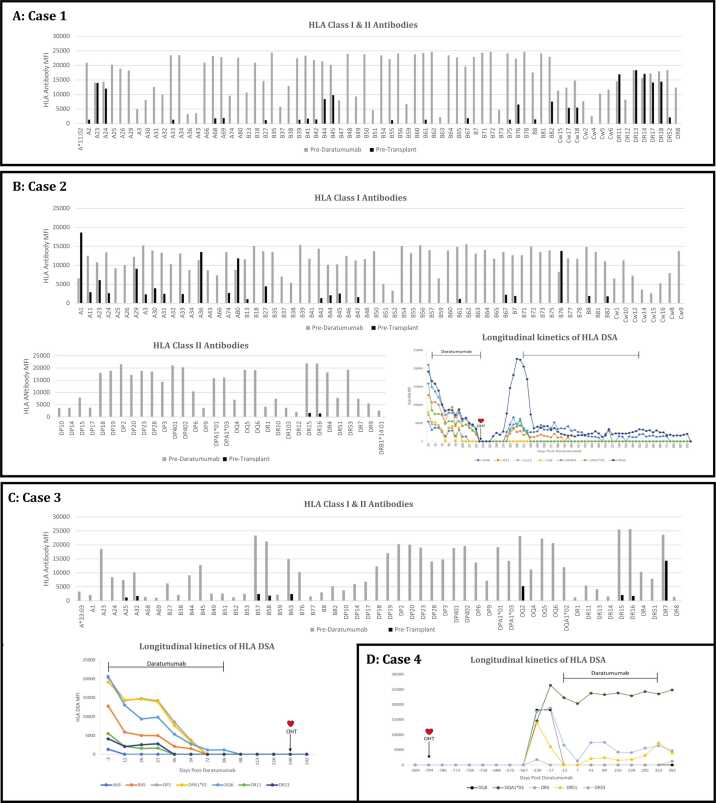
Changes in MFI levels are highlighted as predaratumumab (gray) and pretransplant (black). (A) Case 1: Bar graph showing changes in MFI level of HLA class I and II antibodies. (B) Case 2: Bar graph showing changes in MFI level of HLA class I and II antibodies. Line graph showing longitudinal kinetics in MFI level of HLA-DSA (y-axis) over time in days post daratumumab (x-axis). Black bars represent the timeframes daratumumab was administered. (C) Case 3: Bar graph showing changes in MFI level of HLA class I and II antibodies. Line graph showing longitudinal kinetics in MFI level of HLA-DSA (y-axis) over time in days post daratumumab (x-axis). Black bars represent the timeframes daratumumab was administered. (D) Case 4: Line graph showing longitudinal kinetics in MFI level of HLA-DSA (y-axis) over time in days post daratumumab (x-axis). Black bars represent the timeframes daratumumab was administered. DSA, donor-specific antibodies; HLA, human leukocyte antigens; MFI, mean fluorescence intensity; OHT, orthotopic heart transplant.

*Case 1* (Figure 1A): A now 6-year-old female was listed for ABO-compatible orthotopic heart transplant (OHT) due to failed stage II palliation for hypoplastic left heart syndrome. She was highly sensitized and treated with conventional therapies with only a modest response. During an admission for refractory arrhythmias with resultant cardiac arrest requiring venoarterial extracorporeal membrane oxygenation, she underwent therapeutic plasma exchange (TPE) and initiation of daratumumab for aggressive desensitization. She is currently receiving biweekly dosing per immunogenetics recommendations following her initial 8-week course with an overall moderate reduction in class I and II HLA antibodies. Her current cPRA is 78.77% for those listed as unacceptable antigens. She is outpatient, per family preference, and remains listed as status 1B.

*Case 2* (Figure 1B): A now 23-year-old male underwent OHT due to end-stage biventricular heart failure after repair of Shone's Complex with a valved left ventricle to descending aorta conduit as secondary source of systemic blood flow. He was heavily sensitized before transplantation due to extensive homograft material and received aggressive desensitization with no significant reduction in antibody titers. This was followed by daratumumab with a more robust response. He was successfully transplanted across 4 donor-specific antibodies (DSAs) with a negative cytotoxic and flow crossmatch. Induction therapy included ATG and TPE. However, he had rebound of DQ6 class II antibody to >20,000 MFI post transplant with pathologic AMR-2 (pAMR) though with normal graft function. Consequently, he underwent surgical removal of the retained conduit and repeat treatment with daratumumab resulting in significant reduction of DQ6 class II antibodies to <5,000 MFI. He was continued on weekly doses of daratumumab, which were ultimately spaced to monthly for a total course of 1 year. He received a total of 41 doses. He continues to have pAMR-2 due to a combination of low-level DSA, non-DSA HLA, and non-HLA antibodies. He was treated with a combination of ATG, TPE, IVIG, and rituximab and remains on quadruple maintenance immunosuppression. Clinically, he remains asymptomatic, has normal graft function on surveillance echocardiograms, and has no evidence of coronary artery vasculopathy by angiography.

*Case 3* (Figure 1C): A 9-year-old female was listed for combined ABO-compatible OHT and kidney transplant in the setting of progressive systolic heart failure requiring intravenous milrinone after repair of Tetralogy of Fallot with pulmonary atresia/major aortopulmonary collaterals and end-stage renal disease requiring dialysis. She also had DiGeorge Syndrome and a profound transfusion requirement for severe epistaxis from thrombocytopenia related to Evans Syndrome. As a result, she was broadly sensitized. Daratumumab was initiated after trailing TPE, IVIG, and rituximab. She completed her initial course of 8 weekly doses. No additional doses were administered due to neutropenia, likely exacerbated by her underlying Evans Syndrome. She had a favorable reduction in antibody titers, allowing for a negative cytotoxic and flow crossmatch and successful OHT. No antibodies were crossed. Due to perioperative complications, the kidney transplant was deferred. She ultimately died on postoperative day 10 due to fulminant gram-negative sepsis.

*Case 4* (Figure 1D): An 18-year-old male with a history of OHT due to idiopathic dilated cardiomyopathy was found to have pAMR-2 and multiple de Novo DSA approximately 18 months post transplantation. He was treated with a combination of steroids, IVIG, and rituximab. Due to persistent pathologic AMR and DSA, he was then treated with TPE and daratumumab with a significant decrease in antibody titers. He received his initial 2 doses in the inpatient setting and currently receives monthly outpatient infusions. To date he has received 11 doses. His surveillance endomyocardial biopsies have been negative for pAMR. He is maintained on tacrolimus, sirolimus, and low-dose steroids as part of his chronic immunosuppressive regimen.

## Discussion

Allosensitization is a significant barrier for transplantation. It is magnified in the pediatric and congenital population due to the routine use of biological, nonbiological, and bioprosthetic materials; multiple surgical palliations; catheter-based interventions; and the need for frequent transfusions. Given the limitations of current therapies, daratumumab offers a promising alternative or use as adjunctive therapy.^6^

As collectively highlighted by our 4 cases, daratumumab is well-tolerated across a variety of ages without major adverse effects and yields a reasonable reduction in antibody titers and cPRA, thereby increasing the probability of a negative crossmatch. None had reactivation of the hepatitis B virus. Nonetheless, longitudinal data is required to accurately determine the duration of an optimal course, the durability of response to the therapy, and long-term consequences. Specifically, given the reduction in regulatory T and B cells subsets and consequent increase in CD4-positive cytotoxic T cells and CD8-positive helper T cells, there is concern for T cell-mediated rejection.^10^ Reassuringly, neither of our 2 post-transplant patients have had biopsy proven cellular rejection after receiving treatment.

## Conclusion

Daratumumab is a feasible option for desensitization therapy and the treatment of DSA and AMR in the highly sensitized pediatric heart transplant population. It appears to be well-tolerated with minimal side effects. Additional studies are needed to determine the duration of the optimal course, durability of response, long-term consequences, and efficacy with combination therapies.

## Disclosure statement

The authors declare that they have no known competing financial interests or personal relationships that could have appeared to influence the work reported in this paper.

The authors are thankful to the patients, their families, and the entire clinical and nonclinical team for their efforts in advancing our understanding of this novel therapy.

This research did not receive any specific grant from funding agencies in the public, commercial, or not-for-profit sectors.

The work was presented and recognized at the International Society of Heart and Lung Annual Conference in 2024 during the Early Career & Trainee Clinical Case Dilemmas: The Best of the Best Award Session.

## Data Availability

Data from the cases reported are available from the corresponding author upon request.
