# Supplementary material for: Insights from daratumumab use in highly sensitized pediatric heart transplant candidates and recipients: A single-center institutional experience and outcomes
Source: JHLT Open. 2025 Jul 17;10:100346. doi: 10.1016/j.jhlto.2025.100346 (PMC12354783; doi:10.1016/j.jhlto.2025.100346)
Supplement: Supplementary file 1 — Supplementary material [file mmc1.docx]

*Supplemental figure:*

Figure 2 – Longitudinal kinetics in MFI level of HLA Class I and II antibodies (y-axis) over time in days post-daratumumab (x-axis).

HLA: Human leukocyte antigens; MFI – Mean fluorescence intensity; OHT – Orthotopic heart transplant
