# Supplementary material for: Insights from daratumumab use in highly sensitized pediatric heart transplant candidates and recipients: A single-center institutional experience and outcomes
Source: JHLT Open. 2025 Jul 17;10:100346. doi: 10.1016/j.jhlto.2025.100346 (PMC12354783; doi:10.1016/j.jhlto.2025.100346)
Supplement: Supplementary file 2 — Supplementary material [file mmc2.pptx]

## Slide 1
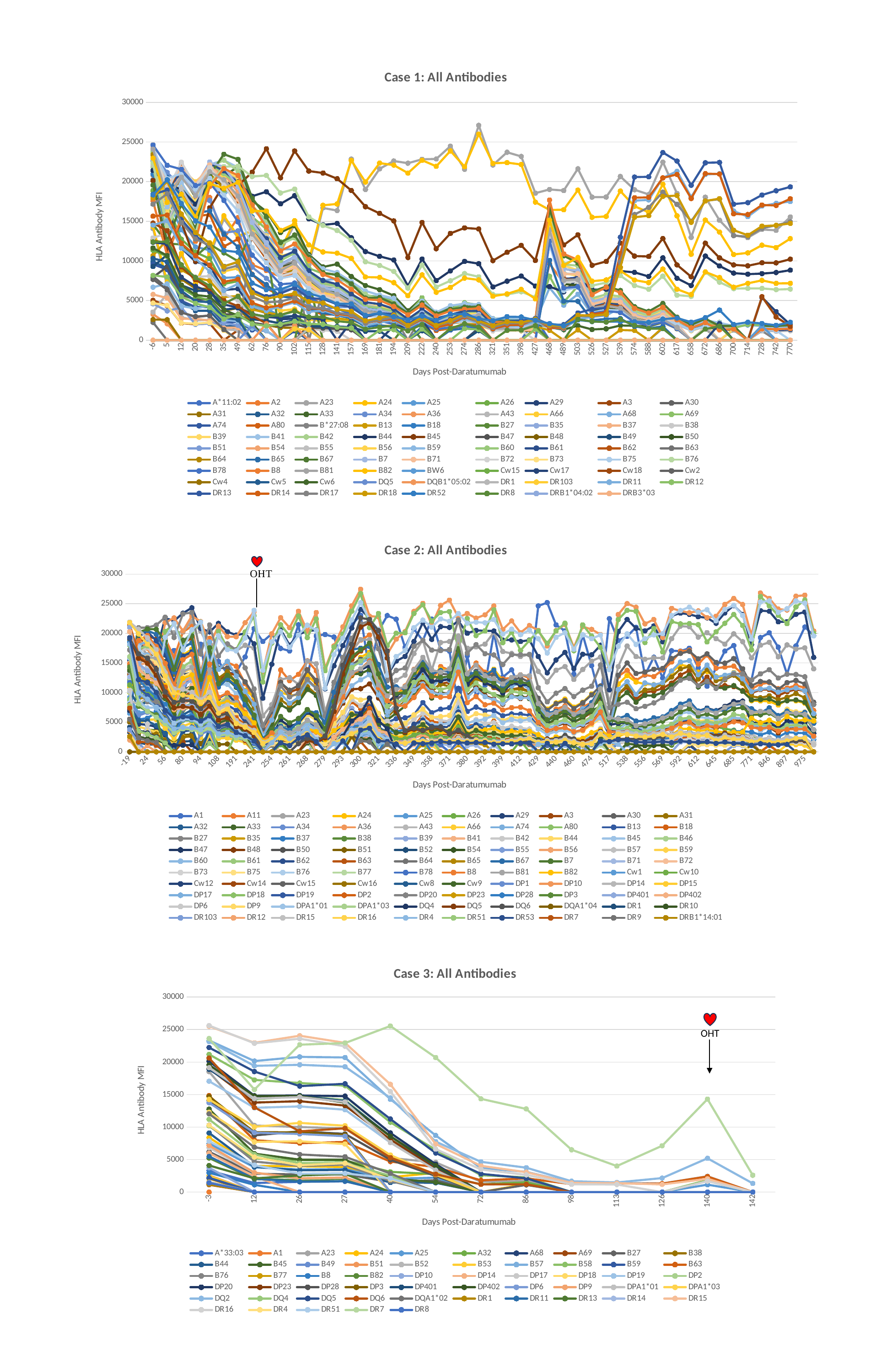

### Chart: Case 1: All Antibodies
| Category | A*11:02 | A2 | A23 | A24 | A25 | A26 | A29 | A3 | A30 | A31 | A32 | A33 | A34 | A36 | A43 | A66 | A68 | A69 | A74 | A80 | B*27:08 | B13 | B18 | B27 | B35 | B37 | B38 | B39 | B41 | B42 | B44 | B45 | B47 | B48 | B49 | B50 | B51 | B54 | B55 | B56 | B59 | B60 | B61 | B62 | B63 | B64 | B65 | B67 | B7 | B71 | B72 | B73 | B75 | B76 | B78 | B8 | B81 | B82 | BW6 | Cw15 | Cw17 | Cw18 | Cw2 | Cw4 | Cw5 | Cw6 | DQ5 | DQB1*05:02 | DR1 | DR103 | DR11 | DR12 | DR13 | DR14 | DR17 | DR18 | DR52 | DR8 | DRB1*04:02 | DRB3*03 |
|---|---|---|---|---|---|---|---|---|---|---|---|---|---|---|---|---|---|---|---|---|---|---|---|---|---|---|---|---|---|---|---|---|---|---|---|---|---|---|---|---|---|---|---|---|---|---|---|---|---|---|---|---|---|---|---|---|---|---|---|---|---|---|---|---|---|---|---|---|---|---|---|---|---|---|---|---|---|---|---|---|
| -6 | 0.0 | 20846.0 | 14088.0 | 14337.0 | 20236.0 | 18887.0 | 18216.0 | 5029.0 | 8049.0 | 12600.0 | 9930.0 | 23515.0 | 23526.0 | 3266.0 | 3545.0 | 20901.0 | 23184.0 | 22836.0 | 9571.0 | 22634.0 | 0.0 | 10668.0 | 20913.0 | 14620.0 | 24369.0 | 5768.0 | 12897.0 | 22411.0 | 23326.0 | 21876.0 | 21413.0 | 20152.0 | 7934.0 | 23928.0 | 9298.0 | 23771.0 | 4580.0 | 23371.0 | 22150.0 | 24092.0 | 6677.0 | 23793.0 | 24225.0 | 24617.0 | 2240.0 | 23415.0 | 22682.0 | 19574.0 | 22859.0 | 24279.0 | 24682.0 | 4677.0 | 24133.0 | 22363.0 | 24608.0 | 17570.0 | 24094.0 | 22952.0 | 0.0 | 11350.0 | 12338.0 | 14765.0 | 7671.0 | 2599.0 | 10312.0 | 11625.0 | 0.0 | 0.0 | 0.0 | 0.0 | 14508.0 | 8172.0 | 18386.0 | 15645.0 | 17156.0 | 17957.0 | 18334.0 | 12388.0 | 0.0 | 0.0 |
| 5 | 0.0 | 19045.0 | 6850.0 | 7331.0 | 17952.0 | 17077.0 | 16743.0 | 4348.0 | 6606.0 | 12235.0 | 9068.0 | 19533.0 | 21155.0 | 2071.0 | 5895.0 | 18556.0 | 16637.0 | 16296.0 | 8913.0 | 18777.0 | 0.0 | 12687.0 | 13765.0 | 12196.0 | 18638.0 | 5311.0 | 13525.0 | 16095.0 | 16799.0 | 13913.0 | 19716.0 | 11398.0 | 9280.0 | 18674.0 | 10763.0 | 18841.0 | 3688.0 | 18094.0 | 14716.0 | 19207.0 | 7111.0 | 17970.0 | 18971.0 | 18583.0 | 0.0 | 17616.0 | 20538.0 | 12514.0 | 17522.0 | 19501.0 | 18849.0 | 4387.0 | 20380.0 | 14408.0 | 22058.0 | 10656.0 | 17959.0 | 17388.0 | 0.0 | 10573.0 | 12134.0 | 13815.0 | 6366.0 | 2584.0 | 9872.0 | 10662.0 | 0.0 | 0.0 | 0.0 | 0.0 | 15071.0 | 7980.0 | 18940.0 | 15791.0 | 18468.0 | 20057.0 | 20239.0 | 12436.0 | 0.0 | 0.0 |
| 12 | 0.0 | 18619.0 | 10737.0 | 10936.0 | 13926.0 | 12741.0 | 11631.0 | 2150.0 | 3522.0 | 7692.0 | 4837.0 | 20957.0 | 19354.0 | 0.0 | 2902.0 | 14856.0 | 18422.0 | 18605.0 | 4963.0 | 18493.0 | 0.0 | 8863.0 | 19309.0 | 12069.0 | 21419.0 | 2691.0 | 9280.0 | 19584.0 | 19914.0 | 18260.0 | 19000.0 | 14919.0 | 5727.0 | 21521.0 | 6939.0 | 21481.0 | 4116.0 | 20831.0 | 19148.0 | 21539.0 | 4311.0 | 20581.0 | 21227.0 | 22264.0 | 0.0 | 20569.0 | 18166.0 | 17079.0 | 20810.0 | 21691.0 | 22456.0 | 2174.0 | 21012.0 | 17671.0 | 21552.0 | 15437.0 | 20573.0 | 18410.0 | 0.0 | 6601.0 | 7930.0 | 9243.0 | 3486.0 | 0.0 | 6226.0 | 6951.0 | 0.0 | 0.0 | 0.0 | 0.0 | 11648.0 | 5481.0 | 15223.0 | 12218.0 | 14850.0 | 15904.0 | 17233.0 | 9508.0 | 0.0 | 0.0 |
| 20 | 0.0 | 16669.0 | 7093.0 | 7182.0 | 11766.0 | 11096.0 | 9906.0 | 2000.0 | 2912.0 | 6525.0 | 3714.0 | 18018.0 | 17258.0 | 0.0 | 2491.0 | 13059.0 | 15811.0 | 15454.0 | 4174.0 | 15608.0 | 0.0 | 7884.0 | 16860.0 | 10566.0 | 19385.0 | 2715.0 | 7982.0 | 17465.0 | 16902.0 | 15296.0 | 17166.0 | 9961.0 | 5122.0 | 18726.0 | 6240.0 | 18758.0 | 1998.0 | 18184.0 | 16020.0 | 19993.0 | 3985.0 | 18595.0 | 18876.0 | 19563.0 | 0.0 | 18333.0 | 16033.0 | 14111.0 | 18498.0 | 19698.0 | 19483.0 | 2078.0 | 19101.0 | 13477.0 | 19501.0 | 12477.0 | 18254.0 | 15532.0 | 0.0 | 5502.0 | 6791.0 | 7625.0 | 2983.0 | 0.0 | 4909.0 | 5709.0 | 0.0 | 0.0 | 0.0 | 0.0 | 10317.0 | 4643.0 | 13199.0 | 10656.0 | 12753.0 | 13594.0 | 15044.0 | 7901.0 | 0.0 | 0.0 |
| 28 | 0.0 | 17999.0 | 10618.0 | 11024.0 | 11351.0 | 10287.0 | 9401.0 | 2122.0 | 3123.0 | 6474.0 | 3709.0 | 22007.0 | 17643.0 | 0.0 | 2561.0 | 12312.0 | 20300.0 | 19835.0 | 4171.0 | 15864.0 | 0.0 | 7500.0 | 21485.0 | 12110.0 | 22477.0 | 2457.0 | 8087.0 | 22299.0 | 22159.0 | 21798.0 | 19861.0 | 16688.0 | 5163.0 | 20434.0 | 6205.0 | 21433.0 | 2100.0 | 21764.0 | 21916.0 | 21527.0 | 4341.0 | 22297.0 | 22301.0 | 22024.0 | 0.0 | 21008.0 | 16318.0 | 20824.0 | 22397.0 | 22107.0 | 21766.0 | 2215.0 | 20437.0 | 19713.0 | 19876.0 | 19394.0 | 21230.0 | 19737.0 | 0.0 | 4995.0 | 6100.0 | 6513.0 | 3090.0 | 0.0 | 4789.0 | 5487.0 | 0.0 | 0.0 | 0.0 | 0.0 | 8664.0 | 4132.0 | 11728.0 | 9482.0 | 11959.0 | 12238.0 | 14280.0 | 6852.0 | 0.0 | 0.0 |
| 35 | 0.0 | 12823.0 | 15297.0 | 15695.0 | 7857.0 | 7089.0 | 6335.0 | 0.0 | 1290.0 | 3694.0 | 1748.0 | 20722.0 | 13538.0 | 0.0 | 1352.0 | 8680.0 | 18241.0 | 18843.0 | 2190.0 | 11822.0 | 0.0 | 5450.0 | 21806.0 | 11014.0 | 21104.0 | 1568.0 | 5836.0 | 21584.0 | 22703.0 | 22711.0 | 19766.0 | 19594.0 | 3248.0 | 19681.0 | 3847.0 | 19910.0 | 2156.0 | 21000.0 | 21579.0 | 20581.0 | 2556.0 | 21027.0 | 21358.0 | 19843.0 | 0.0 | 19117.0 | 13370.0 | 23457.0 | 21895.0 | 20035.0 | 19568.0 | 1216.0 | 18286.0 | 21878.0 | 17644.0 | 21653.0 | 20240.0 | 19163.0 | 0.0 | 3070.0 | 4506.0 | 4994.0 | 1347.0 | 0.0 | 2811.0 | 3844.0 | 0.0 | 0.0 | 0.0 | 0.0 | 6269.0 | 2674.0 | 9321.0 | 7374.0 | 8967.0 | 9284.0 | 11325.0 | 4592.0 | 0.0 | 0.0 |
| 49 | 0.0 | 15455.0 | 12944.0 | 13140.0 | 7945.0 | 7440.0 | 6478.0 | 1266.0 | 1671.0 | 4057.0 | 1812.0 | 21862.0 | 13799.0 | 0.0 | 1259.0 | 9065.0 | 20187.0 | 20832.0 | 2318.0 | 12756.0 | 0.0 | 5572.0 | 20250.0 | 9961.0 | 19430.0 | 1589.0 | 5034.0 | 20417.0 | 21785.0 | 21299.0 | 19957.0 | 19492.0 | 3278.0 | 17515.0 | 4064.0 | 17722.0 | 1875.0 | 18664.0 | 19866.0 | 17430.0 | 2397.0 | 19552.0 | 19861.0 | 17516.0 | 0.0 | 18025.0 | 11754.0 | 22811.0 | 19341.0 | 17774.0 | 16913.0 | 0.0 | 16283.0 | 21957.0 | 14927.0 | 20778.0 | 18168.0 | 19921.0 | 0.0 | 2941.0 | 4105.0 | 4291.0 | 0.0 | 0.0 | 2590.0 | 3082.0 | 0.0 | 0.0 | 0.0 | 0.0 | 7086.0 | 3233.0 | 9853.0 | 7687.0 | 9418.0 | 9903.0 | 11106.0 | 5509.0 | 0.0 | 0.0 |
| 62 | 0.0 | 9519.0 | 13835.0 | 13788.0 | 4613.0 | 4440.0 | 3982.0 | 0.0 | 0.0 | 2164.0 | 0.0 | 17595.0 | 9220.0 | 0.0 | 0.0 | 5578.0 | 14604.0 | 15565.0 | 1292.0 | 8308.0 | 13816.0 | 3868.0 | 15538.0 | 13816.0 | 14413.0 | 0.0 | 3170.0 | 14806.0 | 16601.0 | 16962.0 | 18157.0 | 21189.0 | 2192.0 | 12871.0 | 2634.0 | 12887.0 | 0.0 | 13821.0 | 15394.0 | 13537.0 | 1496.0 | 14261.0 | 14840.0 | 12853.0 | 0.0 | 13006.0 | 8285.0 | 17918.0 | 14848.0 | 13146.0 | 12875.0 | 0.0 | 11682.0 | 20645.0 | 10728.0 | 17064.0 | 13387.0 | 16284.0 | 0.0 | 0.0 | 3321.0 | 3066.0 | 0.0 | 0.0 | 0.0 | 2192.0 | 1805.0 | 0.0 | 0.0 | 0.0 | 4294.0 | 2058.0 | 6307.0 | 4713.0 | 5447.0 | 5995.0 | 7202.0 | 3271.0 | 0.0 | 0.0 |
| 76 | 0.0 | 8792.0 | 15454.0 | 15552.0 | 4004.0 | 3634.0 | 3410.0 | 0.0 | 0.0 | 1907.0 | 0.0 | 16240.0 | 7787.0 | 0.0 | 0.0 | 4708.0 | 13330.0 | 14013.0 | 1187.0 | 6526.0 | 11857.0 | 3480.0 | 12667.0 | 11857.0 | 12055.0 | 0.0 | 2595.0 | 13148.0 | 14775.0 | 15139.0 | 18727.0 | 24145.0 | 1929.0 | 10534.0 | 2443.0 | 11079.0 | 0.0 | 11410.0 | 12978.0 | 11268.0 | 1206.0 | 12539.0 | 12790.0 | 10214.0 | 0.0 | 10593.0 | 7018.0 | 15560.0 | 12312.0 | 10856.0 | 10551.0 | 0.0 | 9771.0 | 20777.0 | 8986.0 | 14276.0 | 11202.0 | 16288.0 | 0.0 | 0.0 | 2935.0 | 2116.0 | 0.0 | 0.0 | 0.0 | 0.0 | 0.0 | 2058.0 | 0.0 | 0.0 | 3731.0 | 1795.0 | 5498.0 | 4090.0 | 4780.0 | 5223.0 | 6375.0 | 2986.0 | 0.0 | 0.0 |
| 90 | 0.0 | 7999.0 | 11100.0 | 11361.0 | 3023.0 | 2882.0 | 2637.0 | 0.0 | 0.0 | 1584.0 | 0.0 | 13585.0 | 6196.0 | 0.0 | 0.0 | 3711.0 | 11468.0 | 12429.0 | 0.0 | 5635.0 | 9372.0 | 3187.0 | 10587.0 | 0.0 | 9593.0 | 0.0 | 1993.0 | 10641.0 | 12004.0 | 12081.0 | 17198.0 | 20479.0 | 1811.0 | 8142.0 | 2124.0 | 9151.0 | 0.0 | 8991.0 | 10411.0 | 8863.0 | 0.0 | 10050.0 | 10425.0 | 8242.0 | 0.0 | 8678.0 | 5530.0 | 12321.0 | 9424.0 | 8517.0 | 8086.0 | 0.0 | 7802.0 | 18565.0 | 7050.0 | 11265.0 | 8752.0 | 13855.0 | 0.0 | 0.0 | 2792.0 | 0.0 | 0.0 | 0.0 | 0.0 | 0.0 | 0.0 | 0.0 | 0.0 | 0.0 | 4261.0 | 2011.0 | 5894.0 | 4396.0 | 4874.0 | 5558.0 | 6413.0 | 3175.0 | 0.0 | 0.0 |
| 102 | 0.0 | 8304.0 | 14657.0 | 15079.0 | 3061.0 | 3030.0 | 2712.0 | 0.0 | 0.0 | 1568.0 | 0.0 | 14448.0 | 6568.0 | 0.0 | 0.0 | 3918.0 | 11484.0 | 13110.0 | 1166.0 | 5548.0 | 9948.0 | 3746.0 | 10942.0 | 0.0 | 10542.0 | 0.0 | 2083.0 | 11054.0 | 13264.0 | 12909.0 | 18218.0 | 23866.0 | 2022.0 | 8710.0 | 2516.0 | 9707.0 | 0.0 | 9589.0 | 11138.0 | 9484.0 | 0.0 | 10987.0 | 11223.0 | 8576.0 | 0.0 | 8773.0 | 5297.0 | 13258.0 | 10458.0 | 9178.0 | 8627.0 | 0.0 | 8169.0 | 19069.0 | 7204.0 | 12150.0 | 9738.0 | 14677.0 | 0.0 | 0.0 | 3079.0 | 0.0 | 0.0 | 0.0 | 0.0 | 0.0 | 0.0 | 1829.0 | 0.0 | 1425.0 | 4766.0 | 2381.0 | 6612.0 | 4951.0 | 5354.0 | 5920.0 | 7039.0 | 3646.0 | 0.0 | 0.0 |
| 115 | 0.0 | 5052.0 | 9517.0 | 10032.0 | 1924.0 | 1972.0 | 1937.0 | 0.0 | 0.0 | 0.0 | 0.0 | 10800.0 | 4368.0 | 0.0 | 0.0 | 2602.0 | 8174.0 | 9077.0 | 0.0 | 3920.0 | 0.0 | 2816.0 | 8799.0 | 7435.0 | 8054.0 | 0.0 | 1443.0 | 8582.0 | 10588.0 | 10384.0 | 15318.0 | 21334.0 | 1583.0 | 7280.0 | 1757.0 | 7550.0 | 0.0 | 7265.0 | 8503.0 | 7316.0 | 0.0 | 8534.0 | 8723.0 | 6530.0 | 0.0 | 6625.0 | 3991.0 | 10499.0 | 8149.0 | 7009.0 | 6507.0 | 0.0 | 6455.0 | 15555.0 | 5406.0 | 10183.0 | 7742.0 | 12007.0 | 0.0 | 0.0 | 2810.0 | 1872.0 | 0.0 | 0.0 | 1615.0 | 1322.0 | 0.0 | 0.0 | 1462.0 | 1326.0 | 3566.0 | 2056.0 | 5312.0 | 4029.0 | 4122.0 | 4871.0 | 5891.0 | 2928.0 | 0.0 | 0.0 |
| 128 | 0.0 | 5235.0 | 16678.0 | 17017.0 | 1701.0 | 1672.0 | 1712.0 | 0.0 | 0.0 | 0.0 | 0.0 | 9240.0 | 3836.0 | 0.0 | 0.0 | 2377.0 | 7557.0 | 8396.0 | 0.0 | 3208.0 | 6241.0 | 2651.0 | 6775.0 | 0.0 | 6479.0 | 0.0 | 1256.0 | 7255.0 | 8988.0 | 8549.0 | 14543.0 | 21090.0 | 1526.0 | 5834.0 | 1747.0 | 6199.0 | 0.0 | 5979.0 | 6925.0 | 5883.0 | 0.0 | 7161.0 | 7509.0 | 5327.0 | 0.0 | 5463.0 | 3453.0 | 8495.0 | 6696.0 | 5676.0 | 5499.0 | 0.0 | 5150.0 | 14480.0 | 4466.0 | 8294.0 | 6321.0 | 11128.0 | 0.0 | 0.0 | 2589.0 | 0.0 | 0.0 | 0.0 | 0.0 | 0.0 | 0.0 | 0.0 | 0.0 | 0.0 | 3755.0 | 2012.0 | 5194.0 | 3943.0 | 4044.0 | 4693.0 | 5394.0 | 2947.0 | 0.0 | 0.0 |
| 141 | 0.0 | 5026.0 | 16351.0 | 17185.0 | 1695.0 | 1666.0 | 1558.0 | 0.0 | 0.0 | 0.0 | 0.0 | 9600.0 | 4204.0 | 0.0 | 0.0 | 2531.0 | 7729.0 | 8249.0 | 0.0 | 3290.0 | 5980.0 | 2745.0 | 6651.0 | 0.0 | 6262.0 | 0.0 | 1122.0 | 6773.0 | 8529.0 | 8174.0 | 14729.0 | 20367.0 | 1413.0 | 5458.0 | 1731.0 | 5952.0 | 0.0 | 5676.0 | 6649.0 | 5611.0 | 0.0 | 6795.0 | 7056.0 | 5165.0 | 0.0 | 5123.0 | 3127.0 | 8241.0 | 6180.0 | 5447.0 | 5047.0 | 0.0 | 4749.0 | 13843.0 | 3975.0 | 7625.0 | 5803.0 | 10982.0 | 0.0 | 0.0 | 0.0 | 0.0 | 0.0 | 0.0 | 0.0 | 0.0 | 0.0 | 0.0 | 0.0 | 0.0 | 3181.0 | 1642.0 | 4526.0 | 3309.0 | 3460.0 | 3913.0 | 4591.0 | 2396.0 | 0.0 | 0.0 |
| 157 | 0.0 | 4562.0 | 22837.0 | 22672.0 | 1601.0 | 1452.0 | 1432.0 | 0.0 | 0.0 | 0.0 | 0.0 | 8035.0 | 4174.0 | 0.0 | 0.0 | 2512.0 | 7250.0 | 7236.0 | 0.0 | 3043.0 | 4941.0 | 2554.0 | 5507.0 | 0.0 | 5115.0 | 0.0 | 0.0 | 5926.0 | 7375.0 | 6838.0 | 12912.0 | 18883.0 | 1321.0 | 4144.0 | 1634.0 | 5131.0 | 0.0 | 4662.0 | 5575.0 | 4609.0 | 0.0 | 5681.0 | 5806.0 | 4364.0 | 0.0 | 4271.0 | 2782.0 | 7132.0 | 5180.0 | 4457.0 | 4219.0 | 0.0 | 4009.0 | 12589.0 | 3250.0 | 6433.0 | 4730.0 | 10326.0 | 0.0 | 0.0 | 2326.0 | 0.0 | 0.0 | 0.0 | 0.0 | 0.0 | 0.0 | 0.0 | 0.0 | 0.0 | 2975.0 | 1542.0 | 4161.0 | 3071.0 | 3191.0 | 3634.0 | 4590.0 | 2230.0 | 0.0 | 0.0 |
| 169 | 0.0 | 3157.0 | 18998.0 | 19944.0 | 0.0 | 0.0 | 0.0 | 0.0 | 0.0 | 0.0 | 0.0 | 6900.0 | 3041.0 | 0.0 | 0.0 | 2487.0 | 5586.0 | 5722.0 | 0.0 | 1997.0 | 3677.0 | 1883.0 | 4221.0 | 0.0 | 3716.0 | 0.0 | 0.0 | 4458.0 | 6209.0 | 5805.0 | 11176.0 | 16839.0 | 0.0 | 3418.0 | 1123.0 | 3760.0 | 0.0 | 3625.0 | 4516.0 | 3438.0 | 0.0 | 4263.0 | 4789.0 | 3014.0 | 0.0 | 3289.0 | 1693.0 | 5294.0 | 3943.0 | 3392.0 | 3201.0 | 0.0 | 3163.0 | 9888.0 | 2513.0 | 5168.0 | 3688.0 | 7964.0 | 0.0 | 0.0 | 0.0 | 0.0 | 0.0 | 0.0 | 0.0 | 0.0 | 0.0 | 0.0 | 0.0 | 0.0 | 1845.0 | 0.0 | 2972.0 | 2177.0 | 2202.0 | 2440.0 | 3118.0 | 1385.0 | 0.0 | 0.0 |
| 181 | 0.0 | 3055.0 | 21619.0 | 22342.0 | 0.0 | 0.0 | 0.0 | 0.0 | 0.0 | 0.0 | 0.0 | 6375.0 | 3027.0 | 0.0 | 0.0 | 2589.0 | 5530.0 | 5342.0 | 0.0 | 1843.0 | 3463.0 | 1828.0 | 4001.0 | 0.0 | 3732.0 | 0.0 | 0.0 | 4274.0 | 5681.0 | 5036.0 | 10574.0 | 15991.0 | 0.0 | 3249.0 | 1136.0 | 3552.0 | 0.0 | 3305.0 | 4060.0 | 3451.0 | 0.0 | 4067.0 | 4542.0 | 3186.0 | 0.0 | 3188.0 | 1884.0 | 5383.0 | 3729.0 | 3207.0 | 3079.0 | 0.0 | 2999.0 | 9431.0 | 2443.0 | 5030.0 | 3408.0 | 7916.0 | 0.0 | 0.0 | 0.0 | 0.0 | 0.0 | 0.0 | 0.0 | 0.0 | 0.0 | 0.0 | 0.0 | 0.0 | 2212.0 | 1255.0 | 3390.0 | 2481.0 | 2551.0 | 2801.0 | 3418.0 | 1646.0 | 0.0 | 0.0 |
| 194 | 0.0 | 2967.0 | 22604.0 | 22082.0 | 0.0 | 0.0 | 0.0 | 0.0 | 0.0 | 0.0 | 0.0 | 5617.0 | 2636.0 | 0.0 | 0.0 | 2252.0 | 4972.0 | 4909.0 | 0.0 | 1756.0 | 3017.0 | 1738.0 | 3475.0 | 0.0 | 3210.0 | 0.0 | 0.0 | 3697.0 | 5290.0 | 4624.0 | 10116.0 | 15042.0 | 0.0 | 2697.0 | 0.0 | 3189.0 | 0.0 | 2919.0 | 3505.0 | 2912.0 | 0.0 | 3644.0 | 3946.0 | 2681.0 | 0.0 | 2640.0 | 1645.0 | 4575.0 | 3211.0 | 2737.0 | 2635.0 | 0.0 | 2535.0 | 8679.0 | 2025.0 | 4249.0 | 2919.0 | 7262.0 | 0.0 | 0.0 | 1799.0 | 0.0 | 0.0 | 0.0 | 0.0 | 0.0 | 0.0 | 0.0 | 0.0 | 0.0 | 2519.0 | 1327.0 | 3304.0 | 2654.0 | 2878.0 | 3162.0 | 2954.0 | 1827.0 | 0.0 | 0.0 |
| 209 | 0.0 | 1786.0 | 22323.0 | 21079.0 | 0.0 | 0.0 | 0.0 | 0.0 | 0.0 | 0.0 | 0.0 | 3702.0 | 1756.0 | 0.0 | 0.0 | 1560.0 | 3193.0 | 3305.0 | 0.0 | 1284.0 | 0.0 | 1284.0 | 2406.0 | 2264.0 | 2376.0 | 0.0 | 0.0 | 2497.0 | 3380.0 | 3348.0 | 6841.0 | 10433.0 | 0.0 | 2031.0 | 0.0 | 2207.0 | 0.0 | 2082.0 | 2512.0 | 2118.0 | 0.0 | 2600.0 | 2655.0 | 1957.0 | 0.0 | 1860.0 | 0.0 | 3405.0 | 2332.0 | 1898.0 | 1849.0 | 0.0 | 1875.0 | 6562.0 | 1439.0 | 3195.0 | 2265.0 | 5609.0 | 0.0 | 0.0 | 1680.0 | 0.0 | 0.0 | 0.0 | 0.0 | 0.0 | 0.0 | 0.0 | 0.0 | 0.0 | 1569.0 | 0.0 | 1959.0 | 1703.0 | 1748.0 | 1860.0 | 2570.0 | 1167.0 | 0.0 | 0.0 |
| 222 | 0.0 | 3314.0 | 22819.0 | 22698.0 | 0.0 | 0.0 | 0.0 | 0.0 | 0.0 | 0.0 | 0.0 | 5335.0 | 2917.0 | 0.0 | 0.0 | 1875.0 | 5241.0 | 5308.0 | 0.0 | 2142.0 | 3232.0 | 1991.0 | 3443.0 | 0.0 | 3241.0 | 0.0 | 0.0 | 3794.0 | 4930.0 | 4526.0 | 10236.0 | 14850.0 | 0.0 | 2704.0 | 1198.0 | 3291.0 | 0.0 | 3011.0 | 3571.0 | 3058.0 | 0.0 | 3776.0 | 3908.0 | 2728.0 | 0.0 | 2674.0 | 1710.0 | 4668.0 | 3422.0 | 2736.0 | 2668.0 | 0.0 | 2587.0 | 9467.0 | 2084.0 | 4427.0 | 3049.0 | 8242.0 | 0.0 | 0.0 | 2577.0 | 0.0 | 0.0 | 0.0 | 0.0 | 0.0 | 0.0 | 0.0 | 0.0 | 0.0 | 2508.0 | 1417.0 | 3242.0 | 2660.0 | 2812.0 | 2923.0 | 3026.0 | 1983.0 | 0.0 | 0.0 |
| 240 | 0.0 | 1860.0 | 22862.0 | 21933.0 | 0.0 | 0.0 | 0.0 | 0.0 | 0.0 | 0.0 | 0.0 | 3305.0 | 1758.0 | 0.0 | 0.0 | 1473.0 | 3263.0 | 3127.0 | 0.0 | 1217.0 | 2160.0 | 1455.0 | 2408.0 | 0.0 | 2262.0 | 0.0 | 0.0 | 2580.0 | 3522.0 | 3134.0 | 7516.0 | 11538.0 | 0.0 | 1955.0 | 0.0 | 2241.0 | 0.0 | 2035.0 | 2420.0 | 2040.0 | 0.0 | 2578.0 | 2740.0 | 1848.0 | 0.0 | 1781.0 | 0.0 | 3329.0 | 2279.0 | 1897.0 | 1860.0 | 0.0 | 1955.0 | 6696.0 | 1409.0 | 3053.0 | 2008.0 | 6046.0 | 0.0 | 0.0 | 0.0 | 0.0 | 0.0 | 0.0 | 0.0 | 0.0 | 0.0 | 0.0 | 0.0 | 0.0 | 1555.0 | 0.0 | 2068.0 | 1590.0 | 1844.0 | 1859.0 | 2484.0 | 0.0 | 0.0 | 0.0 |
| 253 | 0.0 | 2223.0 | 24473.0 | 23878.0 | 0.0 | 0.0 | 0.0 | 0.0 | 0.0 | 0.0 | 0.0 | 4147.0 | 2010.0 | 0.0 | 0.0 | 1692.0 | 3748.0 | 3901.0 | 0.0 | 1478.0 | 2544.0 | 1456.0 | 2771.0 | 0.0 | 2650.0 | 0.0 | 0.0 | 3082.0 | 4343.0 | 3759.0 | 8750.0 | 13472.0 | 0.0 | 2313.0 | 0.0 | 2598.0 | 0.0 | 2454.0 | 2850.0 | 2456.0 | 0.0 | 3084.0 | 3213.0 | 2245.0 | 0.0 | 2161.0 | 1272.0 | 3892.0 | 2731.0 | 2194.0 | 2215.0 | 0.0 | 2197.0 | 7491.0 | 1596.0 | 3683.0 | 2417.0 | 6649.0 | 0.0 | 0.0 | 0.0 | 0.0 | 0.0 | 0.0 | 0.0 | 0.0 | 0.0 | 0.0 | 0.0 | 0.0 | 1976.0 | 0.0 | 2627.0 | 2028.0 | 2301.0 | 2470.0 | 3298.0 | 1471.0 | 0.0 | 0.0 |
| 274 | 0.0 | 3032.0 | 21557.0 | 21899.0 | 0.0 | 0.0 | 0.0 | 0.0 | 0.0 | 0.0 | 0.0 | 4314.0 | 2542.0 | 0.0 | 0.0 | 1722.0 | 4628.0 | 4726.0 | 0.0 | 1797.0 | 2633.0 | 2074.0 | 2946.0 | 0.0 | 2840.0 | 0.0 | 0.0 | 3347.0 | 4609.0 | 4079.0 | 9941.0 | 14163.0 | 0.0 | 2599.0 | 0.0 | 2752.0 | 0.0 | 2560.0 | 3039.0 | 2641.0 | 0.0 | 3543.0 | 3685.0 | 2342.0 | 0.0 | 2257.0 | 1475.0 | 4152.0 | 2959.0 | 2372.0 | 2296.0 | 0.0 | 2402.0 | 8406.0 | 1766.0 | 3888.0 | 2702.0 | 7831.0 | 0.0 | 0.0 | 0.0 | 0.0 | 0.0 | 0.0 | 0.0 | 0.0 | 0.0 | 0.0 | 0.0 | 0.0 | 2786.0 | 1451.0 | 3475.0 | 2937.0 | 3085.0 | 3293.0 | 3274.0 | 2074.0 | 0.0 | 0.0 |
| 286 | 0.0 | 2849.0 | 27095.0 | 25999.0 | 1500.0 | 1291.0 | 1523.0 | 0.0 | 0.0 | 0.0 | 0.0 | 4091.0 | 2825.0 | 0.0 | 0.0 | 1888.0 | 4340.0 | 4428.0 | 0.0 | 1992.0 | 2936.0 | 2197.0 | 3005.0 | 0.0 | 2876.0 | 0.0 | 0.0 | 3298.0 | 4497.0 | 3984.0 | 9651.0 | 14032.0 | 1373.0 | 2847.0 | 1207.0 | 2882.0 | 0.0 | 2688.0 | 3062.0 | 2673.0 | 0.0 | 3604.0 | 3698.0 | 2255.0 | 0.0 | 2227.0 | 1539.0 | 4239.0 | 3242.0 | 2421.0 | 2261.0 | 0.0 | 2492.0 | 8000.0 | 1737.0 | 4028.0 | 3094.0 | 7604.0 | 0.0 | 0.0 | 0.0 | 0.0 | 0.0 | 0.0 | 0.0 | 0.0 | 0.0 | 0.0 | 0.0 | 0.0 | 2104.0 | 1341.0 | 2542.0 | 2305.0 | 2273.0 | 2413.0 | 2729.0 | 1638.0 | 0.0 | 0.0 |
| 321 | 0.0 | 1679.0 | 22087.0 | 22302.0 | 0.0 | 0.0 | 0.0 | 0.0 | 0.0 | 0.0 | 0.0 | 2422.0 | 1492.0 | 0.0 | 0.0 | 1290.0 | 2712.0 | 2682.0 | 0.0 | 0.0 | 1453.0 | 1321.0 | 1600.0 | 0.0 | 1507.0 | 0.0 | 0.0 | 1801.0 | 2662.0 | 2233.0 | 6715.0 | 10029.0 | 0.0 | 1417.0 | 0.0 | 1594.0 | 0.0 | 1406.0 | 1621.0 | 1398.0 | 0.0 | 1954.0 | 2045.0 | 1197.0 | 0.0 | 1218.0 | 0.0 | 2368.0 | 1549.0 | 1263.0 | 1201.0 | 0.0 | 1824.0 | 5762.0 | 0.0 | 2292.0 | 1437.0 | 5545.0 | 0.0 | 0.0 | 0.0 | 0.0 | 0.0 | 0.0 | 0.0 | 0.0 | 0.0 | 0.0 | 0.0 | 0.0 | 1368.0 | 0.0 | 1783.0 | 1434.0 | 1622.0 | 1823.0 | 2449.0 | 0.0 | 0.0 | 0.0 |
| 351 | 0.0 | 1613.0 | 23703.0 | 22398.0 | 0.0 | 0.0 | 0.0 | 0.0 | 0.0 | 0.0 | 0.0 | 2602.0 | 1411.0 | 0.0 | 0.0 | 1230.0 | 2687.0 | 2785.0 | 0.0 | 0.0 | 1433.0 | 1444.0 | 1711.0 | 0.0 | 1596.0 | 0.0 | 0.0 | 1879.0 | 2828.0 | 2339.0 | 7439.0 | 11087.0 | 0.0 | 1428.0 | 0.0 | 1726.0 | 0.0 | 1488.0 | 1636.0 | 1460.0 | 0.0 | 2031.0 | 2120.0 | 1286.0 | 0.0 | 1286.0 | 0.0 | 2393.0 | 1552.0 | 1361.0 | 1240.0 | 0.0 | 1682.0 | 5758.0 | 0.0 | 2300.0 | 1460.0 | 5837.0 | 0.0 | 0.0 | 0.0 | 0.0 | 0.0 | 0.0 | 0.0 | 0.0 | 0.0 | 0.0 | 0.0 | 0.0 | 1843.0 | 0.0 | 2395.0 | 1886.0 | 2129.0 | 2333.0 | 2963.0 | 1242.0 | 0.0 | 0.0 |
| 398 | 0.0 | 1760.0 | 23179.0 | 22164.0 | 0.0 | 0.0 | 0.0 | 0.0 | 0.0 | 0.0 | 0.0 | 2466.0 | 1363.0 | 0.0 | 0.0 | 1188.0 | 2772.0 | 2747.0 | 0.0 | 0.0 | 1355.0 | 1370.0 | 1689.0 | 0.0 | 1525.0 | 0.0 | 0.0 | 1799.0 | 2761.0 | 2214.0 | 8091.0 | 11961.0 | 0.0 | 1274.0 | 0.0 | 1767.0 | 0.0 | 1356.0 | 1639.0 | 1351.0 | 0.0 | 1928.0 | 2073.0 | 1226.0 | 0.0 | 1203.0 | 0.0 | 2283.0 | 1423.0 | 1287.0 | 1232.0 | 0.0 | 1655.0 | 6109.0 | 0.0 | 2122.0 | 1294.0 | 6408.0 | 0.0 | 0.0 | 0.0 | 0.0 | 0.0 | 0.0 | 0.0 | 0.0 | 0.0 | 0.0 | 0.0 | 0.0 | 1828.0 | 0.0 | 2463.0 | 1938.0 | 2154.0 | 2227.0 | 2931.0 | 1274.0 | 0.0 | 0.0 |
| 427 | 2749.0 | 1793.0 | 18547.0 | 17411.0 | 0.0 | 0.0 | 1481.0 | 0.0 | 0.0 | 0.0 | 0.0 | 2135.0 | 1519.0 | 0.0 | 0.0 | 1591.0 | 2344.0 | 2443.0 | 0.0 | 1386.0 | 1782.0 | 1734.0 | 1564.0 | 0.0 | 1506.0 | 0.0 | 0.0 | 1631.0 | 2604.0 | 2192.0 | 6817.0 | 10065.0 | 0.0 | 1735.0 | 0.0 | 1596.0 | 0.0 | 1478.0 | 1650.0 | 1394.0 | 0.0 | 2401.0 | 2310.0 | 1152.0 | 0.0 | 1264.0 | 0.0 | 2366.0 | 2221.0 | 1292.0 | 0.0 | 0.0 | 2010.0 | 5417.0 | 0.0 | 2502.0 | 2023.0 | 5208.0 | 0.0 | 0.0 | 0.0 | 0.0 | 0.0 | 0.0 | 0.0 | 0.0 | 0.0 | 0.0 | 0.0 | 0.0 | 2329.0 | 1389.0 | 2750.0 | 2614.0 | 2479.0 | 2650.0 | 2513.0 | 1835.0 | 0.0 | 0.0 |
| 468 | 0.0 | 9650.0 | 19016.0 | 16431.0 | 0.0 | 0.0 | 0.0 | 0.0 | 0.0 | 0.0 | 0.0 | 1606.0 | 0.0 | 0.0 | 0.0 | 0.0 | 6622.0 | 8071.0 | 0.0 | 0.0 | 15479.0 | 1250.0 | 13422.0 | 0.0 | 14188.0 | 0.0 | 0.0 | 15875.0 | 13880.0 | 15888.0 | 6753.0 | 16842.0 | 0.0 | 13355.0 | 0.0 | 12198.0 | 0.0 | 13825.0 | 14451.0 | 14715.0 | 0.0 | 13159.0 | 13359.0 | 13269.0 | 0.0 | 12768.0 | 10076.0 | 16565.0 | 15135.0 | 14556.0 | 13230.0 | 0.0 | 12048.0 | 15052.0 | 12496.0 | 17685.0 | 13905.0 | 15148.0 | 0.0 | 0.0 | 0.0 | 0.0 | 0.0 | 0.0 | 0.0 | 0.0 | 0.0 | 0.0 | 0.0 | 0.0 | 1554.0 | 0.0 | 1960.0 | 1572.0 | 1834.0 | 1896.0 | 2085.0 | 0.0 | 0.0 | 0.0 |
| 489 | 0.0 | 7198.0 | 18874.0 | 16456.0 | 0.0 | 0.0 | 0.0 | 0.0 | 0.0 | 0.0 | 0.0 | 1323.0 | 0.0 | 0.0 | 0.0 | 0.0 | 4412.0 | 4787.0 | 0.0 | 0.0 | 9021.0 | 0.0 | 7786.0 | 0.0 | 8495.0 | 0.0 | 0.0 | 9423.0 | 8293.0 | 9560.0 | 6102.0 | 12007.0 | 0.0 | 7376.0 | 0.0 | 7077.0 | 0.0 | 7864.0 | 8407.0 | 8392.0 | 0.0 | 7414.0 | 7704.0 | 7658.0 | 0.0 | 6644.0 | 4928.0 | 10653.0 | 8971.0 | 8335.0 | 7293.0 | 0.0 | 6265.0 | 9633.0 | 6592.0 | 10885.0 | 7556.0 | 9445.0 | 0.0 | 0.0 | 0.0 | 0.0 | 0.0 | 0.0 | 0.0 | 0.0 | 0.0 | 0.0 | 0.0 | 0.0 | 1464.0 | 0.0 | 1957.0 | 1536.0 | 1659.0 | 1758.0 | 1826.0 | 0.0 | 0.0 | 0.0 |
| 503 | 0.0 | 8097.0 | 21623.0 | 18926.0 | 0.0 | 0.0 | 0.0 | 0.0 | 0.0 | 0.0 | 0.0 | 1827.0 | 0.0 | 0.0 | 0.0 | 0.0 | 5792.0 | 6824.0 | 0.0 | 0.0 | 8625.0 | 1302.0 | 7700.0 | 0.0 | 8146.0 | 0.0 | 0.0 | 8822.0 | 8496.0 | 9395.0 | 7779.0 | 13291.0 | 0.0 | 7559.0 | 0.0 | 6941.0 | 0.0 | 7536.0 | 8336.0 | 8201.0 | 0.0 | 7564.0 | 7913.0 | 7633.0 | 0.0 | 6751.0 | 4896.0 | 9736.0 | 8775.0 | 8381.0 | 7396.0 | 0.0 | 6675.0 | 10280.0 | 6741.0 | 10136.0 | 7652.0 | 10429.0 | 0.0 | 0.0 | 0.0 | 0.0 | 0.0 | 0.0 | 0.0 | 0.0 | 0.0 | 0.0 | 0.0 | 0.0 | 2633.0 | 0.0 | 3435.0 | 2973.0 | 3016.0 | 3152.0 | 2545.0 | 2627.0 | 0.0 | 0.0 |
| 526 | 0.0 | 4444.0 | 18047.0 | 15505.0 | 0.0 | 0.0 | 0.0 | 0.0 | 0.0 | 0.0 | 0.0 | 1350.0 | 0.0 | 0.0 | 0.0 | 0.0 | 3228.0 | 3961.0 | 0.0 | 0.0 | 4783.0 | 0.0 | 4271.0 | 0.0 | 4722.0 | 0.0 | 0.0 | 5061.0 | 4715.0 | 5288.0 | 6371.0 | 9453.0 | 0.0 | 3827.0 | 0.0 | 3578.0 | 0.0 | 4169.0 | 4478.0 | 4549.0 | 0.0 | 4104.0 | 4163.0 | 4078.0 | 0.0 | 3326.0 | 2509.0 | 6125.0 | 4887.0 | 4480.0 | 3937.0 | 0.0 | 3499.0 | 6886.0 | 3393.0 | 5748.0 | 4070.0 | 7442.0 | 0.0 | 0.0 | 0.0 | 0.0 | 0.0 | 0.0 | 0.0 | 0.0 | 0.0 | 0.0 | 0.0 | 0.0 | 2927.0 | 0.0 | 3905.0 | 3252.0 | 3042.0 | 3096.0 | 2348.0 | 2363.0 | 0.0 | 0.0 |
| 527 | 0.0 | 4399.0 | 18051.0 | 15618.0 | 0.0 | 0.0 | 0.0 | 0.0 | 0.0 | 0.0 | 0.0 | 1434.0 | 0.0 | 0.0 | 0.0 | 0.0 | 3101.0 | 3549.0 | 0.0 | 0.0 | 5261.0 | 0.0 | 4692.0 | 0.0 | 5043.0 | 0.0 | 0.0 | 5416.0 | 5223.0 | 5773.0 | 6410.0 | 9936.0 | 0.0 | 4209.0 | 0.0 | 3931.0 | 0.0 | 4475.0 | 4941.0 | 4991.0 | 0.0 | 4495.0 | 4604.0 | 4528.0 | 0.0 | 3780.0 | 2688.0 | 6767.0 | 5350.0 | 5056.0 | 4327.0 | 0.0 | 3850.0 | 7259.0 | 3655.0 | 6682.0 | 4364.0 | 7605.0 | 0.0 | 0.0 | 0.0 | 0.0 | 0.0 | 0.0 | 0.0 | 0.0 | 0.0 | 0.0 | 0.0 | 0.0 | 3124.0 | 0.0 | 4083.0 | 3541.0 | 3209.0 | 3259.0 | 2332.0 | 2282.0 | 0.0 | 0.0 |
| 539 | 0.0 | 5304.0 | 20650.0 | 18810.0 | 0.0 | 0.0 | 0.0 | 0.0 | 0.0 | 0.0 | 0.0 | 1853.0 | 0.0 | 0.0 | 0.0 | 0.0 | 4113.0 | 4946.0 | 0.0 | 0.0 | 5036.0 | 1282.0 | 4497.0 | 0.0 | 4753.0 | 0.0 | 0.0 | 5277.0 | 5245.0 | 5504.0 | 8722.0 | 12238.0 | 0.0 | 3971.0 | 0.0 | 3850.0 | 0.0 | 4316.0 | 4730.0 | 4659.0 | 0.0 | 4256.0 | 4509.0 | 4206.0 | 0.0 | 3472.0 | 2734.0 | 6272.0 | 5071.0 | 4786.0 | 4029.0 | 0.0 | 3744.0 | 8138.0 | 3509.0 | 5990.0 | 4062.0 | 8752.0 | 0.0 | 0.0 | 0.0 | 0.0 | 0.0 | 0.0 | 0.0 | 0.0 | 0.0 | 0.0 | 0.0 | 0.0 | 10580.0 | 0.0 | 12986.0 | 10917.0 | 9896.0 | 9197.0 | 2335.0 | 2601.0 | 0.0 | 0.0 |
| 574 | 0.0 | 3666.0 | 18977.0 | 16895.0 | 0.0 | 0.0 | 0.0 | 0.0 | 0.0 | 0.0 | 0.0 | 1814.0 | 0.0 | 0.0 | 0.0 | 0.0 | 3243.0 | 3739.0 | 0.0 | 0.0 | 3394.0 | 1223.0 | 2885.0 | 0.0 | 3253.0 | 0.0 | 0.0 | 3509.0 | 3645.0 | 3669.0 | 8555.0 | 10610.0 | 0.0 | 2625.0 | 0.0 | 2618.0 | 0.0 | 2770.0 | 3138.0 | 3011.0 | 0.0 | 2893.0 | 3166.0 | 2680.0 | 0.0 | 2389.0 | 1733.0 | 4228.0 | 3245.0 | 3077.0 | 2636.0 | 0.0 | 2657.0 | 6893.0 | 2264.0 | 4011.0 | 2685.0 | 7582.0 | 0.0 | 0.0 | 0.0 | 0.0 | 0.0 | 0.0 | 0.0 | 0.0 | 0.0 | 0.0 | 0.0 | 0.0 | 17600.0 | 0.0 | 20579.0 | 17978.0 | 15850.0 | 15509.0 | 2329.0 | 1863.0 | 0.0 | 0.0 |
| 588 | 0.0 | 3172.0 | 18386.0 | 16137.0 | 0.0 | 0.0 | 0.0 | 0.0 | 0.0 | 0.0 | 0.0 | 1744.0 | 0.0 | 0.0 | 0.0 | 0.0 | 2901.0 | 3336.0 | 0.0 | 0.0 | 2791.0 | 0.0 | 2402.0 | 0.0 | 2760.0 | 0.0 | 0.0 | 3057.0 | 3234.0 | 3084.0 | 8037.0 | 10561.0 | 0.0 | 2034.0 | 0.0 | 2165.0 | 0.0 | 2260.0 | 2678.0 | 2615.0 | 0.0 | 2422.0 | 2481.0 | 2330.0 | 0.0 | 1869.0 | 1482.0 | 3627.0 | 2705.0 | 2604.0 | 2308.0 | 0.0 | 2128.0 | 6427.0 | 1856.0 | 3413.0 | 2129.0 | 7257.0 | 0.0 | 0.0 | 0.0 | 0.0 | 0.0 | 0.0 | 0.0 | 0.0 | 0.0 | 0.0 | 0.0 | 0.0 | 17700.0 | 0.0 | 20603.0 | 18014.0 | 16760.0 | 15705.0 | 2243.0 | 1456.0 | 0.0 | 0.0 |
| 602 | 0.0 | 3773.0 | 22474.0 | 19692.0 | 0.0 | 0.0 | 0.0 | 0.0 | 0.0 | 0.0 | 0.0 | 2225.0 | 1506.0 | 0.0 | 0.0 | 0.0 | 3510.0 | 3841.0 | 0.0 | 0.0 | 3429.0 | 1597.0 | 2914.0 | 0.0 | 3374.0 | 0.0 | 0.0 | 3710.0 | 3954.0 | 3948.0 | 10393.0 | 12826.0 | 0.0 | 2514.0 | 0.0 | 2579.0 | 0.0 | 2835.0 | 3440.0 | 3200.0 | 0.0 | 2721.0 | 2897.0 | 2906.0 | 0.0 | 2248.0 | 1748.0 | 4635.0 | 3289.0 | 3198.0 | 2845.0 | 0.0 | 2428.0 | 8075.0 | 2286.0 | 4144.0 | 2631.0 | 8983.0 | 0.0 | 0.0 | 0.0 | 0.0 | 0.0 | 0.0 | 0.0 | 0.0 | 0.0 | 0.0 | 0.0 | 0.0 | 20476.0 | 0.0 | 23681.0 | 20469.0 | 18673.0 | 18128.0 | 2652.0 | 1466.0 | 0.0 | 0.0 |
| 617 | 0.0 | 2412.0 | 18503.0 | 15684.0 | 0.0 | 0.0 | 0.0 | 0.0 | 0.0 | 0.0 | 0.0 | 1626.0 | 0.0 | 0.0 | 0.0 | 0.0 | 2416.0 | 2723.0 | 0.0 | 0.0 | 2191.0 | 0.0 | 2001.0 | 0.0 | 2135.0 | 0.0 | 0.0 | 2363.0 | 2665.0 | 2527.0 | 7809.0 | 9501.0 | 0.0 | 1631.0 | 0.0 | 1688.0 | 0.0 | 1819.0 | 2089.0 | 2033.0 | 0.0 | 1977.0 | 2136.0 | 1805.0 | 0.0 | 1439.0 | 0.0 | 2900.0 | 1967.0 | 2062.0 | 1801.0 | 0.0 | 2120.0 | 5685.0 | 1457.0 | 2571.0 | 1603.0 | 6434.0 | 0.0 | 0.0 | 0.0 | 0.0 | 0.0 | 0.0 | 0.0 | 0.0 | 0.0 | 0.0 | 0.0 | 0.0 | 21311.0 | 0.0 | 22597.0 | 20910.0 | 17134.0 | 18245.0 | 2790.0 | 0.0 | 0.0 | 0.0 |
| 658 | 0.0 | 1336.0 | 12976.0 | 10844.0 | 0.0 | 0.0 | 0.0 | 0.0 | 0.0 | 0.0 | 0.0 | 0.0 | 0.0 | 0.0 | 0.0 | 0.0 | 1486.0 | 1543.0 | 0.0 | 0.0 | 1276.0 | 0.0 | 0.0 | 0.0 | 1217.0 | 0.0 | 0.0 | 1371.0 | 1774.0 | 1503.0 | 6901.0 | 8002.0 | 0.0 | 0.0 | 0.0 | 0.0 | 0.0 | 0.0 | 1247.0 | 1198.0 | 0.0 | 0.0 | 1276.0 | 0.0 | 0.0 | 0.0 | 0.0 | 1930.0 | 1251.0 | 0.0 | 0.0 | 0.0 | 1254.0 | 5533.0 | 0.0 | 1554.0 | 0.0 | 5758.0 | 0.0 | 0.0 | 0.0 | 0.0 | 0.0 | 0.0 | 0.0 | 0.0 | 0.0 | 0.0 | 0.0 | 0.0 | 18058.0 | 0.0 | 19538.0 | 17875.0 | 14993.0 | 14867.0 | 2277.0 | 0.0 | 0.0 | 0.0 |
| 672 | 0.0 | 2025.0 | 18039.0 | 15155.0 | 0.0 | 0.0 | 0.0 | 0.0 | 0.0 | 0.0 | 0.0 | 1649.0 | 0.0 | 0.0 | 0.0 | 0.0 | 2317.0 | 2028.0 | 0.0 | 0.0 | 1782.0 | 0.0 | 1655.0 | 0.0 | 1738.0 | 0.0 | 0.0 | 1974.0 | 2508.0 | 2158.0 | 10612.0 | 12238.0 | 0.0 | 1417.0 | 0.0 | 1491.0 | 0.0 | 1483.0 | 1794.0 | 1709.0 | 0.0 | 1680.0 | 1846.0 | 1463.0 | 0.0 | 0.0 | 0.0 | 2703.0 | 1704.0 | 1671.0 | 1571.0 | 0.0 | 1777.0 | 8577.0 | 0.0 | 2205.0 | 1407.0 | 8612.0 | 0.0 | 0.0 | 0.0 | 0.0 | 0.0 | 0.0 | 0.0 | 0.0 | 0.0 | 0.0 | 0.0 | 0.0 | 20899.0 | 0.0 | 22379.0 | 21010.0 | 17574.0 | 17550.0 | 2841.0 | 1395.0 | 0.0 | 0.0 |
| 686 | 0.0 | 1764.0 | 15131.0 | 13647.0 | 0.0 | 0.0 | 0.0 | 0.0 | 0.0 | 0.0 | 0.0 | 1368.0 | 0.0 | 0.0 | 0.0 | 0.0 | 2168.0 | 2233.0 | 0.0 | 0.0 | 0.0 | 0.0 | 0.0 | 0.0 | 0.0 | 0.0 | 0.0 | 0.0 | 2195.0 | 1460.0 | 9361.0 | 10383.0 | 0.0 | 0.0 | 0.0 | 0.0 | 0.0 | 0.0 | 0.0 | 0.0 | 0.0 | 0.0 | 1356.0 | 0.0 | 0.0 | 0.0 | 0.0 | 1799.0 | 0.0 | 0.0 | 0.0 | 0.0 | 1482.0 | 7315.0 | 0.0 | 1305.0 | 0.0 | 7903.0 | 0.0 | 0.0 | 2077.0 | 0.0 | 0.0 | 0.0 | 0.0 | 0.0 | 0.0 | 0.0 | 0.0 | 0.0 | 21001.0 | 0.0 | 22423.0 | 20956.0 | 17787.0 | 17857.0 | 3792.0 | 1770.0 | 0.0 | 0.0 |
| 700 | 0.0 | 1414.0 | 13248.0 | 10813.0 | 0.0 | 0.0 | 0.0 | 0.0 | 0.0 | 0.0 | 0.0 | 1276.0 | 0.0 | 0.0 | 0.0 | 0.0 | 1701.0 | 1542.0 | 0.0 | 0.0 | 1313.0 | 0.0 | 0.0 | 0.0 | 0.0 | 0.0 | 0.0 | 1374.0 | 1792.0 | 1494.0 | 8464.0 | 9501.0 | 0.0 | 0.0 | 0.0 | 0.0 | 0.0 | 0.0 | 0.0 | 0.0 | 0.0 | 0.0 | 1383.0 | 0.0 | 0.0 | 0.0 | 0.0 | 1814.0 | 0.0 | 0.0 | 0.0 | 0.0 | 1341.0 | 6502.0 | 0.0 | 1402.0 | 0.0 | 6677.0 | 0.0 | 0.0 | 0.0 | 0.0 | 0.0 | 0.0 | 0.0 | 0.0 | 0.0 | 0.0 | 0.0 | 0.0 | 16288.0 | 0.0 | 17163.0 | 15934.0 | 13188.0 | 13880.0 | 1990.0 | 0.0 | 0.0 | 0.0 |
| 714 | 0.0 | 0.0 | 12933.0 | 11013.0 | 0.0 | 0.0 | 0.0 | 0.0 | 0.0 | 0.0 | 0.0 | 0.0 | 0.0 | 0.0 | 0.0 | 0.0 | 1900.0 | 1977.0 | 0.0 | 0.0 | 0.0 | 0.0 | 0.0 | 0.0 | 0.0 | 0.0 | 0.0 | 0.0 | 0.0 | 0.0 | 8330.0 | 9387.0 | 0.0 | 0.0 | 0.0 | 0.0 | 0.0 | 0.0 | 0.0 | 0.0 | 0.0 | 0.0 | 0.0 | 0.0 | 0.0 | 0.0 | 0.0 | 0.0 | 0.0 | 0.0 | 0.0 | 0.0 | 0.0 | 6531.0 | 0.0 | 0.0 | 0.0 | 7172.0 | 0.0 | 0.0 | 0.0 | 0.0 | 0.0 | 0.0 | 0.0 | 0.0 | 0.0 | 0.0 | 0.0 | 0.0 | 15581.0 | 0.0 | 17348.0 | 15850.0 | 13063.0 | 13238.0 | 2261.0 | 0.0 | 0.0 | 0.0 |
| 728 | 0.0 | 1323.0 | 13964.0 | 11985.0 | 0.0 | 0.0 | 0.0 | 0.0 | 0.0 | 0.0 | 0.0 | 1313.0 | 0.0 | 0.0 | 0.0 | 0.0 | 1793.0 | 1890.0 | 0.0 | 0.0 | 1177.0 | 0.0 | 0.0 | 0.0 | 0.0 | 0.0 | 0.0 | 1324.0 | 1682.0 | 1419.0 | 8401.0 | 9767.0 | 0.0 | 0.0 | 0.0 | 0.0 | 0.0 | 0.0 | 1203.0 | 0.0 | 0.0 | 0.0 | 1305.0 | 0.0 | 0.0 | 0.0 | 0.0 | 1820.0 | 0.0 | 0.0 | 0.0 | 0.0 | 1317.0 | 6530.0 | 0.0 | 1427.0 | 0.0 | 7545.0 | 1820.0 | 0.0 | 5412.0 | 5486.0 | 0.0 | 0.0 | 0.0 | 0.0 | 0.0 | 0.0 | 0.0 | 0.0 | 16907.0 | 0.0 | 18315.0 | 17046.0 | 14119.0 | 14386.0 | 2106.0 | 0.0 | 0.0 | 0.0 |
| 742 | 0.0 | 1227.0 | 13860.0 | 11680.0 | 0.0 | 0.0 | 0.0 | 0.0 | 0.0 | 0.0 | 0.0 | 1273.0 | 0.0 | 0.0 | 0.0 | 0.0 | 1660.0 | 1743.0 | 0.0 | 0.0 | 0.0 | 0.0 | 0.0 | 0.0 | 0.0 | 0.0 | 0.0 | 1186.0 | 1618.0 | 1344.0 | 8537.0 | 9750.0 | 0.0 | 0.0 | 0.0 | 0.0 | 0.0 | 0.0 | 1117.0 | 0.0 | 0.0 | 0.0 | 1229.0 | 0.0 | 0.0 | 0.0 | 0.0 | 1789.0 | 0.0 | 0.0 | 0.0 | 0.0 | 1226.0 | 6381.0 | 0.0 | 1384.0 | 0.0 | 7167.0 | 0.0 | 0.0 | 3594.0 | 2931.0 | 0.0 | 0.0 | 0.0 | 0.0 | 0.0 | 0.0 | 0.0 | 0.0 | 17266.0 | 0.0 | 18852.0 | 16997.0 | 14499.0 | 14479.0 | 1876.0 | 0.0 | 0.0 | 0.0 |
| 770 | 0.0 | 1211.0 | 15539.0 | 12808.0 | 0.0 | 0.0 | 0.0 | 0.0 | 0.0 | 0.0 | 0.0 | 1484.0 | 0.0 | 0.0 | 0.0 | 0.0 | 1648.0 | 1755.0 | 0.0 | 0.0 | 0.0 | 0.0 | 0.0 | 0.0 | 0.0 | 0.0 | 0.0 | 1274.0 | 1771.0 | 1445.0 | 8832.0 | 10215.0 | 0.0 | 0.0 | 0.0 | 0.0 | 0.0 | 0.0 | 1186.0 | 0.0 | 0.0 | 0.0 | 1344.0 | 0.0 | 0.0 | 0.0 | 0.0 | 1886.0 | 0.0 | 0.0 | 0.0 | 0.0 | 0.0 | 6447.0 | 0.0 | 1447.0 | 0.0 | 7168.0 | 0.0 | 0.0 | 1964.0 | 1744.0 | 0.0 | 0.0 | 0.0 | 0.0 | 0.0 | 0.0 | 0.0 | 0.0 | 17485.0 | 0.0 | 19337.0 | 17838.0 | 14927.0 | 14734.0 | 2255.0 | 0.0 | 0.0 | 0.0 |
### Chart: Case 2: All Antibodies
| Category | A1 | A11 | A23 | A24 | A25 | A26 | A29 | A3 | A30 | A31 | A32 | A33 | A34 | A36 | A43 | A66 | A74 | A80 | B13 | B18 | B27 | B35 | B37 | B38 | B39 | B41 | B42 | B44 | B45 | B46 | B47 | B48 | B50 | B51 | B52 | B54 | B55 | B56 | B57 | B59 | B60 | B61 | B62 | B63 | B64 | B65 | B67 | B7 | B71 | B72 | B73 | B75 | B76 | B77 | B78 | B8 | B81 | B82 | Cw1 | Cw10 | Cw12 | Cw14 | Cw15 | Cw16 | Cw8 | Cw9 | DP1 | DP10 | DP14 | DP15 | DP17 | DP18 | DP19 | DP2 | DP20 | DP23 | DP28 | DP3 | DP401 | DP402 | DP6 | DP9 | DPA1*01 | DPA1*03 | DQ4 | DQ5 | DQ6 | DQA1*04 | DR1 | DR10 | DR103 | DR12 | DR15 | DR16 | DR4 | DR51 | DR53 | DR7 | DR9 | DRB1*14:01 |
|---|---|---|---|---|---|---|---|---|---|---|---|---|---|---|---|---|---|---|---|---|---|---|---|---|---|---|---|---|---|---|---|---|---|---|---|---|---|---|---|---|---|---|---|---|---|---|---|---|---|---|---|---|---|---|---|---|---|---|---|---|---|---|---|---|---|---|---|---|---|---|---|---|---|---|---|---|---|---|---|---|---|---|---|---|---|---|---|---|---|---|---|---|---|---|---|---|---|---|---|---|
| -19 | 6538.0 | 12523.0 | 10781.0 | 13407.0 | 9184.0 | 10046.0 | 12260.0 | 15248.0 | 13861.0 | 13320.0 | 10296.0 | 13054.0 | 8767.0 | 11351.0 | 8706.0 | 7328.0 | 13481.0 | 8816.0 | 11584.0 | 15116.0 | 13722.0 | 13524.0 | 7021.0 | 5388.0 | 15365.0 | 11729.0 | 14377.0 | 10156.0 | 10248.0 | 12470.0 | 11251.0 | 11634.0 | 13737.0 | 5046.0 | 3345.0 | 15077.0 | 13222.0 | 15300.0 | 13963.0 | 6553.0 | 13912.0 | 14788.0 | 15542.0 | 13065.0 | 14050.0 | 11789.0 | 13502.0 | 12670.0 | 12650.0 | 14948.0 | 13526.0 | 13928.0 | 8256.0 | 11833.0 | 11701.0 | 14822.0 | 13510.0 | 11049.0 | 6519.0 | 11284.0 | 7228.0 | 3577.0 | 2629.0 | 5253.0 | 7965.0 | 13759.0 | 0.0 | 3646.0 | 3725.0 | 7874.0 | 3814.0 | 18024.0 | 18851.0 | 21484.0 | 17198.0 | 18838.0 | 18550.0 | 14330.0 | 21029.0 | 20277.0 | 10404.0 | 3714.0 | 15852.0 | 16058.0 | 6992.0 | 19217.0 | 19116.0 | 0.0 | 4147.0 | 7409.0 | 3740.0 | 2009.0 | 21902.0 | 21821.0 | 18142.0 | 7758.0 | 19270.0 | 7395.0 | 5517.0 | 2596.0 |
| 7 | 15358.0 | 18221.0 | 18122.0 | 15483.0 | 6587.0 | 8140.0 | 19353.0 | 18211.0 | 17263.0 | 17050.0 | 7797.0 | 15136.0 | 6127.0 | 18956.0 | 8006.0 | 4803.0 | 18013.0 | 17528.0 | 11752.0 | 12264.0 | 20952.0 | 10410.0 | 5461.0 | 3118.0 | 13568.0 | 9498.0 | 16723.0 | 11899.0 | 14237.0 | 10370.0 | 12221.0 | 10061.0 | 10959.0 | 3284.0 | 2013.0 | 13272.0 | 12669.0 | 14644.0 | 13365.0 | 4247.0 | 16903.0 | 17384.0 | 19498.0 | 12793.0 | 11412.0 | 8944.0 | 20221.0 | 20172.0 | 10641.0 | 12331.0 | 13051.0 | 15440.0 | 17739.0 | 12684.0 | 9383.0 | 18790.0 | 14873.0 | 11958.0 | 4732.0 | 9845.0 | 4683.0 | 0.0 | 0.0 | 3411.0 | 5280.0 | 12551.0 | 0.0 | 0.0 | 0.0 | 3426.0 | 0.0 | 10861.0 | 10828.0 | 16479.0 | 10105.0 | 13046.0 | 12228.0 | 7698.0 | 14898.0 | 13974.0 | 0.0 | 0.0 | 8518.0 | 8099.0 | 3604.0 | 15773.0 | 16538.0 | 0.0 | 1609.0 | 2599.0 | 1496.0 | 0.0 | 20670.0 | 19909.0 | 9940.0 | 3519.0 | 12176.0 | 1676.0 | 2376.0 | 0.0 |
| 24 | 11528.0 | 18283.0 | 16410.0 | 15823.0 | 6881.0 | 8767.0 | 18581.0 | 19560.0 | 18564.0 | 17806.0 | 8453.0 | 16302.0 | 6679.0 | 17504.0 | 9186.0 | 5421.0 | 18661.0 | 15082.0 | 12696.0 | 13050.0 | 20956.0 | 10896.0 | 5700.0 | 3732.0 | 14361.0 | 9695.0 | 17630.0 | 12451.0 | 13875.0 | 11210.0 | 12955.0 | 10501.0 | 10767.0 | 3466.0 | 2232.0 | 13454.0 | 13129.0 | 15057.0 | 14161.0 | 3829.0 | 16346.0 | 17848.0 | 20264.0 | 13388.0 | 11619.0 | 9278.0 | 20451.0 | 20721.0 | 10771.0 | 12624.0 | 13111.0 | 16017.0 | 14792.0 | 13710.0 | 9722.0 | 19642.0 | 15912.0 | 12274.0 | 4544.0 | 10266.0 | 4584.0 | 0.0 | 0.0 | 3183.0 | 5215.0 | 12863.0 | 0.0 | 0.0 | 0.0 | 3013.0 | 0.0 | 9308.0 | 9519.0 | 14913.0 | 8458.0 | 11855.0 | 10577.0 | 6875.0 | 13673.0 | 12718.0 | 4711.0 | 0.0 | 7436.0 | 6849.0 | 3174.0 | 14986.0 | 15793.0 | 0.0 | 1221.0 | 2164.0 | 0.0 | 1031.0 | 19233.0 | 18192.0 | 8882.0 | 2998.0 | 11737.0 | 1630.0 | 1876.0 | 0.0 |
| 45 | 6837.0 | 18064.0 | 13511.0 | 13817.0 | 6431.0 | 8704.0 | 16943.0 | 19025.0 | 17903.0 | 17835.0 | 7674.0 | 16400.0 | 6150.0 | 14097.0 | 8829.0 | 5471.0 | 18489.0 | 11812.0 | 13377.0 | 12351.0 | 21384.0 | 10470.0 | 6685.0 | 3672.0 | 13160.0 | 9544.0 | 15809.0 | 12993.0 | 13140.0 | 9974.0 | 14106.0 | 11452.0 | 10589.0 | 3475.0 | 2261.0 | 12281.0 | 12212.0 | 13520.0 | 12977.0 | 3303.0 | 15510.0 | 17158.0 | 18764.0 | 12018.0 | 11049.0 | 8868.0 | 19845.0 | 20684.0 | 10051.0 | 11591.0 | 11684.0 | 15131.0 | 10063.0 | 12752.0 | 8711.0 | 17759.0 | 15839.0 | 12502.0 | 4311.0 | 8890.0 | 4197.0 | 0.0 | 0.0 | 2998.0 | 4441.0 | 11583.0 | 0.0 | 0.0 | 0.0 | 2045.0 | 0.0 | 8220.0 | 8294.0 | 12690.0 | 8476.0 | 11557.0 | 9347.0 | 6248.0 | 12083.0 | 11626.0 | 0.0 | 0.0 | 7513.0 | 5836.0 | 2505.0 | 12922.0 | 13917.0 | 0.0 | 1257.0 | 2064.0 | 0.0 | 0.0 | 17244.0 | 16475.0 | 7392.0 | 2390.0 | 10033.0 | 1109.0 | 1609.0 | 0.0 |
| 56 | 15193.0 | 19160.0 | 19555.0 | 15010.0 | 4729.0 | 6398.0 | 22564.0 | 17520.0 | 18076.0 | 16308.0 | 5523.0 | 15235.0 | 4388.0 | 21788.0 | 7420.0 | 3935.0 | 18330.0 | 20317.0 | 11354.0 | 9289.0 | 22723.0 | 7532.0 | 5312.0 | 2600.0 | 10128.0 | 6882.0 | 13377.0 | 12203.0 | 14317.0 | 7785.0 | 12225.0 | 8585.0 | 7964.0 | 2487.0 | 1556.0 | 9660.0 | 9545.0 | 10952.0 | 10229.0 | 2235.0 | 12429.0 | 14610.0 | 16410.0 | 9554.0 | 8344.0 | 6453.0 | 17147.0 | 18744.0 | 7491.0 | 8738.0 | 8778.0 | 12306.0 | 19301.0 | 10105.0 | 6602.0 | 14935.0 | 13199.0 | 11206.0 | 3055.0 | 6793.0 | 2862.0 | 0.0 | 0.0 | 2021.0 | 3126.0 | 8994.0 | 0.0 | 0.0 | 0.0 | 0.0 | 0.0 | 6768.0 | 6742.0 | 10911.0 | 7362.0 | 9717.0 | 8010.0 | 0.0 | 9938.0 | 9781.0 | 0.0 | 0.0 | 7245.0 | 4773.0 | 1548.0 | 9456.0 | 10110.0 | 0.0 | 0.0 | 1377.0 | 0.0 | 0.0 | 13359.0 | 12825.0 | 5325.0 | 1511.0 | 7007.0 | 0.0 | 0.0 | 0.0 |
| 66 | 17111.0 | 15887.0 | 20111.0 | 13512.0 | 3009.0 | 3784.0 | 22101.0 | 13602.0 | 14425.0 | 13089.0 | 3503.0 | 11736.0 | 2662.0 | 22609.0 | 4096.0 | 2137.0 | 14941.0 | 21609.0 | 8304.0 | 6270.0 | 19295.0 | 5382.0 | 3137.0 | 1363.0 | 7007.0 | 4662.0 | 9682.0 | 9721.0 | 11937.0 | 4436.0 | 8771.0 | 5367.0 | 5182.0 | 1311.0 | 0.0 | 6716.0 | 6692.0 | 7325.0 | 6860.0 | 1235.0 | 8812.0 | 10330.0 | 11748.0 | 6220.0 | 5382.0 | 3970.0 | 13473.0 | 13949.0 | 5130.0 | 5898.0 | 5901.0 | 8794.0 | 21674.0 | 7013.0 | 4288.0 | 11224.0 | 9399.0 | 8611.0 | 0.0 | 4522.0 | 0.0 | 0.0 | 0.0 | 0.0 | 0.0 | 6225.0 | 0.0 | 0.0 | 0.0 | 0.0 | 0.0 | 4764.0 | 4703.0 | 8214.0 | 4829.0 | 7089.0 | 5567.0 | 0.0 | 7234.0 | 7200.0 | 0.0 | 0.0 | 4807.0 | 3221.0 | 1099.0 | 7411.0 | 8377.0 | 0.0 | 0.0 | 0.0 | 0.0 | 0.0 | 10605.0 | 9987.0 | 3894.0 | 0.0 | 5680.0 | 0.0 | 0.0 | 0.0 |
| 80 | 10062.0 | 20234.0 | 20269.0 | 16327.0 | 5736.0 | 7089.0 | 23393.0 | 18328.0 | 18855.0 | 17585.0 | 6250.0 | 16040.0 | 5351.0 | 20396.0 | 7187.0 | 4336.0 | 18629.0 | 18421.0 | 11980.0 | 10011.0 | 23284.0 | 8970.0 | 5553.0 | 2824.0 | 11180.0 | 7988.0 | 14056.0 | 13355.0 | 15784.0 | 7596.0 | 12984.0 | 8790.0 | 9005.0 | 2709.0 | 1740.0 | 10355.0 | 10290.0 | 11814.0 | 9902.0 | 2496.0 | 12136.0 | 14370.0 | 16181.0 | 9236.0 | 9113.0 | 7162.0 | 17617.0 | 18776.0 | 8585.0 | 10044.0 | 9751.0 | 12667.0 | 17188.0 | 10020.0 | 7233.0 | 15568.0 | 13572.0 | 12407.0 | 3414.0 | 7533.0 | 3532.0 | 1661.0 | 0.0 | 2455.0 | 3794.0 | 9825.0 | 0.0 | 0.0 | 0.0 | 0.0 | 0.0 | 5331.0 | 5444.0 | 8652.0 | 5520.0 | 7673.0 | 6051.0 | 0.0 | 7788.0 | 7385.0 | 0.0 | 0.0 | 5520.0 | 3875.0 | 1109.0 | 7668.0 | 8664.0 | 0.0 | 0.0 | 0.0 | 0.0 | 0.0 | 10579.0 | 9604.0 | 4156.0 | 0.0 | 5910.0 | 0.0 | 0.0 | 0.0 |
| 87 | 13699.0 | 21188.0 | 22037.0 | 17314.0 | 6476.0 | 7639.0 | 24314.0 | 19207.0 | 19631.0 | 18429.0 | 7073.0 | 16759.0 | 5838.0 | 22941.0 | 7651.0 | 4890.0 | 20076.0 | 20180.0 | 12737.0 | 10884.0 | 23513.0 | 9847.0 | 5843.0 | 2983.0 | 12076.0 | 8910.0 | 14732.0 | 14087.0 | 16410.0 | 8170.0 | 13378.0 | 9477.0 | 10105.0 | 2934.0 | 1827.0 | 11158.0 | 10686.0 | 12510.0 | 10525.0 | 2662.0 | 12957.0 | 15341.0 | 16876.0 | 10375.0 | 9867.0 | 7767.0 | 18383.0 | 19553.0 | 9366.0 | 11141.0 | 10557.0 | 13473.0 | 20605.0 | 10640.0 | 8124.0 | 16441.0 | 14228.0 | 12972.0 | 3640.0 | 8078.0 | 3695.0 | 0.0 | 0.0 | 2653.0 | 4170.0 | 10716.0 | 0.0 | 0.0 | 0.0 | 0.0 | 0.0 | 5233.0 | 5456.0 | 8739.0 | 5644.0 | 7662.0 | 6076.0 | 0.0 | 7839.0 | 7468.0 | 0.0 | 0.0 | 5507.0 | 3765.0 | 1173.0 | 7422.0 | 8540.0 | 0.0 | 0.0 | 0.0 | 0.0 | 0.0 | 10044.0 | 9300.0 | 4057.0 | 0.0 | 5751.0 | 0.0 | 0.0 | 0.0 |
| 94 | 18160.0 | 13376.0 | 20389.0 | 12396.0 | 2843.0 | 3778.0 | 20843.0 | 11880.0 | 12861.0 | 11964.0 | 2956.0 | 11306.0 | 2400.0 | 21984.0 | 4580.0 | 2421.0 | 13038.0 | 21909.0 | 7429.0 | 5587.0 | 16512.0 | 4633.0 | 3177.0 | 1438.0 | 5653.0 | 4086.0 | 7908.0 | 8520.0 | 10706.0 | 3623.0 | 7912.0 | 5472.0 | 4526.0 | 1378.0 | 0.0 | 5481.0 | 5282.0 | 5995.0 | 5589.0 | 0.0 | 7820.0 | 8778.0 | 9556.0 | 4999.0 | 4422.0 | 3391.0 | 11018.0 | 12231.0 | 4144.0 | 4947.0 | 4788.0 | 7232.0 | 21894.0 | 5380.0 | 3550.0 | 9422.0 | 8444.0 | 7688.0 | 0.0 | 3786.0 | 0.0 | 0.0 | 0.0 | 0.0 | 0.0 | 5094.0 | 3002.0 | 0.0 | 0.0 | 0.0 | 0.0 | 4479.0 | 4773.0 | 7128.0 | 4988.0 | 6526.0 | 5078.0 | 0.0 | 6657.0 | 6419.0 | 0.0 | 0.0 | 4665.0 | 3298.0 | 0.0 | 6327.0 | 7274.0 | 0.0 | 0.0 | 0.0 | 0.0 | 0.0 | 8778.0 | 8380.0 | 3753.0 | 0.0 | 5204.0 | 0.0 | 0.0 | 0.0 |
| 101 | 8926.0 | 19165.0 | 17261.0 | 14840.0 | 5632.0 | 8060.0 | 20235.0 | 17982.0 | 18158.0 | 17196.0 | 6351.0 | 16897.0 | 5408.0 | 18150.0 | 8320.0 | 4844.0 | 18558.0 | 15303.0 | 12097.0 | 10045.0 | 21411.0 | 8822.0 | 6575.0 | 3150.0 | 10881.0 | 8027.0 | 13198.0 | 13709.0 | 14922.0 | 7195.0 | 12766.0 | 9840.0 | 8693.0 | 2960.0 | 2205.0 | 9937.0 | 9608.0 | 10708.0 | 9404.0 | 2839.0 | 12761.0 | 14488.0 | 15099.0 | 8797.0 | 8419.0 | 6870.0 | 16517.0 | 17569.0 | 8428.0 | 9457.0 | 9122.0 | 12185.0 | 14411.0 | 9356.0 | 7158.0 | 14822.0 | 13844.0 | 12523.0 | 4124.0 | 7438.0 | 3767.0 | 0.0 | 0.0 | 0.0 | 3748.0 | 9513.0 | 3602.0 | 0.0 | 0.0 | 0.0 | 0.0 | 5211.0 | 5769.0 | 8376.0 | 5813.0 | 7623.0 | 6005.0 | 0.0 | 7800.0 | 7340.0 | 0.0 | 0.0 | 5504.0 | 3992.0 | 1477.0 | 7729.0 | 8788.0 | 0.0 | 0.0 | 1437.0 | 0.0 | 0.0 | 10065.0 | 9183.0 | 4601.0 | 1443.0 | 6094.0 | 0.0 | 0.0 | 0.0 |
| 108 | 15275.0 | 13940.0 | 19408.0 | 12247.0 | 2636.0 | 4161.0 | 21677.0 | 11939.0 | 14145.0 | 12424.0 | 2859.0 | 11435.0 | 2476.0 | 21210.0 | 5190.0 | 2272.0 | 13920.0 | 20331.0 | 6750.0 | 5154.0 | 16176.0 | 4297.0 | 3048.0 | 1273.0 | 5575.0 | 3867.0 | 7802.0 | 8557.0 | 11362.0 | 3513.0 | 6532.0 | 4805.0 | 4033.0 | 1206.0 | 0.0 | 5286.0 | 5114.0 | 5974.0 | 4986.0 | 0.0 | 7042.0 | 8048.0 | 9057.0 | 4546.0 | 4250.0 | 3318.0 | 10952.0 | 11501.0 | 4177.0 | 4704.0 | 4786.0 | 6860.0 | 19606.0 | 4875.0 | 3430.0 | 9217.0 | 7945.0 | 8155.0 | 0.0 | 3540.0 | 0.0 | 0.0 | 0.0 | 0.0 | 0.0 | 4852.0 | 0.0 | 0.0 | 0.0 | 0.0 | 0.0 | 0.0 | 3858.0 | 6254.0 | 4067.0 | 5504.0 | 4191.0 | 0.0 | 5535.0 | 5324.0 | 0.0 | 0.0 | 4226.0 | 2684.0 | 0.0 | 5354.0 | 6142.0 | 0.0 | 0.0 | 0.0 | 0.0 | 0.0 | 7171.0 | 6545.0 | 3052.0 | 0.0 | 4274.0 | 0.0 | 0.0 | 0.0 |
| 115 | 13200.0 | 15104.0 | 17440.0 | 12238.0 | 2895.0 | 4421.0 | 20273.0 | 13460.0 | 14156.0 | 13533.0 | 3305.0 | 12257.0 | 2714.0 | 19440.0 | 4577.0 | 2327.0 | 15274.0 | 17554.0 | 7925.0 | 5728.0 | 18214.0 | 4990.0 | 3453.0 | 1326.0 | 6244.0 | 4346.0 | 8627.0 | 9267.0 | 11386.0 | 3980.0 | 7898.0 | 5377.0 | 4800.0 | 1322.0 | 0.0 | 5769.0 | 5699.0 | 6636.0 | 5653.0 | 0.0 | 8587.0 | 9166.0 | 9902.0 | 4960.0 | 4858.0 | 3675.0 | 12328.0 | 12925.0 | 4622.0 | 5239.0 | 5586.0 | 7671.0 | 17263.0 | 5647.0 | 3905.0 | 9951.0 | 8722.0 | 8400.0 | 0.0 | 4017.0 | 0.0 | 0.0 | 0.0 | 0.0 | 0.0 | 5424.0 | 0.0 | 0.0 | 0.0 | 0.0 | 0.0 | 0.0 | 4409.0 | 6837.0 | 4524.0 | 6368.0 | 4597.0 | 0.0 | 6192.0 | 5932.0 | 0.0 | 0.0 | 4607.0 | 3005.0 | 0.0 | 5760.0 | 6470.0 | 0.0 | 0.0 | 0.0 | 0.0 | 0.0 | 7909.0 | 7241.0 | 3301.0 | 0.0 | 4618.0 | 0.0 | 0.0 | 0.0 |
| 191 | 13478.0 | 14437.0 | 17040.0 | 11578.0 | 2693.0 | 3832.0 | 19697.0 | 12854.0 | 13472.0 | 11729.0 | 3060.0 | 10895.0 | 2346.0 | 19418.0 | 4043.0 | 1951.0 | 12808.0 | 17275.0 | 6997.0 | 5600.0 | 17220.0 | 4536.0 | 2696.0 | 0.0 | 5785.0 | 4460.0 | 8095.0 | 8652.0 | 9827.0 | 2849.0 | 8926.0 | 5270.0 | 4753.0 | 0.0 | 0.0 | 5474.0 | 5187.0 | 5924.0 | 3733.0 | 0.0 | 7932.0 | 8886.0 | 8064.0 | 3185.0 | 4510.0 | 3247.0 | 11110.0 | 11834.0 | 4408.0 | 5239.0 | 4624.0 | 6165.0 | 17043.0 | 3796.0 | 3394.0 | 9168.0 | 8312.0 | 7908.0 | 0.0 | 3495.0 | 0.0 | 0.0 | 0.0 | 0.0 | 0.0 | 4834.0 | 0.0 | 0.0 | 0.0 | 0.0 | 0.0 | 3196.0 | 3572.0 | 5755.0 | 3446.0 | 4947.0 | 3587.0 | 0.0 | 5118.0 | 4820.0 | 0.0 | 0.0 | 3712.0 | 2318.0 | 0.0 | 4223.0 | 4920.0 | 0.0 | 0.0 | 0.0 | 0.0 | 0.0 | 5286.0 | 4777.0 | 2790.0 | 0.0 | 3281.0 | 0.0 | 0.0 | 0.0 |
| 227 | 16025.0 | 11514.0 | 17980.0 | 10370.0 | 1716.0 | 2775.0 | 19985.0 | 9601.0 | 11807.0 | 9426.0 | 1875.0 | 8476.0 | 1602.0 | 21784.0 | 3008.0 | 1600.0 | 10191.0 | 20213.0 | 4987.0 | 3673.0 | 14180.0 | 3193.0 | 1954.0 | 0.0 | 4123.0 | 2865.0 | 5731.0 | 7076.0 | 8224.0 | 1871.0 | 6499.0 | 3381.0 | 3223.0 | 0.0 | 0.0 | 3848.0 | 3709.0 | 4285.0 | 2233.0 | 0.0 | 5680.0 | 6496.0 | 5447.0 | 1916.0 | 3023.0 | 2213.0 | 7900.0 | 8834.0 | 3064.0 | 3455.0 | 3311.0 | 4010.0 | 19229.0 | 2165.0 | 2368.0 | 6637.0 | 5951.0 | 6447.0 | 0.0 | 2198.0 | 0.0 | 0.0 | 0.0 | 0.0 | 0.0 | 3030.0 | 0.0 | 0.0 | 0.0 | 0.0 | 0.0 | 2709.0 | 2997.0 | 4662.0 | 3149.0 | 3986.0 | 2995.0 | 0.0 | 4199.0 | 3961.0 | 0.0 | 0.0 | 2910.0 | 2243.0 | 0.0 | 3852.0 | 4632.0 | 0.0 | 0.0 | 0.0 | 0.0 | 0.0 | 4458.0 | 4175.0 | 2565.0 | 0.0 | 2749.0 | 0.0 | 0.0 | 0.0 |
| 241 | 20786.0 | 8738.0 | 17501.0 | 9316.0 | 1001.0 | 1819.0 | 18236.0 | 7443.0 | 9573.0 | 7413.0 | 1149.0 | 6516.0 | 0.0 | 23647.0 | 1866.0 | 0.0 | 8039.0 | 22949.0 | 3396.0 | 2436.0 | 10626.0 | 2048.0 | 1157.0 | 0.0 | 2683.0 | 1887.0 | 3964.0 | 5284.0 | 6544.0 | 0.0 | 4342.0 | 2292.0 | 2113.0 | 0.0 | 0.0 | 2508.0 | 2446.0 | 2783.0 | 1343.0 | 0.0 | 3868.0 | 4352.0 | 3461.0 | 0.0 | 1914.0 | 1377.0 | 5753.0 | 6185.0 | 1946.0 | 2229.0 | 1943.0 | 2594.0 | 23873.0 | 1234.0 | 1451.0 | 4528.0 | 3999.0 | 4905.0 | 0.0 | 0.0 | 0.0 | 0.0 | 0.0 | 0.0 | 0.0 | 0.0 | 0.0 | 0.0 | 0.0 | 0.0 | 0.0 | 2324.0 | 2602.0 | 4072.0 | 2631.0 | 3486.0 | 2623.0 | 0.0 | 3628.0 | 3313.0 | 0.0 | 0.0 | 2611.0 | 0.0 | 0.0 | 2695.0 | 3323.0 | 0.0 | 0.0 | 0.0 | 0.0 | 0.0 | 3599.0 | 3262.0 | 1761.0 | 0.0 | 1845.0 | 0.0 | 0.0 | 0.0 |
| 251 | 18619.0 | 2915.0 | 6065.0 | 2617.0 | 0.0 | 0.0 | 9049.0 | 2313.0 | 3933.0 | 2473.0 | 0.0 | 2403.0 | 0.0 | 13500.0 | 0.0 | 0.0 | 2685.0 | 11804.0 | 1065.0 | 0.0 | 4429.0 | 0.0 | 0.0 | 0.0 | 0.0 | 0.0 | 1339.0 | 2080.0 | 2538.0 | 0.0 | 1581.0 | 0.0 | 0.0 | 0.0 | 0.0 | 0.0 | 0.0 | 0.0 | 0.0 | 0.0 | 0.0 | 1126.0 | 0.0 | 0.0 | 0.0 | 0.0 | 2219.0 | 1924.0 | 0.0 | 0.0 | 0.0 | 0.0 | 13782.0 | 0.0 | 0.0 | 1921.0 | 0.0 | 1804.0 | 0.0 | 0.0 | 0.0 | 0.0 | 0.0 | 0.0 | 0.0 | 0.0 | 0.0 | 0.0 | 0.0 | 0.0 | 0.0 | 0.0 | 0.0 | 0.0 | 0.0 | 0.0 | 0.0 | 0.0 | 0.0 | 0.0 | 0.0 | 0.0 | 0.0 | 0.0 | 0.0 | 0.0 | 0.0 | 0.0 | 0.0 | 0.0 | 0.0 | 0.0 | 1575.0 | 1394.0 | 0.0 | 0.0 | 0.0 | 0.0 | 0.0 | 0.0 |
| 254 | 19917.0 | 6023.0 | 7961.0 | 3732.0 | 0.0 | 1197.0 | 14790.0 | 4564.0 | 6766.0 | 4854.0 | 0.0 | 4367.0 | 0.0 | 19919.0 | 1225.0 | 0.0 | 5380.0 | 18352.0 | 1987.0 | 1538.0 | 7191.0 | 0.0 | 0.0 | 0.0 | 1724.0 | 0.0 | 2412.0 | 3041.0 | 3492.0 | 0.0 | 2696.0 | 0.0 | 1290.0 | 0.0 | 0.0 | 1398.0 | 1322.0 | 1665.0 | 0.0 | 0.0 | 2133.0 | 2736.0 | 2210.0 | 0.0 | 1116.0 | 0.0 | 3814.0 | 3789.0 | 0.0 | 1538.0 | 1675.0 | 1335.0 | 18551.0 | 0.0 | 0.0 | 3188.0 | 2026.0 | 2414.0 | 0.0 | 0.0 | 0.0 | 0.0 | 0.0 | 0.0 | 0.0 | 0.0 | 0.0 | 0.0 | 0.0 | 0.0 | 0.0 | 0.0 | 0.0 | 0.0 | 0.0 | 0.0 | 0.0 | 0.0 | 0.0 | 0.0 | 0.0 | 0.0 | 0.0 | 0.0 | 0.0 | 0.0 | 0.0 | 0.0 | 0.0 | 0.0 | 0.0 | 0.0 | 2241.0 | 2036.0 | 1533.0 | 0.0 | 0.0 | 0.0 | 0.0 | 0.0 |
| 258 | 17454.0 | 13831.0 | 12189.0 | 10030.0 | 3408.0 | 3751.0 | 20616.0 | 11732.0 | 11669.0 | 8801.0 | 3241.0 | 7959.0 | 2657.0 | 22639.0 | 3244.0 | 2070.0 | 9803.0 | 21446.0 | 3722.0 | 0.0 | 11145.0 | 0.0 | 1383.0 | 0.0 | 0.0 | 0.0 | 2109.0 | 4981.0 | 3467.0 | 0.0 | 5365.0 | 1877.0 | 0.0 | 0.0 | 0.0 | 0.0 | 0.0 | 1298.0 | 1405.0 | 0.0 | 4021.0 | 4588.0 | 2410.0 | 1289.0 | 0.0 | 0.0 | 4293.0 | 5976.0 | 0.0 | 0.0 | 3100.0 | 1619.0 | 20294.0 | 1369.0 | 0.0 | 2583.0 | 4040.0 | 2503.0 | 0.0 | 1211.0 | 0.0 | 0.0 | 0.0 | 0.0 | 0.0 | 1837.0 | 0.0 | 0.0 | 0.0 | 0.0 | 0.0 | 0.0 | 0.0 | 0.0 | 0.0 | 0.0 | 0.0 | 0.0 | 0.0 | 0.0 | 0.0 | 0.0 | 0.0 | 0.0 | 0.0 | 0.0 | 0.0 | 0.0 | 0.0 | 0.0 | 0.0 | 0.0 | 2492.0 | 2309.0 | 3340.0 | 0.0 | 1529.0 | 0.0 | 0.0 | 0.0 |
| 261 | 17032.0 | 12006.0 | 12524.0 | 9582.0 | 2751.0 | 2984.0 | 18009.0 | 10091.0 | 10411.0 | 7730.0 | 2845.0 | 7083.0 | 2280.0 | 20964.0 | 2776.0 | 1761.0 | 8208.0 | 19611.0 | 4762.0 | 0.0 | 9276.0 | 0.0 | 1269.0 | 0.0 | 0.0 | 0.0 | 1758.0 | 4251.0 | 3489.0 | 0.0 | 4277.0 | 1465.0 | 0.0 | 0.0 | 0.0 | 0.0 | 0.0 | 0.0 | 1021.0 | 0.0 | 3061.0 | 3555.0 | 1657.0 | 0.0 | 0.0 | 0.0 | 3643.0 | 4926.0 | 0.0 | 0.0 | 2373.0 | 1374.0 | 17638.0 | 0.0 | 0.0 | 1912.0 | 3184.0 | 2371.0 | 0.0 | 0.0 | 0.0 | 0.0 | 0.0 | 0.0 | 0.0 | 0.0 | 0.0 | 0.0 | 0.0 | 0.0 | 0.0 | 0.0 | 0.0 | 0.0 | 0.0 | 0.0 | 0.0 | 0.0 | 0.0 | 0.0 | 0.0 | 0.0 | 0.0 | 0.0 | 0.0 | 0.0 | 0.0 | 0.0 | 0.0 | 0.0 | 0.0 | 0.0 | 1851.0 | 1641.0 | 2228.0 | 0.0 | 1073.0 | 0.0 | 0.0 | 0.0 |
| 265 | 21445.0 | 13085.0 | 11389.0 | 9589.0 | 3078.0 | 3418.0 | 19806.0 | 10614.0 | 11182.0 | 8870.0 | 2730.0 | 8287.0 | 2639.0 | 23726.0 | 3067.0 | 2074.0 | 9193.0 | 22929.0 | 3325.0 | 0.0 | 9882.0 | 0.0 | 1737.0 | 0.0 | 0.0 | 0.0 | 2372.0 | 4652.0 | 3772.0 | 0.0 | 4872.0 | 2039.0 | 0.0 | 0.0 | 0.0 | 0.0 | 1384.0 | 1424.0 | 1441.0 | 0.0 | 3716.0 | 4201.0 | 1814.0 | 0.0 | 0.0 | 0.0 | 5121.0 | 5627.0 | 0.0 | 0.0 | 2982.0 | 1411.0 | 19725.0 | 0.0 | 0.0 | 2597.0 | 3917.0 | 2896.0 | 0.0 | 0.0 | 0.0 | 0.0 | 0.0 | 0.0 | 0.0 | 0.0 | 0.0 | 0.0 | 0.0 | 0.0 | 0.0 | 0.0 | 0.0 | 0.0 | 0.0 | 0.0 | 0.0 | 0.0 | 0.0 | 0.0 | 0.0 | 0.0 | 0.0 | 0.0 | 0.0 | 0.0 | 1385.0 | 0.0 | 0.0 | 0.0 | 0.0 | 0.0 | 2391.0 | 2258.0 | 2935.0 | 0.0 | 2307.0 | 0.0 | 0.0 | 0.0 |
| 268 | 12716.0 | 15366.0 | 15327.0 | 12894.0 | 4185.0 | 5048.0 | 20390.0 | 13850.0 | 13747.0 | 11011.0 | 3947.0 | 10520.0 | 3546.0 | 20352.0 | 4397.0 | 3070.0 | 11897.0 | 19279.0 | 4910.0 | 0.0 | 12253.0 | 0.0 | 2607.0 | 0.0 | 0.0 | 0.0 | 3099.0 | 6290.0 | 4401.0 | 1491.0 | 6674.0 | 3103.0 | 0.0 | 0.0 | 0.0 | 1507.0 | 1753.0 | 1814.0 | 1957.0 | 0.0 | 4806.0 | 5305.0 | 2513.0 | 1823.0 | 0.0 | 0.0 | 6919.0 | 7160.0 | 0.0 | 0.0 | 3781.0 | 2054.0 | 21367.0 | 1894.0 | 0.0 | 3477.0 | 5370.0 | 3893.0 | 0.0 | 0.0 | 0.0 | 0.0 | 0.0 | 0.0 | 0.0 | 2261.0 | 0.0 | 0.0 | 0.0 | 0.0 | 0.0 | 0.0 | 0.0 | 0.0 | 0.0 | 0.0 | 0.0 | 0.0 | 0.0 | 2687.0 | 0.0 | 0.0 | 0.0 | 0.0 | 0.0 | 1915.0 | 2312.0 | 0.0 | 0.0 | 0.0 | 0.0 | 0.0 | 3461.0 | 3218.0 | 3883.0 | 0.0 | 2867.0 | 0.0 | 0.0 | 0.0 |
| 272 | 19601.0 | 13711.0 | 14870.0 | 11493.0 | 3394.0 | 3970.0 | 20536.0 | 11828.0 | 12079.0 | 9700.0 | 3463.0 | 9513.0 | 2845.0 | 23512.0 | 3307.0 | 2523.0 | 10727.0 | 22431.0 | 4099.0 | 1412.0 | 10759.0 | 0.0 | 2234.0 | 0.0 | 1651.0 | 0.0 | 2975.0 | 5309.0 | 3926.0 | 0.0 | 5740.0 | 2610.0 | 0.0 | 0.0 | 0.0 | 1521.0 | 1703.0 | 1881.0 | 1815.0 | 0.0 | 3747.0 | 4591.0 | 2483.0 | 1677.0 | 0.0 | 0.0 | 6614.0 | 6110.0 | 0.0 | 0.0 | 2781.0 | 1945.0 | 20511.0 | 1774.0 | 0.0 | 3331.0 | 4553.0 | 3433.0 | 0.0 | 0.0 | 0.0 | 0.0 | 0.0 | 0.0 | 0.0 | 0.0 | 0.0 | 0.0 | 0.0 | 0.0 | 0.0 | 0.0 | 0.0 | 0.0 | 0.0 | 0.0 | 0.0 | 0.0 | 0.0 | 0.0 | 0.0 | 0.0 | 0.0 | 0.0 | 0.0 | 1534.0 | 1805.0 | 0.0 | 0.0 | 0.0 | 0.0 | 0.0 | 2136.0 | 1984.0 | 2120.0 | 0.0 | 1717.0 | 0.0 | 0.0 | 0.0 |
| 279 | 19807.0 | 5980.0 | 6730.0 | 4914.0 | 0.0 | 1337.0 | 10639.0 | 5172.0 | 5638.0 | 4197.0 | 1193.0 | 3871.0 | 0.0 | 14044.0 | 0.0 | 0.0 | 4679.0 | 13713.0 | 2651.0 | 1350.0 | 6073.0 | 0.0 | 0.0 | 0.0 | 1777.0 | 0.0 | 2112.0 | 2517.0 | 2074.0 | 0.0 | 3139.0 | 1614.0 | 0.0 | 0.0 | 0.0 | 1225.0 | 0.0 | 1422.0 | 0.0 | 0.0 | 2020.0 | 2954.0 | 1531.0 | 0.0 | 0.0 | 0.0 | 5009.0 | 3856.0 | 0.0 | 0.0 | 0.0 | 1321.0 | 10736.0 | 0.0 | 0.0 | 2502.0 | 2578.0 | 2079.0 | 0.0 | 0.0 | 0.0 | 0.0 | 0.0 | 0.0 | 0.0 | 0.0 | 0.0 | 0.0 | 0.0 | 0.0 | 0.0 | 0.0 | 0.0 | 0.0 | 0.0 | 0.0 | 0.0 | 0.0 | 0.0 | 0.0 | 0.0 | 0.0 | 0.0 | 0.0 | 0.0 | 2361.0 | 2674.0 | 0.0 | 0.0 | 0.0 | 0.0 | 0.0 | 0.0 | 0.0 | 0.0 | 0.0 | 0.0 | 0.0 | 0.0 | 0.0 |
| 286 | 19381.0 | 9737.0 | 11224.0 | 8536.0 | 2742.0 | 2943.0 | 14523.0 | 8933.0 | 8742.0 | 7320.0 | 2777.0 | 7361.0 | 2464.0 | 17894.0 | 2441.0 | 2149.0 | 7833.0 | 17667.0 | 4400.0 | 3526.0 | 11735.0 | 0.0 | 2473.0 | 2560.0 | 3128.0 | 0.0 | 4193.0 | 4765.0 | 3369.0 | 2010.0 | 7497.0 | 5736.0 | 0.0 | 1757.0 | 0.0 | 2437.0 | 2346.0 | 2781.0 | 1931.0 | 0.0 | 5818.0 | 7479.0 | 2966.0 | 1738.0 | 2256.0 | 1809.0 | 10063.0 | 8512.0 | 1484.0 | 1437.0 | 2708.0 | 2501.0 | 16398.0 | 1882.0 | 1582.0 | 5609.0 | 7105.0 | 3591.0 | 0.0 | 0.0 | 0.0 | 0.0 | 0.0 | 0.0 | 0.0 | 2542.0 | 0.0 | 0.0 | 0.0 | 0.0 | 0.0 | 0.0 | 0.0 | 2337.0 | 0.0 | 0.0 | 0.0 | 0.0 | 0.0 | 2508.0 | 0.0 | 0.0 | 0.0 | 0.0 | 1411.0 | 6815.0 | 7643.0 | 0.0 | 0.0 | 0.0 | 0.0 | 0.0 | 1614.0 | 1539.0 | 1391.0 | 0.0 | 0.0 | 0.0 | 0.0 | 0.0 |
| 293 | 15075.0 | 13273.0 | 15084.0 | 12093.0 | 3812.0 | 4190.0 | 17922.0 | 12095.0 | 11464.0 | 9799.0 | 3993.0 | 10046.0 | 3604.0 | 21109.0 | 3588.0 | 3105.0 | 10811.0 | 19895.0 | 6494.0 | 6192.0 | 15950.0 | 0.0 | 3967.0 | 4606.0 | 4952.0 | 1837.0 | 5765.0 | 6689.0 | 4586.0 | 3124.0 | 10728.0 | 8222.0 | 0.0 | 2392.0 | 0.0 | 3434.0 | 3119.0 | 3544.0 | 2843.0 | 2297.0 | 8633.0 | 10506.0 | 4331.0 | 2500.0 | 3741.0 | 2951.0 | 14099.0 | 11932.0 | 2065.0 | 2106.0 | 4034.0 | 3698.0 | 19174.0 | 2786.0 | 2368.0 | 9236.0 | 10162.0 | 4537.0 | 0.0 | 2690.0 | 0.0 | 0.0 | 0.0 | 0.0 | 0.0 | 3731.0 | 0.0 | 0.0 | 0.0 | 0.0 | 0.0 | 0.0 | 2367.0 | 3795.0 | 3012.0 | 3295.0 | 0.0 | 0.0 | 3391.0 | 3797.0 | 0.0 | 0.0 | 2869.0 | 0.0 | 2658.0 | 13096.0 | 14616.0 | 0.0 | 0.0 | 0.0 | 0.0 | 0.0 | 2378.0 | 2230.0 | 1565.0 | 0.0 | 1852.0 | 0.0 | 0.0 | 0.0 |
| 296 | 21459.0 | 17052.0 | 17060.0 | 14413.0 | 6477.0 | 6599.0 | 20904.0 | 15604.0 | 14714.0 | 12869.0 | 6450.0 | 13090.0 | 5984.0 | 24659.0 | 5325.0 | 5234.0 | 14647.0 | 23722.0 | 8476.0 | 8488.0 | 19168.0 | 2541.0 | 5262.0 | 6579.0 | 7375.0 | 3341.0 | 8855.0 | 9301.0 | 5962.0 | 4941.0 | 13437.0 | 10568.0 | 2795.0 | 3646.0 | 0.0 | 5794.0 | 5113.0 | 6044.0 | 4165.0 | 3588.0 | 11175.0 | 14204.0 | 6809.0 | 3941.0 | 5378.0 | 4489.0 | 17239.0 | 16098.0 | 3430.0 | 3670.0 | 6692.0 | 5825.0 | 22256.0 | 4325.0 | 3928.0 | 13069.0 | 13358.0 | 6425.0 | 3431.0 | 4642.0 | 0.0 | 0.0 | 0.0 | 0.0 | 0.0 | 6308.0 | 4186.0 | 0.0 | 0.0 | 0.0 | 0.0 | 0.0 | 3728.0 | 5181.0 | 4525.0 | 4425.0 | 4262.0 | 0.0 | 4657.0 | 4841.0 | 0.0 | 0.0 | 3699.0 | 3095.0 | 5196.0 | 16836.0 | 18138.0 | 2593.0 | 0.0 | 0.0 | 0.0 | 0.0 | 3303.0 | 3047.0 | 0.0 | 0.0 | 0.0 | 0.0 | 0.0 | 0.0 |
| 300 | 23061.0 | 18861.0 | 18952.0 | 15944.0 | 6045.0 | 5323.0 | 24051.0 | 17006.0 | 15621.0 | 13974.0 | 6318.0 | 13191.0 | 5117.0 | 27440.0 | 4633.0 | 4283.0 | 14995.0 | 26702.0 | 7770.0 | 7263.0 | 21603.0 | 1788.0 | 4413.0 | 4115.0 | 5601.0 | 2265.0 | 7787.0 | 8767.0 | 5398.0 | 4826.0 | 13816.0 | 10662.0 | 1925.0 | 2431.0 | 0.0 | 5315.0 | 4253.0 | 5081.0 | 4226.0 | 3384.0 | 12161.0 | 15345.0 | 6541.0 | 3872.0 | 5117.0 | 3916.0 | 17502.0 | 17245.0 | 2545.0 | 2816.0 | 5421.0 | 5372.0 | 25167.0 | 4488.0 | 3020.0 | 13999.0 | 14024.0 | 5543.0 | 2756.0 | 4465.0 | 0.0 | 0.0 | 0.0 | 0.0 | 0.0 | 6232.0 | 3484.0 | 0.0 | 0.0 | 0.0 | 0.0 | 0.0 | 3648.0 | 5447.0 | 4736.0 | 4728.0 | 4486.0 | 0.0 | 4836.0 | 5546.0 | 0.0 | 0.0 | 4034.0 | 3090.0 | 6351.0 | 21063.0 | 22599.0 | 0.0 | 1858.0 | 2152.0 | 0.0 | 0.0 | 3961.0 | 3727.0 | 3021.0 | 0.0 | 3686.0 | 1740.0 | 1902.0 | 0.0 |
| 307 | 13409.0 | 19735.0 | 18928.0 | 16808.0 | 6983.0 | 6299.0 | 22586.0 | 17945.0 | 15984.0 | 14510.0 | 7480.0 | 13901.0 | 5870.0 | 22915.0 | 4983.0 | 4895.0 | 15766.0 | 20972.0 | 8228.0 | 7986.0 | 22045.0 | 1747.0 | 4934.0 | 4343.0 | 6083.0 | 2424.0 | 8654.0 | 9031.0 | 5852.0 | 5882.0 | 14560.0 | 11457.0 | 2004.0 | 2843.0 | 0.0 | 5991.0 | 4548.0 | 5694.0 | 5244.0 | 4124.0 | 13015.0 | 16427.0 | 7615.0 | 4714.0 | 5814.0 | 4478.0 | 17786.0 | 18339.0 | 2833.0 | 3279.0 | 5890.0 | 6319.0 | 22300.0 | 5411.0 | 3479.0 | 15348.0 | 15086.0 | 5628.0 | 3162.0 | 5298.0 | 0.0 | 0.0 | 0.0 | 0.0 | 0.0 | 7130.0 | 3804.0 | 0.0 | 0.0 | 0.0 | 0.0 | 0.0 | 4864.0 | 7334.0 | 5842.0 | 6121.0 | 5965.0 | 0.0 | 6130.0 | 6620.0 | 0.0 | 0.0 | 5521.0 | 3652.0 | 9095.0 | 21741.0 | 22345.0 | 0.0 | 1690.0 | 2070.0 | 0.0 | 0.0 | 4908.0 | 4454.0 | 3210.0 | 0.0 | 3976.0 | 0.0 | 1729.0 | 0.0 |
| 321 | 20385.0 | 13643.0 | 14537.0 | 11501.0 | 3629.0 | 3323.0 | 19144.0 | 12183.0 | 11099.0 | 9689.0 | 4057.0 | 10006.0 | 2968.0 | 22778.0 | 2650.0 | 2635.0 | 10882.0 | 23353.0 | 6372.0 | 5958.0 | 17396.0 | 0.0 | 4006.0 | 3604.0 | 4161.0 | 1536.0 | 5424.0 | 6500.0 | 3489.0 | 3622.0 | 11559.0 | 9113.0 | 0.0 | 1733.0 | 0.0 | 3854.0 | 2720.0 | 3319.0 | 2865.0 | 1890.0 | 9601.0 | 11796.0 | 4337.0 | 2459.0 | 3612.0 | 2579.0 | 14733.0 | 13144.0 | 1754.0 | 1877.0 | 3565.0 | 3646.0 | 20673.0 | 2994.0 | 2010.0 | 10839.0 | 11290.0 | 3787.0 | 0.0 | 3675.0 | 0.0 | 0.0 | 0.0 | 0.0 | 0.0 | 4855.0 | 0.0 | 0.0 | 0.0 | 0.0 | 0.0 | 0.0 | 0.0 | 3461.0 | 2788.0 | 2992.0 | 0.0 | 0.0 | 2923.0 | 3518.0 | 0.0 | 0.0 | 2951.0 | 0.0 | 5298.0 | 19406.0 | 20487.0 | 0.0 | 0.0 | 0.0 | 0.0 | 0.0 | 2119.0 | 2091.0 | 1320.0 | 0.0 | 1858.0 | 0.0 | 0.0 | 0.0 |
| 328 | 23007.0 | 8102.0 | 9441.0 | 7883.0 | 1770.0 | 2292.0 | 12946.0 | 6421.0 | 7394.0 | 5861.0 | 2177.0 | 5416.0 | 1913.0 | 16500.0 | 2029.0 | 1959.0 | 6212.0 | 17936.0 | 4969.0 | 4800.0 | 12040.0 | 1632.0 | 3507.0 | 2482.0 | 3411.0 | 1805.0 | 5220.0 | 3946.0 | 3229.0 | 2880.0 | 8277.0 | 6746.0 | 0.0 | 1484.0 | 0.0 | 3453.0 | 3029.0 | 2735.0 | 1749.0 | 1176.0 | 6783.0 | 7710.0 | 2813.0 | 1764.0 | 3350.0 | 2309.0 | 10940.0 | 9766.0 | 2085.0 | 1659.0 | 2928.0 | 2896.0 | 12957.0 | 1844.0 | 1943.0 | 8638.0 | 9446.0 | 3140.0 | 2049.0 | 3149.0 | 0.0 | 0.0 | 0.0 | 0.0 | 0.0 | 4391.0 | 2262.0 | 0.0 | 0.0 | 0.0 | 0.0 | 2154.0 | 0.0 | 3328.0 | 2816.0 | 3009.0 | 2512.0 | 0.0 | 2961.0 | 3234.0 | 0.0 | 0.0 | 2131.0 | 0.0 | 3872.0 | 15835.0 | 17026.0 | 0.0 | 0.0 | 0.0 | 0.0 | 0.0 | 1748.0 | 1625.0 | 1572.0 | 0.0 | 1752.0 | 0.0 | 1306.0 | 0.0 |
| 336 | 22381.0 | 9560.0 | 12089.0 | 9733.0 | 1874.0 | 2312.0 | 15306.0 | 7969.0 | 8833.0 | 7380.0 | 2367.0 | 6522.0 | 1858.0 | 19016.0 | 2109.0 | 1753.0 | 7772.0 | 20012.0 | 5596.0 | 4127.0 | 13737.0 | 0.0 | 3384.0 | 2485.0 | 2261.0 | 0.0 | 4238.0 | 4522.0 | 3046.0 | 2091.0 | 9603.0 | 7582.0 | 0.0 | 1788.0 | 0.0 | 2517.0 | 2072.0 | 1696.0 | 1985.0 | 0.0 | 7656.0 | 8490.0 | 2887.0 | 2058.0 | 2265.0 | 1470.0 | 10963.0 | 9836.0 | 1143.0 | 0.0 | 2768.0 | 2761.0 | 16094.0 | 2083.0 | 0.0 | 7879.0 | 10545.0 | 2425.0 | 0.0 | 2217.0 | 0.0 | 0.0 | 0.0 | 0.0 | 0.0 | 3248.0 | 0.0 | 0.0 | 0.0 | 0.0 | 0.0 | 0.0 | 0.0 | 0.0 | 0.0 | 0.0 | 0.0 | 0.0 | 0.0 | 0.0 | 0.0 | 0.0 | 0.0 | 0.0 | 0.0 | 6832.0 | 7387.0 | 0.0 | 0.0 | 0.0 | 0.0 | 0.0 | 1450.0 | 1527.0 | 0.0 | 0.0 | 1310.0 | 0.0 | 0.0 | 0.0 |
| 344 | 17627.0 | 11112.0 | 12951.0 | 10422.0 | 2235.0 | 2721.0 | 16112.0 | 9140.0 | 9892.0 | 8503.0 | 2827.0 | 7726.0 | 2343.0 | 20299.0 | 2463.0 | 2084.0 | 9442.0 | 20105.0 | 5736.0 | 3956.0 | 14146.0 | 0.0 | 3798.0 | 2566.0 | 2503.0 | 0.0 | 4235.0 | 4807.0 | 3265.0 | 2120.0 | 10078.0 | 8016.0 | 0.0 | 1854.0 | 0.0 | 2382.0 | 1973.0 | 1693.0 | 2349.0 | 0.0 | 7997.0 | 8740.0 | 3166.0 | 2290.0 | 2203.0 | 1466.0 | 10583.0 | 10073.0 | 1206.0 | 0.0 | 2941.0 | 3112.0 | 17097.0 | 2416.0 | 0.0 | 7746.0 | 10988.0 | 2689.0 | 0.0 | 2230.0 | 0.0 | 0.0 | 0.0 | 0.0 | 0.0 | 3192.0 | 0.0 | 0.0 | 0.0 | 0.0 | 0.0 | 0.0 | 0.0 | 0.0 | 0.0 | 0.0 | 0.0 | 0.0 | 0.0 | 0.0 | 0.0 | 0.0 | 0.0 | 0.0 | 0.0 | 3362.0 | 3572.0 | 0.0 | 0.0 | 0.0 | 0.0 | 0.0 | 1626.0 | 1462.0 | 0.0 | 0.0 | 0.0 | 0.0 | 0.0 | 0.0 |
| 349 | 20717.0 | 13919.0 | 16594.0 | 12718.0 | 3153.0 | 2998.0 | 18631.0 | 11546.0 | 11876.0 | 10747.0 | 3845.0 | 9456.0 | 3059.0 | 23693.0 | 2831.0 | 2436.0 | 10966.0 | 23355.0 | 6255.0 | 4286.0 | 17813.0 | 0.0 | 3387.0 | 2750.0 | 3079.0 | 1220.0 | 5529.0 | 6251.0 | 4357.0 | 2613.0 | 12034.0 | 10423.0 | 0.0 | 1613.0 | 0.0 | 3004.0 | 2702.0 | 2323.0 | 3242.0 | 1361.0 | 10395.0 | 11748.0 | 4661.0 | 3245.0 | 2746.0 | 1842.0 | 13049.0 | 13339.0 | 1460.0 | 1216.0 | 3760.0 | 3786.0 | 20970.0 | 3254.0 | 1417.0 | 9186.0 | 13841.0 | 3471.0 | 0.0 | 2848.0 | 0.0 | 0.0 | 0.0 | 0.0 | 0.0 | 3958.0 | 0.0 | 0.0 | 0.0 | 0.0 | 0.0 | 0.0 | 0.0 | 0.0 | 0.0 | 0.0 | 0.0 | 0.0 | 0.0 | 0.0 | 0.0 | 0.0 | 0.0 | 0.0 | 0.0 | 3809.0 | 4004.0 | 0.0 | 0.0 | 0.0 | 0.0 | 0.0 | 1997.0 | 1863.0 | 0.0 | 0.0 | 1506.0 | 0.0 | 0.0 | 0.0 |
| 352 | 18790.0 | 15866.0 | 18861.0 | 14940.0 | 3762.0 | 4626.0 | 21797.0 | 13575.0 | 14462.0 | 12740.0 | 4752.0 | 11632.0 | 3904.0 | 25057.0 | 3949.0 | 3423.0 | 13823.0 | 24801.0 | 8324.0 | 6214.0 | 18799.0 | 1227.0 | 5537.0 | 4404.0 | 4228.0 | 1510.0 | 6515.0 | 7247.0 | 5630.0 | 3237.0 | 13862.0 | 11851.0 | 0.0 | 2302.0 | 0.0 | 3696.0 | 3384.0 | 2800.0 | 3813.0 | 1433.0 | 11459.0 | 12557.0 | 5344.0 | 3769.0 | 3378.0 | 2153.0 | 15592.0 | 14742.0 | 1843.0 | 1489.0 | 4504.0 | 4925.0 | 22235.0 | 4028.0 | 1773.0 | 11128.0 | 15955.0 | 4305.0 | 0.0 | 3335.0 | 0.0 | 0.0 | 0.0 | 0.0 | 0.0 | 4615.0 | 0.0 | 0.0 | 0.0 | 0.0 | 0.0 | 0.0 | 0.0 | 2117.0 | 0.0 | 0.0 | 2094.0 | 0.0 | 0.0 | 0.0 | 0.0 | 0.0 | 0.0 | 0.0 | 0.0 | 3591.0 | 3661.0 | 0.0 | 0.0 | 0.0 | 0.0 | 0.0 | 1887.0 | 1759.0 | 0.0 | 0.0 | 1379.0 | 0.0 | 0.0 | 0.0 |
| 358 | 22387.0 | 12008.0 | 13783.0 | 10118.0 | 2653.0 | 3361.0 | 18983.0 | 10314.0 | 11059.0 | 10176.0 | 3623.0 | 9101.0 | 2679.0 | 21430.0 | 2865.0 | 2496.0 | 10753.0 | 22063.0 | 6598.0 | 5114.0 | 17178.0 | 0.0 | 4079.0 | 3212.0 | 3149.0 | 1198.0 | 5919.0 | 5808.0 | 4643.0 | 2174.0 | 11950.0 | 10193.0 | 0.0 | 2767.0 | 0.0 | 2804.0 | 2662.0 | 2214.0 | 2898.0 | 1079.0 | 10054.0 | 11085.0 | 4300.0 | 2806.0 | 2496.0 | 1629.0 | 13106.0 | 12728.0 | 1507.0 | 1190.0 | 3813.0 | 3655.0 | 21047.0 | 3021.0 | 1315.0 | 9407.0 | 13826.0 | 3080.0 | 0.0 | 2511.0 | 0.0 | 0.0 | 0.0 | 0.0 | 0.0 | 3517.0 | 0.0 | 0.0 | 0.0 | 0.0 | 0.0 | 2004.0 | 0.0 | 0.0 | 0.0 | 0.0 | 0.0 | 0.0 | 0.0 | 0.0 | 0.0 | 0.0 | 0.0 | 0.0 | 0.0 | 3983.0 | 4129.0 | 0.0 | 0.0 | 0.0 | 0.0 | 0.0 | 1820.0 | 1640.0 | 0.0 | 0.0 | 1503.0 | 0.0 | 0.0 | 0.0 |
| 364 | 17241.0 | 14395.0 | 18444.0 | 13944.0 | 2594.0 | 3490.0 | 21136.0 | 11478.0 | 13160.0 | 11427.0 | 3518.0 | 10028.0 | 2840.0 | 24712.0 | 3158.0 | 2496.0 | 12555.0 | 23510.0 | 7207.0 | 4923.0 | 17097.0 | 0.0 | 4681.0 | 3170.0 | 2909.0 | 0.0 | 5322.0 | 5973.0 | 4624.0 | 2387.0 | 11994.0 | 9715.0 | 0.0 | 1485.0 | 0.0 | 2772.0 | 2407.0 | 1993.0 | 2889.0 | 0.0 | 9680.0 | 10489.0 | 4243.0 | 3072.0 | 2363.0 | 1482.0 | 13732.0 | 12528.0 | 1325.0 | 1037.0 | 3392.0 | 3788.0 | 22440.0 | 3258.0 | 1186.0 | 9236.0 | 13455.0 | 3283.0 | 0.0 | 2550.0 | 0.0 | 0.0 | 0.0 | 0.0 | 0.0 | 3784.0 | 0.0 | 0.0 | 0.0 | 0.0 | 0.0 | 0.0 | 0.0 | 0.0 | 0.0 | 0.0 | 0.0 | 0.0 | 0.0 | 0.0 | 0.0 | 0.0 | 0.0 | 0.0 | 0.0 | 3998.0 | 4184.0 | 0.0 | 0.0 | 0.0 | 0.0 | 0.0 | 2154.0 | 1977.0 | 0.0 | 0.0 | 1783.0 | 0.0 | 0.0 | 0.0 |
| 371 | 17018.0 | 13795.0 | 18623.0 | 13740.0 | 2627.0 | 3590.0 | 20997.0 | 11776.0 | 13082.0 | 11033.0 | 3569.0 | 9774.0 | 2806.0 | 25603.0 | 3178.0 | 2522.0 | 12095.0 | 23671.0 | 7450.0 | 4826.0 | 17253.0 | 0.0 | 4875.0 | 3568.0 | 3003.0 | 0.0 | 5163.0 | 6138.0 | 4774.0 | 2219.0 | 12277.0 | 9993.0 | 0.0 | 2641.0 | 0.0 | 2529.0 | 2282.0 | 1772.0 | 2837.0 | 0.0 | 9931.0 | 10884.0 | 4158.0 | 2947.0 | 2187.0 | 1342.0 | 13505.0 | 12752.0 | 1105.0 | 0.0 | 3690.0 | 3792.0 | 22022.0 | 3170.0 | 0.0 | 9286.0 | 14014.0 | 3199.0 | 0.0 | 2359.0 | 0.0 | 0.0 | 0.0 | 0.0 | 0.0 | 3472.0 | 0.0 | 0.0 | 0.0 | 0.0 | 0.0 | 0.0 | 0.0 | 0.0 | 0.0 | 0.0 | 0.0 | 0.0 | 0.0 | 0.0 | 0.0 | 0.0 | 0.0 | 0.0 | 0.0 | 3010.0 | 3053.0 | 0.0 | 0.0 | 0.0 | 0.0 | 0.0 | 1930.0 | 1744.0 | 0.0 | 0.0 | 1565.0 | 0.0 | 0.0 | 0.0 |
| 378 | 7516.0 | 18821.0 | 19535.0 | 15422.0 | 5214.0 | 6399.0 | 22080.0 | 17023.0 | 17454.0 | 16386.0 | 6474.0 | 14934.0 | 5441.0 | 22694.0 | 5595.0 | 4891.0 | 17193.0 | 17368.0 | 10726.0 | 6928.0 | 22259.0 | 1195.0 | 7683.0 | 5237.0 | 4818.0 | 1816.0 | 8451.0 | 9558.0 | 6915.0 | 3789.0 | 17218.0 | 14982.0 | 0.0 | 2452.0 | 0.0 | 4338.0 | 4102.0 | 3396.0 | 4809.0 | 1828.0 | 14599.0 | 15811.0 | 6718.0 | 5066.0 | 3732.0 | 2329.0 | 16603.0 | 17405.0 | 2088.0 | 1599.0 | 6291.0 | 6258.0 | 23342.0 | 5276.0 | 1818.0 | 13479.0 | 19261.0 | 5252.0 | 0.0 | 4212.0 | 0.0 | 0.0 | 0.0 | 0.0 | 0.0 | 5870.0 | 0.0 | 0.0 | 0.0 | 0.0 | 0.0 | 0.0 | 0.0 | 0.0 | 0.0 | 0.0 | 0.0 | 0.0 | 0.0 | 0.0 | 0.0 | 0.0 | 0.0 | 0.0 | 0.0 | 3435.0 | 3641.0 | 0.0 | 0.0 | 0.0 | 0.0 | 0.0 | 2358.0 | 1906.0 | 0.0 | 0.0 | 1606.0 | 0.0 | 0.0 | 0.0 |
| 380 | 16197.0 | 13238.0 | 17503.0 | 13058.0 | 2579.0 | 3304.0 | 20021.0 | 10797.0 | 12665.0 | 10858.0 | 3319.0 | 9459.0 | 2739.0 | 23344.0 | 3102.0 | 2248.0 | 11378.0 | 22491.0 | 6825.0 | 4374.0 | 16656.0 | 0.0 | 3983.0 | 3410.0 | 2849.0 | 0.0 | 4755.0 | 5636.0 | 4868.0 | 1923.0 | 11398.0 | 9269.0 | 0.0 | 2519.0 | 0.0 | 2133.0 | 2090.0 | 1627.0 | 2608.0 | 0.0 | 9218.0 | 10505.0 | 3603.0 | 2669.0 | 1861.0 | 1227.0 | 13075.0 | 12223.0 | 1001.0 | 0.0 | 3220.0 | 3428.0 | 21701.0 | 2935.0 | 0.0 | 8318.0 | 13190.0 | 3085.0 | 0.0 | 2053.0 | 0.0 | 0.0 | 0.0 | 0.0 | 0.0 | 2931.0 | 0.0 | 0.0 | 0.0 | 0.0 | 0.0 | 0.0 | 0.0 | 0.0 | 0.0 | 0.0 | 0.0 | 0.0 | 0.0 | 0.0 | 0.0 | 0.0 | 0.0 | 0.0 | 0.0 | 2594.0 | 2637.0 | 0.0 | 0.0 | 0.0 | 0.0 | 0.0 | 1922.0 | 1713.0 | 0.0 | 0.0 | 1505.0 | 0.0 | 0.0 | 0.0 |
| 385 | 9420.0 | 14989.0 | 17692.0 | 13328.0 | 3162.0 | 3991.0 | 20504.0 | 12693.0 | 13609.0 | 12298.0 | 4005.0 | 10899.0 | 3309.0 | 22550.0 | 3695.0 | 2849.0 | 13113.0 | 19619.0 | 7566.0 | 4635.0 | 18101.0 | 0.0 | 4708.0 | 3583.0 | 3012.0 | 0.0 | 5442.0 | 6446.0 | 5235.0 | 2399.0 | 12932.0 | 10896.0 | 0.0 | 1750.0 | 0.0 | 2502.0 | 2414.0 | 1880.0 | 3118.0 | 0.0 | 10576.0 | 11606.0 | 4558.0 | 3222.0 | 2113.0 | 1400.0 | 13407.0 | 13268.0 | 1141.0 | 0.0 | 3786.0 | 4081.0 | 21897.0 | 3414.0 | 0.0 | 9338.0 | 14649.0 | 3561.0 | 0.0 | 2481.0 | 0.0 | 0.0 | 0.0 | 0.0 | 0.0 | 3624.0 | 0.0 | 0.0 | 0.0 | 0.0 | 0.0 | 0.0 | 0.0 | 0.0 | 0.0 | 0.0 | 0.0 | 0.0 | 0.0 | 0.0 | 0.0 | 0.0 | 0.0 | 0.0 | 0.0 | 2129.0 | 2054.0 | 0.0 | 0.0 | 0.0 | 0.0 | 0.0 | 2025.0 | 1854.0 | 0.0 | 0.0 | 1565.0 | 0.0 | 0.0 | 0.0 |
| 392 | 11240.0 | 13435.0 | 18182.0 | 12968.0 | 2706.0 | 3765.0 | 20431.0 | 11673.0 | 13083.0 | 11718.0 | 3470.0 | 10259.0 | 2991.0 | 23136.0 | 3396.0 | 2607.0 | 12323.0 | 20403.0 | 6929.0 | 4400.0 | 16568.0 | 0.0 | 4457.0 | 3476.0 | 2971.0 | 0.0 | 4775.0 | 6217.0 | 5539.0 | 1978.0 | 11644.0 | 9480.0 | 0.0 | 2497.0 | 0.0 | 2115.0 | 2062.0 | 1677.0 | 2515.0 | 0.0 | 9305.0 | 10468.0 | 3725.0 | 2655.0 | 1830.0 | 1184.0 | 12924.0 | 12025.0 | 0.0 | 0.0 | 3441.0 | 3441.0 | 21771.0 | 2842.0 | 0.0 | 7944.0 | 12832.0 | 3704.0 | 0.0 | 2192.0 | 0.0 | 0.0 | 0.0 | 0.0 | 0.0 | 3024.0 | 0.0 | 0.0 | 0.0 | 0.0 | 0.0 | 0.0 | 0.0 | 0.0 | 0.0 | 0.0 | 0.0 | 0.0 | 0.0 | 0.0 | 0.0 | 0.0 | 0.0 | 0.0 | 0.0 | 1836.0 | 1799.0 | 0.0 | 0.0 | 0.0 | 0.0 | 0.0 | 1954.0 | 1813.0 | 0.0 | 0.0 | 1574.0 | 0.0 | 0.0 | 0.0 |
| 394 | 20094.0 | 13157.0 | 20172.0 | 13955.0 | 2406.0 | 3433.0 | 20396.0 | 10856.0 | 12911.0 | 10944.0 | 3203.0 | 9704.0 | 2673.0 | 24629.0 | 3212.0 | 2308.0 | 12088.0 | 23969.0 | 6964.0 | 5157.0 | 16361.0 | 0.0 | 4250.0 | 4128.0 | 3375.0 | 0.0 | 4942.0 | 5993.0 | 6152.0 | 1893.0 | 11121.0 | 8883.0 | 0.0 | 1922.0 | 0.0 | 2014.0 | 1974.0 | 1613.0 | 2520.0 | 0.0 | 8743.0 | 9696.0 | 3629.0 | 2540.0 | 1773.0 | 0.0 | 13797.0 | 11665.0 | 1073.0 | 0.0 | 3527.0 | 3439.0 | 22322.0 | 2851.0 | 0.0 | 8199.0 | 12371.0 | 4015.0 | 0.0 | 2086.0 | 0.0 | 0.0 | 0.0 | 0.0 | 0.0 | 3136.0 | 0.0 | 0.0 | 0.0 | 0.0 | 0.0 | 0.0 | 0.0 | 0.0 | 0.0 | 0.0 | 0.0 | 0.0 | 0.0 | 0.0 | 0.0 | 0.0 | 0.0 | 0.0 | 0.0 | 1723.0 | 1551.0 | 0.0 | 0.0 | 0.0 | 0.0 | 0.0 | 1725.0 | 1555.0 | 0.0 | 0.0 | 1300.0 | 0.0 | 0.0 | 0.0 |
| 399 | 12614.0 | 12314.0 | 16924.0 | 12016.0 | 2181.0 | 3043.0 | 18880.0 | 9990.0 | 12077.0 | 10284.0 | 2806.0 | 9319.0 | 2524.0 | 20656.0 | 2964.0 | 2099.0 | 11352.0 | 18417.0 | 6091.0 | 3712.0 | 14743.0 | 0.0 | 3743.0 | 2886.0 | 2350.0 | 0.0 | 3989.0 | 5283.0 | 4928.0 | 1697.0 | 9953.0 | 8040.0 | 0.0 | 1980.0 | 0.0 | 1675.0 | 1769.0 | 1380.0 | 2112.0 | 0.0 | 8179.0 | 8763.0 | 2998.0 | 2202.0 | 1446.0 | 0.0 | 11038.0 | 10353.0 | 0.0 | 0.0 | 3022.0 | 2916.0 | 19869.0 | 2373.0 | 0.0 | 7060.0 | 11443.0 | 3213.0 | 0.0 | 0.0 | 0.0 | 0.0 | 0.0 | 0.0 | 0.0 | 2722.0 | 0.0 | 0.0 | 0.0 | 0.0 | 0.0 | 0.0 | 0.0 | 0.0 | 0.0 | 0.0 | 0.0 | 0.0 | 0.0 | 0.0 | 0.0 | 0.0 | 0.0 | 0.0 | 0.0 | 1601.0 | 1634.0 | 0.0 | 0.0 | 0.0 | 0.0 | 0.0 | 1772.0 | 1602.0 | 0.0 | 0.0 | 1367.0 | 0.0 | 0.0 | 0.0 |
| 406 | 13837.0 | 12471.0 | 16607.0 | 11953.0 | 2465.0 | 3229.0 | 18894.0 | 10560.0 | 12105.0 | 10522.0 | 3110.0 | 9293.0 | 2661.0 | 22089.0 | 3155.0 | 2232.0 | 11094.0 | 20166.0 | 6410.0 | 3798.0 | 16402.0 | 0.0 | 3920.0 | 3134.0 | 2500.0 | 0.0 | 4349.0 | 5892.0 | 5465.0 | 1757.0 | 11664.0 | 9387.0 | 0.0 | 1477.0 | 0.0 | 1779.0 | 1840.0 | 1443.0 | 2332.0 | 0.0 | 9287.0 | 10223.0 | 3391.0 | 2484.0 | 1449.0 | 0.0 | 12233.0 | 11853.0 | 0.0 | 0.0 | 3213.0 | 3077.0 | 20712.0 | 2673.0 | 0.0 | 7459.0 | 12566.0 | 3482.0 | 0.0 | 0.0 | 0.0 | 0.0 | 0.0 | 0.0 | 0.0 | 2725.0 | 0.0 | 0.0 | 0.0 | 0.0 | 0.0 | 0.0 | 0.0 | 0.0 | 0.0 | 0.0 | 0.0 | 0.0 | 0.0 | 0.0 | 0.0 | 0.0 | 0.0 | 0.0 | 0.0 | 1491.0 | 1333.0 | 0.0 | 0.0 | 0.0 | 0.0 | 0.0 | 1943.0 | 1754.0 | 0.0 | 0.0 | 1358.0 | 0.0 | 0.0 | 0.0 |
| 412 | 10681.0 | 13025.0 | 16078.0 | 11028.0 | 2384.0 | 3197.0 | 18615.0 | 10794.0 | 12476.0 | 10579.0 | 2984.0 | 9459.0 | 2429.0 | 20115.0 | 3035.0 | 2174.0 | 11570.0 | 17115.0 | 6375.0 | 3141.0 | 16381.0 | 0.0 | 3887.0 | 2563.0 | 1998.0 | 0.0 | 4034.0 | 5719.0 | 5237.0 | 1626.0 | 11298.0 | 8997.0 | 0.0 | 1750.0 | 0.0 | 1593.0 | 1659.0 | 1296.0 | 2237.0 | 0.0 | 9122.0 | 9949.0 | 3255.0 | 2415.0 | 1345.0 | 0.0 | 11672.0 | 11805.0 | 0.0 | 0.0 | 2940.0 | 2954.0 | 19770.0 | 2516.0 | 0.0 | 7544.0 | 12974.0 | 3356.0 | 0.0 | 1895.0 | 0.0 | 0.0 | 0.0 | 0.0 | 0.0 | 2736.0 | 0.0 | 0.0 | 0.0 | 0.0 | 0.0 | 0.0 | 0.0 | 0.0 | 0.0 | 0.0 | 0.0 | 0.0 | 0.0 | 0.0 | 0.0 | 0.0 | 0.0 | 0.0 | 0.0 | 1620.0 | 1675.0 | 0.0 | 0.0 | 0.0 | 0.0 | 0.0 | 2230.0 | 1972.0 | 0.0 | 0.0 | 1465.0 | 0.0 | 0.0 | 0.0 |
| 413 | 11769.0 | 12461.0 | 16456.0 | 11309.0 | 2188.0 | 2934.0 | 18867.0 | 10182.0 | 11784.0 | 10166.0 | 2874.0 | 8976.0 | 2357.0 | 21303.0 | 2850.0 | 2017.0 | 10572.0 | 18808.0 | 6163.0 | 3150.0 | 16416.0 | 0.0 | 3507.0 | 2527.0 | 2020.0 | 0.0 | 3852.0 | 5637.0 | 5496.0 | 1597.0 | 11305.0 | 9388.0 | 0.0 | 1809.0 | 0.0 | 1508.0 | 1538.0 | 1178.0 | 2051.0 | 0.0 | 9114.0 | 9886.0 | 3002.0 | 2161.0 | 1276.0 | 0.0 | 11606.0 | 11457.0 | 0.0 | 0.0 | 2721.0 | 2804.0 | 20537.0 | 2391.0 | 0.0 | 6927.0 | 12836.0 | 3476.0 | 0.0 | 0.0 | 0.0 | 0.0 | 0.0 | 0.0 | 0.0 | 2458.0 | 0.0 | 0.0 | 0.0 | 0.0 | 0.0 | 0.0 | 0.0 | 0.0 | 0.0 | 0.0 | 0.0 | 0.0 | 0.0 | 0.0 | 0.0 | 0.0 | 0.0 | 0.0 | 0.0 | 1658.0 | 1703.0 | 0.0 | 0.0 | 0.0 | 0.0 | 0.0 | 2447.0 | 2186.0 | 0.0 | 0.0 | 1519.0 | 0.0 | 0.0 | 0.0 |
| 429 | 24625.0 | 8066.0 | 13584.0 | 8681.0 | 1936.0 | 2511.0 | 16094.0 | 6532.0 | 9045.0 | 7481.0 | 1560.0 | 6320.0 | 2094.0 | 20503.0 | 2484.0 | 2040.0 | 7633.0 | 20394.0 | 4216.0 | 2130.0 | 10812.0 | 0.0 | 2477.0 | 1580.0 | 1366.0 | 0.0 | 2874.0 | 4010.0 | 3849.0 | 1382.0 | 6749.0 | 5303.0 | 0.0 | 0.0 | 0.0 | 1372.0 | 1502.0 | 1169.0 | 1314.0 | 0.0 | 5724.0 | 6033.0 | 1687.0 | 1370.0 | 0.0 | 0.0 | 7938.0 | 7173.0 | 0.0 | 0.0 | 2141.0 | 1687.0 | 19075.0 | 1264.0 | 0.0 | 4980.0 | 8149.0 | 2920.0 | 0.0 | 0.0 | 0.0 | 0.0 | 0.0 | 0.0 | 0.0 | 2065.0 | 0.0 | 0.0 | 0.0 | 0.0 | 0.0 | 0.0 | 0.0 | 0.0 | 0.0 | 0.0 | 0.0 | 0.0 | 0.0 | 0.0 | 0.0 | 0.0 | 0.0 | 0.0 | 0.0 | 1769.0 | 1779.0 | 0.0 | 0.0 | 0.0 | 0.0 | 0.0 | 1664.0 | 1575.0 | 1290.0 | 0.0 | 0.0 | 0.0 | 0.0 | 0.0 |
| 432 | 25181.0 | 6038.0 | 11814.0 | 7215.0 | 1214.0 | 1656.0 | 13333.0 | 4559.0 | 7008.0 | 5629.0 | 0.0 | 4707.0 | 1316.0 | 17863.0 | 1679.0 | 1287.0 | 5798.0 | 18321.0 | 3089.0 | 1676.0 | 8442.0 | 0.0 | 1605.0 | 1369.0 | 1115.0 | 0.0 | 2082.0 | 3013.0 | 2918.0 | 0.0 | 5322.0 | 4095.0 | 0.0 | 0.0 | 0.0 | 0.0 | 0.0 | 0.0 | 0.0 | 0.0 | 4062.0 | 4454.0 | 1106.0 | 0.0 | 0.0 | 0.0 | 6584.0 | 5409.0 | 0.0 | 0.0 | 1558.0 | 1427.0 | 16678.0 | 0.0 | 0.0 | 3623.0 | 6327.0 | 2040.0 | 0.0 | 0.0 | 0.0 | 0.0 | 0.0 | 0.0 | 0.0 | 0.0 | 0.0 | 0.0 | 0.0 | 0.0 | 0.0 | 0.0 | 0.0 | 0.0 | 0.0 | 0.0 | 0.0 | 0.0 | 0.0 | 0.0 | 0.0 | 0.0 | 0.0 | 0.0 | 0.0 | 2162.0 | 2027.0 | 0.0 | 0.0 | 0.0 | 0.0 | 0.0 | 1933.0 | 1712.0 | 1338.0 | 0.0 | 1430.0 | 0.0 | 0.0 | 0.0 |
| 440 | 21421.0 | 7200.0 | 13772.0 | 8264.0 | 1193.0 | 1742.0 | 15514.0 | 5578.0 | 8134.0 | 6545.0 | 1207.0 | 5640.0 | 1355.0 | 20462.0 | 1668.0 | 1418.0 | 6857.0 | 20408.0 | 3640.0 | 1745.0 | 9895.0 | 0.0 | 2010.0 | 1267.0 | 1105.0 | 0.0 | 2268.0 | 3515.0 | 3476.0 | 0.0 | 6029.0 | 4346.0 | 0.0 | 0.0 | 0.0 | 0.0 | 0.0 | 0.0 | 0.0 | 0.0 | 4433.0 | 4978.0 | 1343.0 | 0.0 | 0.0 | 0.0 | 6854.0 | 5895.0 | 0.0 | 0.0 | 1646.0 | 1540.0 | 19324.0 | 0.0 | 0.0 | 3945.0 | 6663.0 | 2527.0 | 0.0 | 0.0 | 0.0 | 0.0 | 0.0 | 0.0 | 0.0 | 1551.0 | 0.0 | 0.0 | 0.0 | 0.0 | 0.0 | 0.0 | 0.0 | 0.0 | 0.0 | 0.0 | 0.0 | 0.0 | 0.0 | 0.0 | 0.0 | 0.0 | 0.0 | 0.0 | 0.0 | 2097.0 | 2144.0 | 0.0 | 0.0 | 0.0 | 0.0 | 0.0 | 1899.0 | 1664.0 | 0.0 | 0.0 | 0.0 | 0.0 | 0.0 | 0.0 |
| 446 | 20414.0 | 7746.0 | 14409.0 | 8923.0 | 1232.0 | 1744.0 | 16776.0 | 6251.0 | 8642.0 | 6785.0 | 1270.0 | 6139.0 | 1320.0 | 21498.0 | 1679.0 | 1373.0 | 7223.0 | 21709.0 | 4033.0 | 1761.0 | 10702.0 | 0.0 | 2133.0 | 1249.0 | 1122.0 | 0.0 | 2347.0 | 3707.0 | 3602.0 | 0.0 | 6828.0 | 4868.0 | 0.0 | 0.0 | 0.0 | 0.0 | 0.0 | 0.0 | 0.0 | 0.0 | 5124.0 | 5419.0 | 1430.0 | 0.0 | 0.0 | 0.0 | 7185.0 | 6915.0 | 0.0 | 0.0 | 1679.0 | 1655.0 | 20109.0 | 0.0 | 0.0 | 4101.0 | 7519.0 | 2545.0 | 0.0 | 0.0 | 0.0 | 0.0 | 0.0 | 0.0 | 0.0 | 0.0 | 0.0 | 0.0 | 0.0 | 0.0 | 0.0 | 0.0 | 0.0 | 0.0 | 0.0 | 0.0 | 0.0 | 0.0 | 0.0 | 0.0 | 0.0 | 0.0 | 0.0 | 0.0 | 0.0 | 2654.0 | 2763.0 | 0.0 | 0.0 | 0.0 | 0.0 | 0.0 | 2274.0 | 1977.0 | 1216.0 | 0.0 | 1442.0 | 0.0 | 0.0 | 0.0 |
| 460 | 13111.0 | 6618.0 | 12230.0 | 7553.0 | 0.0 | 1396.0 | 14061.0 | 5046.0 | 7315.0 | 5772.0 | 0.0 | 5024.0 | 0.0 | 17622.0 | 1373.0 | 0.0 | 5990.0 | 17064.0 | 3312.0 | 1419.0 | 9333.0 | 0.0 | 1799.0 | 1017.0 | 0.0 | 0.0 | 1936.0 | 3149.0 | 3189.0 | 0.0 | 5685.0 | 4115.0 | 0.0 | 0.0 | 0.0 | 0.0 | 0.0 | 0.0 | 0.0 | 0.0 | 4166.0 | 4589.0 | 1135.0 | 0.0 | 0.0 | 0.0 | 6043.0 | 5762.0 | 0.0 | 0.0 | 1440.0 | 1257.0 | 17311.0 | 0.0 | 0.0 | 3386.0 | 6329.0 | 2194.0 | 0.0 | 0.0 | 0.0 | 0.0 | 0.0 | 0.0 | 0.0 | 1284.0 | 0.0 | 0.0 | 0.0 | 0.0 | 0.0 | 0.0 | 0.0 | 0.0 | 0.0 | 0.0 | 0.0 | 0.0 | 0.0 | 0.0 | 0.0 | 0.0 | 0.0 | 0.0 | 0.0 | 2102.0 | 2174.0 | 0.0 | 0.0 | 0.0 | 0.0 | 0.0 | 1804.0 | 1548.0 | 0.0 | 0.0 | 0.0 | 0.0 | 0.0 | 0.0 |
| 468 | 19765.0 | 7574.0 | 13903.0 | 8358.0 | 0.0 | 1433.0 | 16462.0 | 5694.0 | 8265.0 | 6583.0 | 1184.0 | 5841.0 | 1016.0 | 21133.0 | 1372.0 | 0.0 | 7277.0 | 21435.0 | 3747.0 | 1491.0 | 10414.0 | 0.0 | 2044.0 | 0.0 | 0.0 | 0.0 | 2119.0 | 3553.0 | 3699.0 | 0.0 | 6314.0 | 4499.0 | 0.0 | 0.0 | 0.0 | 0.0 | 0.0 | 0.0 | 0.0 | 0.0 | 4678.0 | 5042.0 | 1190.0 | 0.0 | 0.0 | 0.0 | 6815.0 | 6152.0 | 0.0 | 0.0 | 1477.0 | 1099.0 | 19758.0 | 0.0 | 0.0 | 3683.0 | 6780.0 | 2278.0 | 0.0 | 0.0 | 0.0 | 0.0 | 0.0 | 0.0 | 0.0 | 1463.0 | 0.0 | 0.0 | 0.0 | 0.0 | 0.0 | 0.0 | 0.0 | 0.0 | 0.0 | 0.0 | 0.0 | 0.0 | 0.0 | 0.0 | 0.0 | 0.0 | 0.0 | 0.0 | 0.0 | 1734.0 | 1248.0 | 0.0 | 0.0 | 0.0 | 0.0 | 0.0 | 1428.0 | 1295.0 | 0.0 | 0.0 | 0.0 | 0.0 | 0.0 | 0.0 |
| 474 | 14560.0 | 8332.0 | 15320.0 | 9781.0 | 1237.0 | 2093.0 | 16374.0 | 6664.0 | 9625.0 | 7595.0 | 1545.0 | 6856.0 | 1406.0 | 20670.0 | 2222.0 | 1353.0 | 8003.0 | 19557.0 | 4587.0 | 2567.0 | 11080.0 | 0.0 | 2748.0 | 2026.0 | 1800.0 | 0.0 | 3020.0 | 4593.0 | 4938.0 | 0.0 | 7214.0 | 5247.0 | 0.0 | 0.0 | 0.0 | 1227.0 | 1252.0 | 0.0 | 0.0 | 0.0 | 5506.0 | 5940.0 | 1510.0 | 0.0 | 0.0 | 0.0 | 8326.0 | 7060.0 | 0.0 | 0.0 | 2186.0 | 1539.0 | 19105.0 | 0.0 | 0.0 | 4852.0 | 7827.0 | 3241.0 | 0.0 | 0.0 | 0.0 | 0.0 | 0.0 | 0.0 | 0.0 | 0.0 | 0.0 | 0.0 | 0.0 | 0.0 | 0.0 | 0.0 | 0.0 | 0.0 | 0.0 | 0.0 | 0.0 | 0.0 | 0.0 | 0.0 | 0.0 | 0.0 | 0.0 | 0.0 | 0.0 | 2204.0 | 1639.0 | 0.0 | 0.0 | 0.0 | 0.0 | 0.0 | 1591.0 | 1360.0 | 0.0 | 0.0 | 0.0 | 0.0 | 0.0 | 0.0 |
| 503 | 8775.0 | 9487.0 | 15990.0 | 9684.0 | 1372.0 | 2832.0 | 17977.0 | 7724.0 | 11122.0 | 8783.0 | 1694.0 | 7758.0 | 1663.0 | 19813.0 | 2941.0 | 1537.0 | 9641.0 | 17639.0 | 5902.0 | 3611.0 | 14656.0 | 0.0 | 3565.0 | 2947.0 | 2728.0 | 0.0 | 3968.0 | 5654.0 | 5773.0 | 1490.0 | 9470.0 | 7502.0 | 0.0 | 0.0 | 0.0 | 1479.0 | 1538.0 | 1302.0 | 0.0 | 0.0 | 7521.0 | 8181.0 | 1583.0 | 0.0 | 1362.0 | 0.0 | 10819.0 | 9783.0 | 0.0 | 0.0 | 3188.0 | 1866.0 | 19720.0 | 1155.0 | 0.0 | 6724.0 | 10662.0 | 3737.0 | 0.0 | 0.0 | 0.0 | 0.0 | 0.0 | 0.0 | 0.0 | 2184.0 | 0.0 | 0.0 | 0.0 | 0.0 | 0.0 | 0.0 | 0.0 | 0.0 | 0.0 | 0.0 | 0.0 | 0.0 | 0.0 | 0.0 | 0.0 | 0.0 | 0.0 | 0.0 | 0.0 | 3453.0 | 3338.0 | 0.0 | 0.0 | 0.0 | 0.0 | 0.0 | 3246.0 | 2992.0 | 1438.0 | 0.0 | 1815.0 | 0.0 | 0.0 | 0.0 |
| 517 | 22469.0 | 4275.0 | 8232.0 | 4762.0 | 0.0 | 1404.0 | 10480.0 | 3249.0 | 5310.0 | 4178.0 | 0.0 | 3598.0 | 0.0 | 14214.0 | 1407.0 | 1196.0 | 3999.0 | 13964.0 | 2749.0 | 1467.0 | 7488.0 | 0.0 | 1718.0 | 0.0 | 0.0 | 0.0 | 2263.0 | 3313.0 | 3413.0 | 1321.0 | 4550.0 | 3762.0 | 0.0 | 0.0 | 0.0 | 1185.0 | 1368.0 | 1132.0 | 0.0 | 0.0 | 3897.0 | 4045.0 | 0.0 | 0.0 | 0.0 | 0.0 | 5530.0 | 5406.0 | 0.0 | 0.0 | 1732.0 | 1211.0 | 13847.0 | 0.0 | 0.0 | 3343.0 | 5767.0 | 2645.0 | 0.0 | 0.0 | 0.0 | 0.0 | 0.0 | 0.0 | 0.0 | 0.0 | 0.0 | 0.0 | 0.0 | 0.0 | 0.0 | 0.0 | 0.0 | 0.0 | 0.0 | 0.0 | 0.0 | 0.0 | 0.0 | 0.0 | 0.0 | 0.0 | 0.0 | 0.0 | 0.0 | 1688.0 | 1918.0 | 0.0 | 0.0 | 0.0 | 0.0 | 0.0 | 1981.0 | 1883.0 | 1663.0 | 0.0 | 1752.0 | 0.0 | 0.0 | 0.0 |
| 528 | 18792.0 | 12775.0 | 14392.0 | 11441.0 | 1540.0 | 4164.0 | 20285.0 | 9719.0 | 12944.0 | 9779.0 | 1677.0 | 8850.0 | 1810.0 | 23180.0 | 3418.0 | 1509.0 | 10510.0 | 22336.0 | 2995.0 | 1388.0 | 8033.0 | 0.0 | 1740.0 | 0.0 | 0.0 | 0.0 | 1907.0 | 3436.0 | 3735.0 | 0.0 | 4805.0 | 3461.0 | 0.0 | 0.0 | 0.0 | 0.0 | 0.0 | 0.0 | 0.0 | 0.0 | 3728.0 | 3920.0 | 0.0 | 0.0 | 0.0 | 0.0 | 5658.0 | 5039.0 | 0.0 | 0.0 | 1489.0 | 0.0 | 17817.0 | 0.0 | 0.0 | 3333.0 | 5557.0 | 2619.0 | 0.0 | 0.0 | 0.0 | 0.0 | 0.0 | 0.0 | 0.0 | 0.0 | 0.0 | 0.0 | 0.0 | 0.0 | 0.0 | 0.0 | 0.0 | 0.0 | 0.0 | 0.0 | 0.0 | 0.0 | 0.0 | 0.0 | 0.0 | 0.0 | 0.0 | 0.0 | 0.0 | 2113.0 | 2185.0 | 0.0 | 0.0 | 0.0 | 0.0 | 0.0 | 3296.0 | 2995.0 | 1261.0 | 0.0 | 2064.0 | 0.0 | 0.0 | 0.0 |
| 538 | 19487.0 | 13747.0 | 17852.0 | 12885.0 | 1563.0 | 4751.0 | 22358.0 | 10898.0 | 15010.0 | 11169.0 | 1764.0 | 10023.0 | 2006.0 | 25020.0 | 3986.0 | 1575.0 | 11869.0 | 23875.0 | 3219.0 | 1721.0 | 8369.0 | 0.0 | 1838.0 | 1425.0 | 1192.0 | 0.0 | 1981.0 | 3993.0 | 4821.0 | 0.0 | 4977.0 | 3503.0 | 0.0 | 0.0 | 0.0 | 0.0 | 0.0 | 0.0 | 0.0 | 0.0 | 3805.0 | 3962.0 | 0.0 | 0.0 | 0.0 | 0.0 | 6145.0 | 5037.0 | 0.0 | 0.0 | 1605.0 | 0.0 | 19914.0 | 0.0 | 0.0 | 3400.0 | 5429.0 | 3152.0 | 0.0 | 0.0 | 0.0 | 0.0 | 0.0 | 0.0 | 0.0 | 1382.0 | 0.0 | 0.0 | 0.0 | 0.0 | 0.0 | 0.0 | 0.0 | 0.0 | 0.0 | 0.0 | 0.0 | 0.0 | 0.0 | 0.0 | 0.0 | 0.0 | 0.0 | 0.0 | 0.0 | 2200.0 | 2188.0 | 0.0 | 0.0 | 0.0 | 0.0 | 0.0 | 3326.0 | 2943.0 | 0.0 | 0.0 | 1990.0 | 0.0 | 0.0 | 0.0 |
| 542 | 21026.0 | 12079.0 | 16086.0 | 11786.0 | 1331.0 | 3692.0 | 20842.0 | 8879.0 | 13235.0 | 9692.0 | 1533.0 | 8394.0 | 1666.0 | 24400.0 | 3135.0 | 1417.0 | 9700.0 | 23723.0 | 4393.0 | 1408.0 | 7280.0 | 0.0 | 1243.0 | 1232.0 | 0.0 | 0.0 | 1781.0 | 3546.0 | 4419.0 | 0.0 | 4213.0 | 2882.0 | 0.0 | 0.0 | 0.0 | 0.0 | 0.0 | 0.0 | 0.0 | 0.0 | 3162.0 | 3472.0 | 0.0 | 0.0 | 0.0 | 0.0 | 5205.0 | 4323.0 | 0.0 | 0.0 | 1349.0 | 0.0 | 18129.0 | 0.0 | 0.0 | 2859.0 | 4435.0 | 2870.0 | 0.0 | 0.0 | 0.0 | 0.0 | 0.0 | 0.0 | 0.0 | 1189.0 | 0.0 | 0.0 | 0.0 | 0.0 | 0.0 | 0.0 | 0.0 | 0.0 | 0.0 | 0.0 | 0.0 | 0.0 | 0.0 | 0.0 | 0.0 | 0.0 | 0.0 | 0.0 | 0.0 | 2179.0 | 2136.0 | 0.0 | 0.0 | 1121.0 | 0.0 | 0.0 | 3045.0 | 2676.0 | 0.0 | 0.0 | 1674.0 | 0.0 | 0.0 | 0.0 |
| 556 | 12951.0 | 12986.0 | 15620.0 | 11446.0 | 1325.0 | 3955.0 | 20414.0 | 10042.0 | 13463.0 | 10431.0 | 1608.0 | 9528.0 | 1677.0 | 21603.0 | 3249.0 | 1291.0 | 11644.0 | 18451.0 | 3016.0 | 1276.0 | 7697.0 | 0.0 | 1782.0 | 0.0 | 0.0 | 0.0 | 1618.0 | 3359.0 | 3748.0 | 0.0 | 4668.0 | 2972.0 | 0.0 | 0.0 | 0.0 | 0.0 | 0.0 | 0.0 | 0.0 | 0.0 | 3290.0 | 3411.0 | 0.0 | 0.0 | 0.0 | 0.0 | 5190.0 | 4347.0 | 0.0 | 0.0 | 1211.0 | 0.0 | 19086.0 | 0.0 | 0.0 | 2944.0 | 4742.0 | 2327.0 | 0.0 | 0.0 | 0.0 | 0.0 | 0.0 | 0.0 | 0.0 | 0.0 | 0.0 | 0.0 | 0.0 | 0.0 | 0.0 | 0.0 | 0.0 | 0.0 | 0.0 | 0.0 | 0.0 | 0.0 | 0.0 | 0.0 | 0.0 | 0.0 | 0.0 | 0.0 | 0.0 | 2165.0 | 2152.0 | 0.0 | 0.0 | 1028.0 | 0.0 | 0.0 | 2840.0 | 2471.0 | 0.0 | 0.0 | 1625.0 | 0.0 | 0.0 | 0.0 |
| 566 | 15975.0 | 12713.0 | 15856.0 | 11353.0 | 1372.0 | 4033.0 | 21250.0 | 10253.0 | 13950.0 | 10683.0 | 1657.0 | 9475.0 | 1786.0 | 22341.0 | 3303.0 | 1390.0 | 11326.0 | 20810.0 | 3250.0 | 1606.0 | 8267.0 | 0.0 | 1913.0 | 1319.0 | 0.0 | 0.0 | 1765.0 | 3671.0 | 4309.0 | 0.0 | 5257.0 | 3462.0 | 0.0 | 0.0 | 0.0 | 0.0 | 0.0 | 0.0 | 0.0 | 0.0 | 3481.0 | 3874.0 | 0.0 | 0.0 | 0.0 | 0.0 | 5758.0 | 4710.0 | 0.0 | 0.0 | 1371.0 | 0.0 | 19746.0 | 0.0 | 0.0 | 3133.0 | 5129.0 | 2748.0 | 0.0 | 0.0 | 0.0 | 0.0 | 0.0 | 0.0 | 0.0 | 0.0 | 0.0 | 0.0 | 0.0 | 0.0 | 0.0 | 0.0 | 0.0 | 0.0 | 0.0 | 0.0 | 0.0 | 0.0 | 0.0 | 0.0 | 0.0 | 0.0 | 0.0 | 0.0 | 0.0 | 2317.0 | 2250.0 | 0.0 | 0.0 | 1082.0 | 0.0 | 0.0 | 3040.0 | 2624.0 | 0.0 | 0.0 | 1615.0 | 0.0 | 0.0 | 0.0 |
| 569 | 12469.0 | 13831.0 | 15845.0 | 11522.0 | 1525.0 | 4216.0 | 18568.0 | 10721.0 | 14087.0 | 11488.0 | 1890.0 | 10029.0 | 1962.0 | 19296.0 | 3684.0 | 1433.0 | 11673.0 | 16847.0 | 3677.0 | 1472.0 | 9141.0 | 0.0 | 2080.0 | 1170.0 | 0.0 | 0.0 | 1830.0 | 3922.0 | 4204.0 | 0.0 | 5624.0 | 3708.0 | 0.0 | 0.0 | 0.0 | 0.0 | 0.0 | 0.0 | 0.0 | 0.0 | 4030.0 | 4257.0 | 0.0 | 0.0 | 0.0 | 0.0 | 5894.0 | 5178.0 | 0.0 | 0.0 | 1461.0 | 0.0 | 19951.0 | 0.0 | 0.0 | 3421.0 | 5721.0 | 2710.0 | 0.0 | 0.0 | 0.0 | 0.0 | 0.0 | 0.0 | 0.0 | 0.0 | 0.0 | 0.0 | 0.0 | 0.0 | 0.0 | 0.0 | 0.0 | 0.0 | 0.0 | 0.0 | 0.0 | 0.0 | 0.0 | 0.0 | 0.0 | 0.0 | 0.0 | 0.0 | 0.0 | 2616.0 | 2524.0 | 0.0 | 0.0 | 1119.0 | 0.0 | 0.0 | 3412.0 | 2933.0 | 0.0 | 0.0 | 1754.0 | 0.0 | 0.0 | 0.0 |
| 584 | 17064.0 | 15158.0 | 19090.0 | 13436.0 | 1525.0 | 4154.0 | 22960.0 | 11595.0 | 15739.0 | 12638.0 | 1933.0 | 11224.0 | 1878.0 | 24217.0 | 3634.0 | 1415.0 | 13651.0 | 21993.0 | 4218.0 | 1554.0 | 10564.0 | 0.0 | 2393.0 | 1139.0 | 0.0 | 0.0 | 2044.0 | 4456.0 | 4626.0 | 0.0 | 6400.0 | 3994.0 | 0.0 | 0.0 | 0.0 | 0.0 | 0.0 | 0.0 | 0.0 | 0.0 | 4474.0 | 4642.0 | 0.0 | 0.0 | 0.0 | 0.0 | 6965.0 | 5817.0 | 0.0 | 0.0 | 1481.0 | 0.0 | 22996.0 | 0.0 | 0.0 | 3999.0 | 6404.0 | 2856.0 | 0.0 | 0.0 | 0.0 | 0.0 | 0.0 | 0.0 | 0.0 | 0.0 | 0.0 | 0.0 | 0.0 | 0.0 | 0.0 | 0.0 | 0.0 | 0.0 | 0.0 | 0.0 | 0.0 | 0.0 | 0.0 | 0.0 | 0.0 | 0.0 | 0.0 | 0.0 | 0.0 | 2892.0 | 2768.0 | 0.0 | 0.0 | 0.0 | 0.0 | 0.0 | 2854.0 | 2505.0 | 0.0 | 0.0 | 1555.0 | 0.0 | 0.0 | 0.0 |
| 592 | 17002.0 | 16965.0 | 21217.0 | 14694.0 | 1883.0 | 5145.0 | 23418.0 | 12969.0 | 16607.0 | 13984.0 | 2312.0 | 12192.0 | 2476.0 | 23800.0 | 4658.0 | 1918.0 | 15147.0 | 21598.0 | 5014.0 | 2348.0 | 12102.0 | 0.0 | 3257.0 | 1909.0 | 1646.0 | 0.0 | 2730.0 | 5588.0 | 6463.0 | 0.0 | 7687.0 | 4864.0 | 0.0 | 0.0 | 0.0 | 0.0 | 0.0 | 0.0 | 0.0 | 0.0 | 5379.0 | 5574.0 | 0.0 | 0.0 | 0.0 | 0.0 | 8056.0 | 6766.0 | 0.0 | 0.0 | 2009.0 | 0.0 | 23848.0 | 0.0 | 0.0 | 4754.0 | 7520.0 | 3872.0 | 0.0 | 0.0 | 0.0 | 0.0 | 0.0 | 0.0 | 0.0 | 0.0 | 0.0 | 0.0 | 0.0 | 0.0 | 0.0 | 0.0 | 0.0 | 0.0 | 0.0 | 0.0 | 0.0 | 0.0 | 0.0 | 0.0 | 0.0 | 0.0 | 0.0 | 0.0 | 0.0 | 3459.0 | 3294.0 | 0.0 | 0.0 | 0.0 | 0.0 | 0.0 | 3491.0 | 3129.0 | 0.0 | 0.0 | 1846.0 | 0.0 | 0.0 | 0.0 |
| 598 | 17498.0 | 17132.0 | 21111.0 | 14396.0 | 1891.0 | 5382.0 | 23202.0 | 13721.0 | 16980.0 | 14403.0 | 2413.0 | 12957.0 | 2518.0 | 23463.0 | 4803.0 | 1943.0 | 15390.0 | 21646.0 | 5462.0 | 2381.0 | 12404.0 | 0.0 | 3365.0 | 1974.0 | 1603.0 | 0.0 | 2913.0 | 5768.0 | 6739.0 | 0.0 | 7900.0 | 5116.0 | 0.0 | 1356.0 | 0.0 | 0.0 | 1107.0 | 0.0 | 0.0 | 0.0 | 5667.0 | 5767.0 | 1089.0 | 0.0 | 0.0 | 0.0 | 8605.0 | 7284.0 | 0.0 | 0.0 | 2168.0 | 1348.0 | 24432.0 | 0.0 | 0.0 | 5018.0 | 8038.0 | 3894.0 | 0.0 | 0.0 | 0.0 | 0.0 | 0.0 | 0.0 | 0.0 | 0.0 | 0.0 | 0.0 | 0.0 | 0.0 | 0.0 | 0.0 | 0.0 | 0.0 | 0.0 | 0.0 | 0.0 | 0.0 | 0.0 | 0.0 | 0.0 | 0.0 | 0.0 | 0.0 | 0.0 | 3213.0 | 3199.0 | 0.0 | 0.0 | 0.0 | 0.0 | 0.0 | 3190.0 | 2800.0 | 0.0 | 0.0 | 1659.0 | 0.0 | 0.0 | 0.0 |
| 612 | 14173.0 | 14813.0 | 19364.0 | 13442.0 | 1556.0 | 4032.0 | 22812.0 | 11123.0 | 15708.0 | 12286.0 | 1933.0 | 10962.0 | 1960.0 | 23779.0 | 3665.0 | 1536.0 | 13031.0 | 21468.0 | 4722.0 | 1697.0 | 11177.0 | 0.0 | 2963.0 | 1302.0 | 1084.0 | 0.0 | 2189.0 | 5187.0 | 5909.0 | 0.0 | 6900.0 | 4110.0 | 0.0 | 0.0 | 0.0 | 0.0 | 0.0 | 0.0 | 0.0 | 0.0 | 4514.0 | 4884.0 | 0.0 | 0.0 | 0.0 | 0.0 | 6882.0 | 6075.0 | 0.0 | 0.0 | 1699.0 | 1115.0 | 23558.0 | 0.0 | 0.0 | 3916.0 | 6591.0 | 3544.0 | 0.0 | 0.0 | 0.0 | 0.0 | 0.0 | 0.0 | 0.0 | 0.0 | 0.0 | 0.0 | 0.0 | 0.0 | 0.0 | 0.0 | 0.0 | 0.0 | 0.0 | 0.0 | 0.0 | 0.0 | 0.0 | 0.0 | 0.0 | 0.0 | 0.0 | 0.0 | 0.0 | 3015.0 | 2836.0 | 0.0 | 0.0 | 0.0 | 0.0 | 0.0 | 3042.0 | 2674.0 | 0.0 | 0.0 | 1632.0 | 0.0 | 0.0 | 0.0 |
| 622 | 11077.0 | 16360.0 | 20120.0 | 13552.0 | 1708.0 | 4394.0 | 22605.0 | 12625.0 | 16533.0 | 13912.0 | 2079.0 | 11871.0 | 2108.0 | 22659.0 | 3896.0 | 1582.0 | 14651.0 | 18536.0 | 4862.0 | 1890.0 | 11900.0 | 0.0 | 3170.0 | 1460.0 | 1224.0 | 0.0 | 2364.0 | 5363.0 | 5692.0 | 0.0 | 7390.0 | 4422.0 | 0.0 | 0.0 | 0.0 | 0.0 | 0.0 | 0.0 | 0.0 | 0.0 | 4757.0 | 5154.0 | 0.0 | 0.0 | 0.0 | 0.0 | 7265.0 | 6400.0 | 0.0 | 0.0 | 1865.0 | 1196.0 | 23990.0 | 0.0 | 0.0 | 4335.0 | 7005.0 | 3652.0 | 0.0 | 0.0 | 0.0 | 0.0 | 0.0 | 0.0 | 0.0 | 0.0 | 0.0 | 0.0 | 0.0 | 0.0 | 0.0 | 0.0 | 0.0 | 0.0 | 0.0 | 0.0 | 0.0 | 0.0 | 0.0 | 0.0 | 0.0 | 0.0 | 0.0 | 0.0 | 0.0 | 3187.0 | 3026.0 | 0.0 | 0.0 | 0.0 | 0.0 | 0.0 | 3166.0 | 2788.0 | 0.0 | 0.0 | 1700.0 | 0.0 | 0.0 | 0.0 |
| 645 | 14106.0 | 14134.0 | 19101.0 | 12744.0 | 1450.0 | 4248.0 | 21691.0 | 10944.0 | 15112.0 | 12031.0 | 1773.0 | 10829.0 | 1916.0 | 22884.0 | 3538.0 | 1526.0 | 12612.0 | 20246.0 | 4813.0 | 2146.0 | 10710.0 | 0.0 | 3132.0 | 1582.0 | 1405.0 | 0.0 | 2501.0 | 5219.0 | 5522.0 | 0.0 | 6943.0 | 3965.0 | 0.0 | 0.0 | 0.0 | 0.0 | 0.0 | 0.0 | 0.0 | 0.0 | 4563.0 | 4852.0 | 0.0 | 0.0 | 0.0 | 0.0 | 6873.0 | 5907.0 | 0.0 | 0.0 | 1934.0 | 1130.0 | 22122.0 | 0.0 | 0.0 | 4231.0 | 6521.0 | 3466.0 | 0.0 | 0.0 | 0.0 | 0.0 | 0.0 | 0.0 | 0.0 | 0.0 | 0.0 | 0.0 | 0.0 | 0.0 | 0.0 | 0.0 | 0.0 | 0.0 | 0.0 | 0.0 | 0.0 | 0.0 | 0.0 | 0.0 | 0.0 | 0.0 | 0.0 | 0.0 | 0.0 | 2678.0 | 2481.0 | 0.0 | 0.0 | 0.0 | 0.0 | 0.0 | 2193.0 | 1894.0 | 0.0 | 0.0 | 1438.0 | 0.0 | 0.0 | 0.0 |
| 657 | 16997.0 | 14297.0 | 18185.0 | 12335.0 | 1476.0 | 3894.0 | 23454.0 | 11194.0 | 14967.0 | 12173.0 | 1948.0 | 10751.0 | 1819.0 | 24880.0 | 3225.0 | 1447.0 | 12937.0 | 21694.0 | 5013.0 | 1842.0 | 12309.0 | 0.0 | 3130.0 | 1227.0 | 1103.0 | 0.0 | 2403.0 | 5285.0 | 5378.0 | 0.0 | 7684.0 | 4675.0 | 0.0 | 0.0 | 0.0 | 0.0 | 0.0 | 0.0 | 0.0 | 0.0 | 5088.0 | 5362.0 | 0.0 | 0.0 | 0.0 | 0.0 | 7015.0 | 6562.0 | 0.0 | 0.0 | 1722.0 | 1105.0 | 24042.0 | 0.0 | 0.0 | 4432.0 | 7515.0 | 3246.0 | 0.0 | 0.0 | 0.0 | 0.0 | 0.0 | 0.0 | 0.0 | 0.0 | 0.0 | 0.0 | 0.0 | 0.0 | 0.0 | 0.0 | 0.0 | 0.0 | 0.0 | 0.0 | 0.0 | 0.0 | 0.0 | 0.0 | 0.0 | 0.0 | 0.0 | 0.0 | 0.0 | 3156.0 | 2978.0 | 0.0 | 0.0 | 0.0 | 0.0 | 0.0 | 2794.0 | 2382.0 | 0.0 | 0.0 | 1662.0 | 0.0 | 0.0 | 0.0 |
| 685 | 17940.0 | 14461.0 | 19923.0 | 13405.0 | 1482.0 | 3648.0 | 24696.0 | 10991.0 | 15735.0 | 12971.0 | 1988.0 | 11181.0 | 1802.0 | 25887.0 | 3165.0 | 1418.0 | 13306.0 | 23180.0 | 5545.0 | 2030.0 | 13158.0 | 0.0 | 3472.0 | 1269.0 | 1232.0 | 0.0 | 2753.0 | 5381.0 | 5099.0 | 0.0 | 8497.0 | 5258.0 | 0.0 | 0.0 | 0.0 | 0.0 | 1090.0 | 0.0 | 0.0 | 0.0 | 5772.0 | 6331.0 | 0.0 | 0.0 | 0.0 | 0.0 | 7934.0 | 7263.0 | 0.0 | 0.0 | 1897.0 | 1239.0 | 24731.0 | 0.0 | 0.0 | 5081.0 | 8051.0 | 3342.0 | 0.0 | 0.0 | 0.0 | 0.0 | 0.0 | 0.0 | 0.0 | 0.0 | 0.0 | 0.0 | 0.0 | 0.0 | 0.0 | 0.0 | 0.0 | 0.0 | 0.0 | 0.0 | 0.0 | 0.0 | 0.0 | 0.0 | 0.0 | 0.0 | 0.0 | 0.0 | 0.0 | 2662.0 | 2476.0 | 0.0 | 0.0 | 0.0 | 0.0 | 0.0 | 2466.0 | 2130.0 | 0.0 | 0.0 | 1434.0 | 0.0 | 0.0 | 0.0 |
| 737 | 12725.0 | 13133.0 | 18594.0 | 11868.0 | 1276.0 | 2971.0 | 23206.0 | 10343.0 | 14018.0 | 11584.0 | 1710.0 | 10225.0 | 1494.0 | 24859.0 | 2608.0 | 1419.0 | 12330.0 | 21324.0 | 5699.0 | 2080.0 | 13184.0 | 0.0 | 3470.0 | 1419.0 | 1186.0 | 0.0 | 2754.0 | 5564.0 | 5698.0 | 0.0 | 8562.0 | 5269.0 | 0.0 | 0.0 | 0.0 | 0.0 | 1061.0 | 0.0 | 0.0 | 0.0 | 5633.0 | 6112.0 | 0.0 | 0.0 | 0.0 | 0.0 | 8066.0 | 7484.0 | 0.0 | 0.0 | 1914.0 | 1141.0 | 23035.0 | 0.0 | 0.0 | 4880.0 | 8320.0 | 3344.0 | 0.0 | 0.0 | 0.0 | 0.0 | 0.0 | 0.0 | 0.0 | 0.0 | 0.0 | 0.0 | 0.0 | 0.0 | 0.0 | 0.0 | 0.0 | 0.0 | 0.0 | 0.0 | 0.0 | 0.0 | 0.0 | 0.0 | 0.0 | 0.0 | 0.0 | 0.0 | 0.0 | 2587.0 | 2464.0 | 0.0 | 0.0 | 0.0 | 0.0 | 0.0 | 2475.0 | 2078.0 | 0.0 | 0.0 | 1420.0 | 0.0 | 0.0 | 0.0 |
| 771 | 12132.0 | 10620.0 | 15856.0 | 8637.0 | 1046.0 | 2790.0 | 18087.0 | 9929.0 | 11179.0 | 9362.0 | 1538.0 | 8774.0 | 1617.0 | 19636.0 | 3002.0 | 1213.0 | 10176.0 | 17210.0 | 4906.0 | 1590.0 | 12112.0 | 0.0 | 3357.0 | 1319.0 | 1288.0 | 0.0 | 2287.0 | 6732.0 | 4507.0 | 0.0 | 4900.0 | 4387.0 | 0.0 | 1181.0 | 0.0 | 0.0 | 0.0 | 0.0 | 0.0 | 0.0 | 4417.0 | 4736.0 | 0.0 | 0.0 | 0.0 | 0.0 | 7279.0 | 6267.0 | 0.0 | 0.0 | 1810.0 | 0.0 | 19012.0 | 0.0 | 0.0 | 4128.0 | 6658.0 | 5673.0 | 0.0 | 0.0 | 0.0 | 0.0 | 0.0 | 0.0 | 0.0 | 0.0 | 0.0 | 0.0 | 0.0 | 0.0 | 0.0 | 0.0 | 0.0 | 0.0 | 0.0 | 0.0 | 0.0 | 0.0 | 0.0 | 0.0 | 0.0 | 0.0 | 0.0 | 0.0 | 0.0 | 1970.0 | 1717.0 | 0.0 | 0.0 | 0.0 | 0.0 | 0.0 | 1939.0 | 1684.0 | 0.0 | 0.0 | 1136.0 | 0.0 | 0.0 | 0.0 |
| 811 | 19285.0 | 11441.0 | 18000.0 | 8595.0 | 0.0 | 2064.0 | 23830.0 | 10514.0 | 11875.0 | 9129.0 | 1558.0 | 8816.0 | 1331.0 | 26797.0 | 2153.0 | 0.0 | 10614.0 | 26221.0 | 4648.0 | 0.0 | 13171.0 | 0.0 | 2867.0 | 0.0 | 0.0 | 0.0 | 1961.0 | 6458.0 | 3567.0 | 0.0 | 4948.0 | 4119.0 | 0.0 | 0.0 | 0.0 | 0.0 | 0.0 | 0.0 | 0.0 | 0.0 | 4401.0 | 4660.0 | 0.0 | 0.0 | 0.0 | 0.0 | 6552.0 | 6258.0 | 0.0 | 0.0 | 1512.0 | 0.0 | 25274.0 | 0.0 | 0.0 | 3903.0 | 6838.0 | 4905.0 | 0.0 | 0.0 | 0.0 | 0.0 | 0.0 | 0.0 | 0.0 | 0.0 | 0.0 | 0.0 | 0.0 | 0.0 | 0.0 | 0.0 | 0.0 | 0.0 | 0.0 | 0.0 | 0.0 | 0.0 | 0.0 | 0.0 | 0.0 | 0.0 | 0.0 | 0.0 | 0.0 | 1887.0 | 1792.0 | 0.0 | 0.0 | 0.0 | 0.0 | 0.0 | 2208.0 | 1909.0 | 0.0 | 0.0 | 1383.0 | 0.0 | 0.0 | 0.0 |
| 846 | 20089.0 | 11689.0 | 18488.0 | 8524.0 | 1078.0 | 2275.0 | 23724.0 | 10518.0 | 11807.0 | 9462.0 | 1632.0 | 8951.0 | 1460.0 | 25890.0 | 2373.0 | 1218.0 | 10744.0 | 24645.0 | 5097.0 | 1101.0 | 13857.0 | 0.0 | 3330.0 | 0.0 | 0.0 | 0.0 | 2158.0 | 6828.0 | 4205.0 | 0.0 | 4816.0 | 4125.0 | 0.0 | 0.0 | 0.0 | 0.0 | 0.0 | 0.0 | 0.0 | 0.0 | 4268.0 | 4461.0 | 0.0 | 0.0 | 0.0 | 0.0 | 7437.0 | 6543.0 | 0.0 | 0.0 | 1702.0 | 0.0 | 25472.0 | 0.0 | 0.0 | 4160.0 | 6777.0 | 5339.0 | 0.0 | 0.0 | 0.0 | 0.0 | 0.0 | 0.0 | 0.0 | 0.0 | 0.0 | 0.0 | 0.0 | 0.0 | 0.0 | 0.0 | 0.0 | 0.0 | 0.0 | 0.0 | 0.0 | 0.0 | 0.0 | 0.0 | 0.0 | 0.0 | 0.0 | 0.0 | 0.0 | 1757.0 | 1542.0 | 0.0 | 0.0 | 0.0 | 0.0 | 0.0 | 1926.0 | 1683.0 | 0.0 | 0.0 | 1158.0 | 0.0 | 0.0 | 0.0 |
| 883 | 17669.0 | 10669.0 | 16047.0 | 7893.0 | 0.0 | 1767.0 | 21938.0 | 9502.0 | 10546.0 | 8788.0 | 1415.0 | 8207.0 | 1166.0 | 24094.0 | 1880.0 | 0.0 | 10079.0 | 23019.0 | 4055.0 | 0.0 | 12457.0 | 0.0 | 2496.0 | 0.0 | 0.0 | 0.0 | 1601.0 | 5924.0 | 3467.0 | 0.0 | 3955.0 | 3189.0 | 0.0 | 0.0 | 0.0 | 0.0 | 0.0 | 0.0 | 0.0 | 0.0 | 3552.0 | 3689.0 | 0.0 | 0.0 | 0.0 | 0.0 | 6097.0 | 5598.0 | 0.0 | 0.0 | 1306.0 | 0.0 | 23543.0 | 0.0 | 0.0 | 3480.0 | 5651.0 | 4762.0 | 0.0 | 0.0 | 0.0 | 0.0 | 0.0 | 0.0 | 0.0 | 0.0 | 0.0 | 0.0 | 0.0 | 0.0 | 0.0 | 0.0 | 0.0 | 0.0 | 0.0 | 0.0 | 0.0 | 0.0 | 0.0 | 0.0 | 0.0 | 0.0 | 0.0 | 0.0 | 0.0 | 1649.0 | 1630.0 | 0.0 | 0.0 | 0.0 | 0.0 | 0.0 | 1953.0 | 1663.0 | 0.0 | 0.0 | 1193.0 | 0.0 | 0.0 | 0.0 |
| 897 | 13117.0 | 10776.0 | 18059.0 | 8801.0 | 0.0 | 2315.0 | 21957.0 | 10159.0 | 11687.0 | 9218.0 | 1511.0 | 8637.0 | 1380.0 | 23931.0 | 2570.0 | 1236.0 | 10466.0 | 21627.0 | 4927.0 | 0.0 | 13108.0 | 0.0 | 3308.0 | 0.0 | 0.0 | 0.0 | 1875.0 | 7199.0 | 4211.0 | 0.0 | 5210.0 | 4046.0 | 0.0 | 0.0 | 0.0 | 0.0 | 0.0 | 0.0 | 0.0 | 0.0 | 4247.0 | 4470.0 | 0.0 | 0.0 | 0.0 | 0.0 | 6863.0 | 6471.0 | 0.0 | 0.0 | 1635.0 | 0.0 | 24148.0 | 0.0 | 0.0 | 3788.0 | 6525.0 | 5957.0 | 0.0 | 0.0 | 0.0 | 0.0 | 0.0 | 0.0 | 0.0 | 0.0 | 0.0 | 0.0 | 0.0 | 0.0 | 0.0 | 0.0 | 0.0 | 0.0 | 0.0 | 0.0 | 0.0 | 0.0 | 0.0 | 0.0 | 0.0 | 0.0 | 0.0 | 0.0 | 0.0 | 1793.0 | 1557.0 | 0.0 | 0.0 | 0.0 | 0.0 | 0.0 | 2193.0 | 1841.0 | 0.0 | 0.0 | 1256.0 | 0.0 | 0.0 | 0.0 |
| 947 | 17890.0 | 11204.0 | 17351.0 | 8616.0 | 0.0 | 2210.0 | 23213.0 | 10542.0 | 12040.0 | 9832.0 | 1627.0 | 8764.0 | 1396.0 | 26274.0 | 2250.0 | 1217.0 | 10764.0 | 24457.0 | 4940.0 | 0.0 | 13056.0 | 0.0 | 3213.0 | 0.0 | 0.0 | 0.0 | 1853.0 | 6744.0 | 3712.0 | 0.0 | 5187.0 | 4032.0 | 0.0 | 0.0 | 0.0 | 0.0 | 0.0 | 0.0 | 0.0 | 0.0 | 4326.0 | 4539.0 | 0.0 | 0.0 | 0.0 | 0.0 | 6354.0 | 6150.0 | 0.0 | 0.0 | 1573.0 | 0.0 | 25473.0 | 0.0 | 0.0 | 3837.0 | 6542.0 | 5233.0 | 0.0 | 0.0 | 0.0 | 0.0 | 0.0 | 0.0 | 0.0 | 0.0 | 0.0 | 0.0 | 0.0 | 0.0 | 0.0 | 0.0 | 0.0 | 0.0 | 0.0 | 0.0 | 0.0 | 0.0 | 0.0 | 0.0 | 0.0 | 0.0 | 0.0 | 0.0 | 0.0 | 1796.0 | 1819.0 | 0.0 | 0.0 | 0.0 | 0.0 | 0.0 | 2368.0 | 2286.0 | 0.0 | 0.0 | 0.0 | 0.0 | 0.0 | 0.0 |
| 975 | 21074.0 | 10834.0 | 17559.0 | 8216.0 | 0.0 | 2037.0 | 23595.0 | 10258.0 | 11454.0 | 9116.0 | 1563.0 | 8362.0 | 1279.0 | 26436.0 | 2156.0 | 1133.0 | 10305.0 | 25634.0 | 4757.0 | 0.0 | 12671.0 | 0.0 | 3155.0 | 0.0 | 0.0 | 0.0 | 1822.0 | 6733.0 | 3820.0 | 0.0 | 4814.0 | 3953.0 | 0.0 | 0.0 | 0.0 | 0.0 | 0.0 | 0.0 | 0.0 | 0.0 | 3925.0 | 4315.0 | 0.0 | 0.0 | 0.0 | 0.0 | 6657.0 | 6006.0 | 0.0 | 0.0 | 1598.0 | 0.0 | 25093.0 | 0.0 | 0.0 | 3876.0 | 6329.0 | 5407.0 | 0.0 | 0.0 | 0.0 | 0.0 | 0.0 | 0.0 | 0.0 | 0.0 | 0.0 | 0.0 | 0.0 | 0.0 | 0.0 | 0.0 | 0.0 | 0.0 | 0.0 | 0.0 | 0.0 | 0.0 | 0.0 | 0.0 | 0.0 | 0.0 | 0.0 | 0.0 | 0.0 | 1867.0 | 2023.0 | 0.0 | 0.0 | 0.0 | 0.0 | 0.0 | 2425.0 | 2313.0 | 0.0 | 0.0 | 0.0 | 0.0 | 0.0 | 0.0 |
| 1087 | 19620.0 | 6974.0 | 14006.0 | 5832.0 | 0.0 | 1459.0 | 15926.0 | 6261.0 | 8377.0 | 6093.0 | 0.0 | 5634.0 | 0.0 | 20392.0 | 1491.0 | 0.0 | 6449.0 | 20004.0 | 3154.0 | 0.0 | 8003.0 | 0.0 | 2058.0 | 0.0 | 0.0 | 0.0 | 1518.0 | 5291.0 | 3599.0 | 0.0 | 3104.0 | 2422.0 | 0.0 | 0.0 | 0.0 | 0.0 | 0.0 | 0.0 | 0.0 | 0.0 | 2375.0 | 2655.0 | 0.0 | 0.0 | 0.0 | 0.0 | 4832.0 | 3708.0 | 0.0 | 0.0 | 1168.0 | 0.0 | 19555.0 | 0.0 | 0.0 | 2644.0 | 3767.0 | 5205.0 | 0.0 | 0.0 | 0.0 | 0.0 | 0.0 | 0.0 | 0.0 | 0.0 | 0.0 | 0.0 | 0.0 | 0.0 | 0.0 | 0.0 | 0.0 | 0.0 | 0.0 | 0.0 | 0.0 | 0.0 | 0.0 | 0.0 | 0.0 | 0.0 | 0.0 | 0.0 | 0.0 | 1213.0 | 0.0 | 0.0 | 0.0 | 0.0 | 0.0 | 0.0 | 1207.0 | 0.0 | 0.0 | 0.0 | 0.0 | 0.0 | 0.0 | 0.0 |
[unsupported chart]
OHT
